# Total Synthesis of the Antimycobacterial Natural Product Chlorflavonin and Analogs via a Late-Stage Ruthenium(II)-Catalyzed *ortho*-C(sp^2^)-H-Hydroxylation

**DOI:** 10.3390/ph15080984

**Published:** 2022-08-10

**Authors:** Alexander Berger, Talea Knak, Anna-Lene Kiffe-Delf, Korana Mudrovcic, Vinayak Singh, Mathew Njoroge, Bjoern B. Burckhardt, Mohanraj Gopalswamy, Beate Lungerich, Lutz Ackermann, Holger Gohlke, Kelly Chibale, Rainer Kalscheuer, Thomas Kurz

**Affiliations:** 1 Institute of Pharmaceutical and Medicinal Chemistry, Heinrich Heine University Düsseldorf, Universitätsstraße 1, 40225 Düsseldorf, Germany or; 2 Institute of Pharmaceutical Biology and Biotechnology, Heinrich Heine University Düsseldorf, Universitätsstraße 1, 40225 Düsseldorf, Germany; 3 South African Medical Research Council Drug Discovery and Development Research Unit, Department of Chemistry and Institute of Infectious Disease and Molecular Medicine, University of Cape Town, Rondebosch 7701, South Africa; 4 Drug Discovery and Development Centre (H3D), University of Cape Town, Rondebosch 7701, South Africa; 5 Institute of Clinical Pharmacy and Pharmacotherapy, Heinrich Heine University Düsseldorf, Universitätsstraße 1, 40225 Düsseldorf, Germany; 6 Institut für Organische und Biomolekulare Chemie, Georg-August-Universität Göttingen, Tammannstraße 2, 37077 Göttingen, Germany; 7 John-von-Neumann-Institute for Computing (NIC), Jülich Supercomputing Centre (JSC), Institute of Biological Information Processing (IBI-7: Structural Biochemistry), and Institute of Bio- and Geosciences (IBG-4: Bioinformatics), Forschungszentrum Jülich GmbH, 52428 Jülich, Germany

**Keywords:** *Mycobacterium tuberculosis*, natural product, flavonoid, acetohydroxyacid synthase inhibitor, *ortho*-C(sp^2^)-H-hydroxylation, 4*H*-chromen-4-one, chlorflavonin, antimycobacterial activity

## Abstract

The continuous, worldwide spread of multidrug-resistant (MDR) and extensively drug-resistant (XDR) tuberculosis (TB) endanger the World Health Organization’s (WHO) goal to end the global TB pandemic by the year 2035. During the past 50 years, very few new drugs have been approved by medical agencies to treat drug-resistant TB. Therefore, the development of novel antimycobacterial drug candidates to combat the threat of drug-resistant TB is urgent. In this work, we developed and optimized a total synthesis of the antimycobacterial natural flavonoid chlorflavonin by selective ruthenium(II)-catalyzed *ortho*-C(sp^2^)-H-hydroxylation of a substituted 3′-methoxyflavonoid skeleton. We extended our methodology to synthesize a small compound library of 14 structural analogs. The new analogs were tested for their antimycobacterial in vitro activity against *Mycobacterium tuberculosis* (*Mtb*) and their cytotoxicity against various human cell lines. The most promising new analog bromflavonin exhibited improved antimycobacterial in vitro activity against the virulent H37Rv strain of *Mtb* (Minimal Inhibitory Concentrations (MIC_90_) = 0.78 μm). In addition, we determined the chemical and metabolic stability as well as the p*K*_a_ values of chlorflavonin and bromflavonin. Furthermore, we established a quantitative structure–activity relationship model using a thermodynamic integration approach. Our computations may be used for suggesting further structural changes to develop improved derivatives.

## 1. Introduction

**Figure 1:**
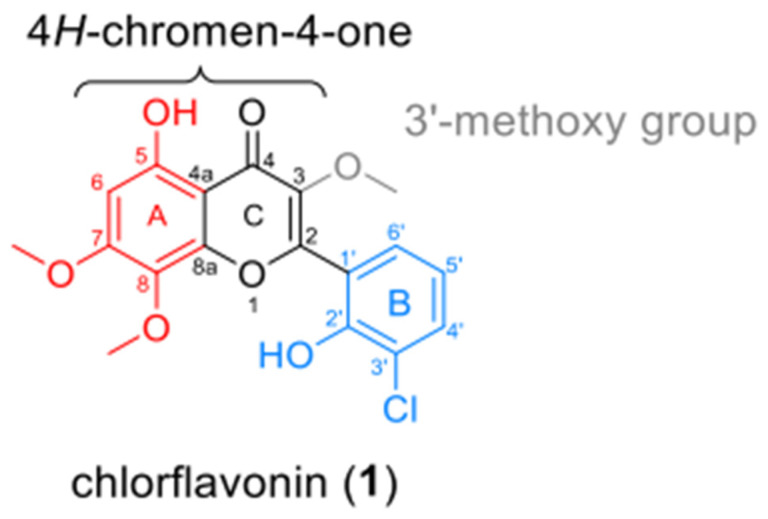
Structure and nomenclature of CF (**1**).

In 2020, the World Health Organization estimated that over two billion people were infected with tuberculosis (TB) caused by the bacterial pathogen *Mycobacterium tuberculosis* (*Mtb*). The first-line therapy for drug-susceptible *Mtb* strains has remained unchanged for 50 years, despite a long treatment duration and, in part, severe side effects. During this period, only three new anti-TB drugs with novel modes of action, bedaquiline, delamanid, and pretomanid, have been clinically approved. However, these three drugs are only approved for the treatment of multidrug-(MDR-) and extensively drug-resistant-(XDR-)TB [1,2,3]. Therefore, there is a strong need for the development of novel lead structures, and (pre)clinical candidates with novel mechanisms of action for inclusion in novel drug regimens that can contribute to shortening treatment and delaying resistance. The naturally occurring flavonoid chlorflavonin (CF, Figure 1) was first isolated in 1969 from *Aspergillus candidus*. CF’s antimycotic properties against, e.g., *Aspergillus amstelodami* und *Paecilomyces variotii* were reported in the same year [4]. In 1970, the structure of CF was resolved [5]. Recently, Rehberg et al. discovered CF’s strong in vitro growth inhibition against virulent *Mtb* H37Rv (MIC_90_ = 1.56 μM) and *Mtb* XDR clinical isolates. CF exhibited no cytotoxicity against the human fetal lung fibroblast cell line MRC-5 and monocyte cell line THP-1. For mono treatment with CF, a bacteriostatic effect was observed. In addition, CF demonstrated synergistic efficacy with delamanid and the first-line anti-TB drug isoniazid, including intracellular activity against infected human macrophages. CF specifically inhibits the large catalytic subunit IlvB1 of the mycobacterial acetohydroxyacid synthase (AHAS), which is the first common enzyme in the de novo biosynthetic pathway of essential branched-chain amino acids (BCAA) and pantothenic acid [6,7,8,9,10,11,12,13]. The inhibition of the mycobacterial AHAS through chlorflavonin causes combined auxotrophies of BCAA and pantothenic acid [14]. In addition, the treatment of *Mtb*-infected mice with the AHAS inhibitor sulfometuron methyl, which is approved as a herbicide, led to a significantly reduced proliferation of the pathogen in the host’s lungs [15]. In the context of vaccine research, it was shown that BCAA-auxotrophic mutants of intracellular pathogens exhibited significantly reduced virulence in vivo [16]. The fact that AHAS is present in *Mtb* but not in the human host makes it a promising target for developing novel antituberculosis drugs [17].

**Scheme 1:**
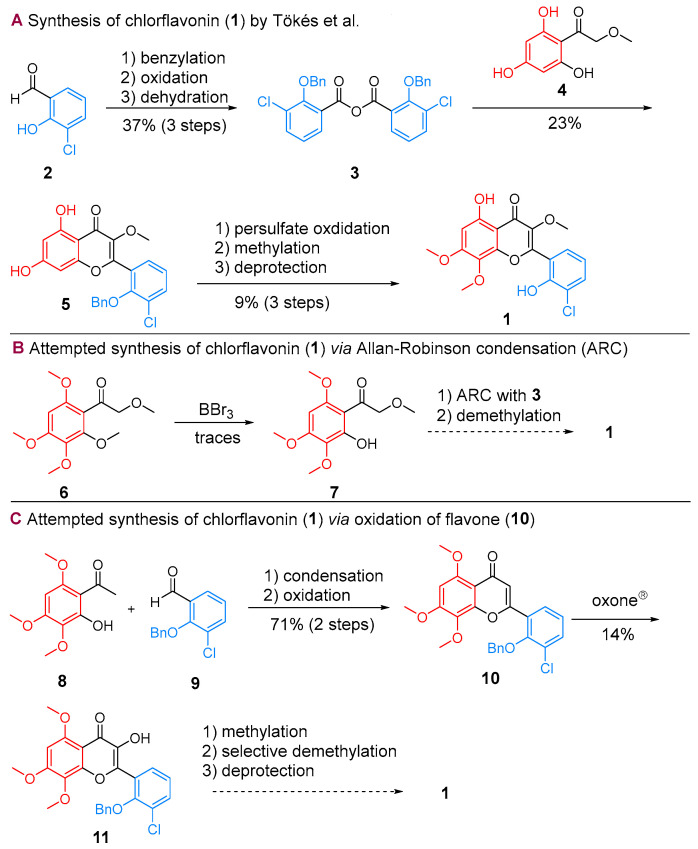
Synthetic approaches towards chlorflavonin (**1**).

None of the currently approved TB drugs inhibit AHAS nor any enzyme in the BCAA biosynthetic pathway [14]. CF’s novel mechanism of action, structure, and potent antimycobacterial in vitro activity make it a promising lead structure for developing a new class of anti-TB drugs. The eponymous chlorine substituent on the B-ring adjacent to the phenolic hydroxyl group is particularly noteworthy and unusual for a flavonoid [18,19]. In 1980, Tökés et al. described the only published synthesis of CF through an Allan–Robinson condensation of 2-(benzoyloxy)-3-chlorobenzoic anhydride (**3**) with a hydroxyacetophenone derivative 4 in a total yield of only 0.79% over seven steps (Scheme 1A) [20].

## 2. Results and Discussion

### 2.1. Synthesis of CF

**Scheme 2:**
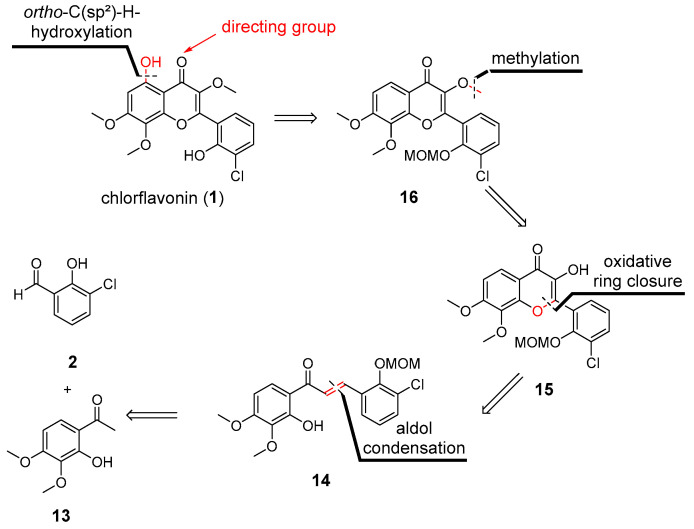
Retrosynthetic approach.

**Scheme 3:**
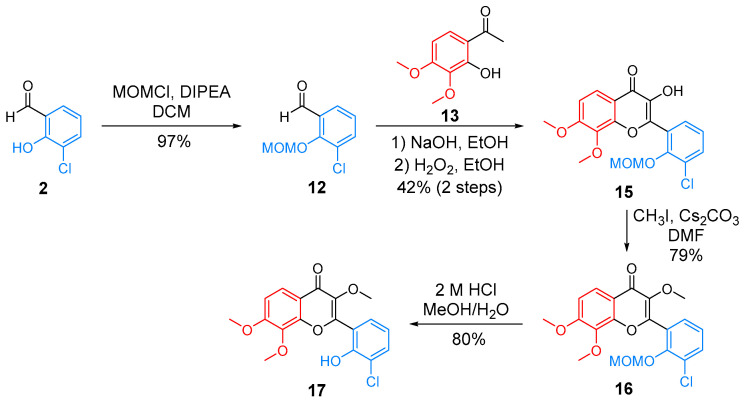
Synthesis of 3′-methoxyflavonol **17**.

In a first synthetic attempt, we tried to avoid the occasionally unfavorable Elbs persulfate oxidation by using the 2′-hydroxyacetophenon **7** with the natural substitution pattern of the A-ring [21]. Using this approach adapted from Tökés et al. [20], we planned to circumvent the difficult selective methylation of the phenolic hydroxyl groups in the 7- and 8-position (Scheme 1A). While intermediate 6 was successfully synthesized, the selective demethylation of 6 with BBr_3_ to provide 1-(2-hydroxy-3,4,6-trimethoxyphenyl)-2-methoxyethan-1-one (**7**) in sufficient yield was not feasible (Scheme 1B, see Supplementary Materials for details). In our second attempt, flavone **10** was synthesized by a Claisen–Schmidt condensation of the substituted 2′-hydroxyacetophenon **8** and the salicylic aldehyde derivative **9**, followed by oxidative cyclo-dehydration of the chalcone intermediate with a catalytic amount of iodine in dimethyl sulfoxide (DMSO). Unfortunately, the oxidation of flavone **10** with dimethyldioxirane (DMDO), oxone^®^, or *tert*-butylhydroperoxid (TBHP) resulted in poor yields (Scheme 1C, see Supplementary Materials for details). To introduce the important phenolic hydroxyl group of the A-ring immediately and to forego selective demethylation in a separate step, the commonly used Algar–Flynn–Oyamada (AFO) reaction was employed to build the flavonol skeleton. However, the substrate scope of the AFO reaction was limited due to the competing formation of aurones when using substrates with electron-donating groups [21,22,23]. Based on this limitation, we planned to generate the essential substitution pattern of the A-ring via a transition metal-catalyzed *ortho*-C(sp^2^)-H-functionalization using the carbonyl oxygen of the 4-chromanone moiety as the coordinating directing group in the last step (Scheme 2). Therefore, the synthesis of the 3′-methoxyflavonol **16** should take place by methylation of the flavonol **15**, which can be synthesized in advance by Claisen–Schmidt condensation of the commercially available starting materials **2** and **13**. The oxidative ring closure of the chalcone intermediate should then be initiated with alkaline hydrogen peroxide in an ethanolic solution in a sequential one-pot process. Starting from 3-chloro-2-hydroxybenzaldehyde (**2**), the methoxymethyl ether (MOM) protecting group was introduced to the phenolic hydroxyl group by nucleophilic substitution with chloromethyl methyl ether in the presence of *N*,*N*-diisopropylethylamine (DIPEA) with an excellent yield of 97% (Scheme 3).

Next, the resulting MOM-protected salicylaldehyde **12** was condensed with the commercially available 2′-hydroxyacetophenone **13** by a base-mediated Claisen–Schmidt condensation in ethanolic solution. Afterward, hydrogen peroxide was added to initiate the oxidative Algar–Flynn–Oyamada ring closure to provide flavonol **15** with a moderate yield of 42% on a multigram scale. Effective cooling, a slow addition rate of hydrogen peroxide, and sufficient dilution of the reaction mixture were critical for avoiding side products and simplifying the purification process (Scheme 3). Next, the enolic hydroxyl group of flavonol **15** was methylated with iodomethane in the presence of Cs_2_CO_3_ in dimethylformamide (DMF). The MOM protecting group of 3′-methoxyflavonol **16** was then removed by acidolysis with methanolic hydrochloric acid at 50 °C to give the deprotected flavonoid **17** with a good yield. It was unnecessary to add a scavenger to deactivate in situ-generated formaldehyde, since no significant side reactions were observed (Scheme 3).

In the final step, we studied the introduction of the essential phenolic hydroxyl group in position 5 of the A-ring by a selective transition metal-catalyzed *ortho*-C(sp^2^)-H-hydroxylation using the carbonyl oxygen of the 4*H*-chromen-4-one skeleton as a coordinating directing group. In the last two decades, significant progress has been made for C-H-functionalization [24,25,26], especially for substrates with weakly coordinating directing groups such as the carbonyl oxygen of acetophenones and chromones [26,27,28,29,30,31,32,33] An extensive catalyst, oxidant, solvent, and temperature screening was performed to optimize our method. For this purpose, the conversion and selectivity of the *ortho*-C(sp^2^)-H-hydroxylation were monitored by HPLC-UV/Vis (Table 1). Inspired by the work of Shan et al. and others, we first studied palladium(II)-catalysts in combination with PhI(TFA)_2_ as the terminal oxidant in 1,2-dichlorethane (DCE) [34]. However, these reaction conditions provided only trace amounts of CF, and in the case of longer reaction times and an excess of oxidant, the substrate decomposed (entries 1–2). Replacing DCE with a mixture of trifluoroacetic acid (TFA) and trifluoroacetic anhydride (TFAA) (9:1, *v*/*v*) resulted in a higher conversion rate and moderate selectivity. Notably, reversing the solvent ratio significantly increased conversion and improved selectivity (entries 3–4). By combining [RuCl_2_(*p*-cymene)]_2_ as a catalyst and selectfluor as an oxidizing agent, the formation of byproducts could be suppressed to such an extent that purification by silica gel chromatography or recrystallization was successfully applied (entries 5–6). The addition of a silver(I) additive was beneficial because of its role as a halogen scavenger. Silver-cations abstract chloride from the neutral dimeric catalyst and form two monomeric, cationic catalyst species, which can interact with the partially negatively charged oxygen in a stronger fashion [29,35]. While an increased number of side reactions was observed with 2.0 equivalents of the oxidizing agent, virtually no side reaction occurred with a slight stoichiometric excess of 1.1 equivalents (entries 7–8). At the ideal reaction temperature of 80 °C, good conversions and superior selectivity were achieved, while only trace amounts of 1 were detected at 60 °C. In contrast, complete conversion of the starting material was observed at 120 °C, as well as the formation of several side products, necessitating purification with RP-flash chromatography (entries 9–10). Furthermore, no significant increase in conversion was observed when the reaction was carried out under argon atmosphere, nor with the continuous addition of the oxidants or without separate previous activation of the catalyst through the silver additive (see Supplementary Materials for complete optimization studies). Initially, the *ortho*-C(sp^2^)-H-hydroxylation yielded the 2,2,2-trifluoroacetate intermediate **18**, which was converted into the desired product 1 through methanolysis. The structure of chlorflavonin was elucidated via ^1^H-and ^13^C-NMR, which agrees with the spectral data published and discussed by Rehberg et al. [14].

### 2.2. Lead Optimization of CF

**Scheme 4:**
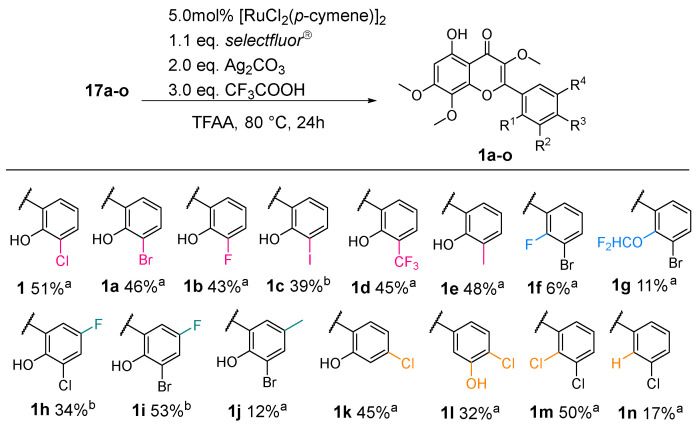
Scope of ortho-C(sp^2^)-H-hydroxylation. ^a^ Reactions were performed on a 1.0 mmole scale. ^b^ 0.3–0.8 mmole reaction scales.

After establishing the optimal reaction conditions, we designed a small compound library of CF analogs for preliminary structure–activity relationship (SAR) studies. Previously, we published a homology model of the *Mtb* H37Rv IlvB1 catalytic subunit of AHAS using the *Saccharomyces cerevisiae* and *Arabidopsis thaliana* (PDB ID 1T9C and 1YBH) AHAS proteins as templates [14]. Subsequent docking studies performed with Glide and AutoDock identified potential interactions between CF and amino acid residues within the putative active site of IlvB1, which provided a rational approach for the design of the analogs. Therefore, in this first series of analogs, we focused on modifications to the B-ring. Since the lipophilic chlorine substituent might interact with amino acid side chains inside a hydrophobic side pocket, we replaced the chlorine substituent with bioisosteric moieties and lipophilic substituents, e.g., Br and CF_3_ [14]. In addition, we wanted to increase the acidity of the phenolic hydroxyl group of the B-ring to facilitate its deprotonation and strengthen the possible salt bridge to Lys197. To analyze the importance of the 2′-hydroxy group, we replaced it with the bioisosteric difluoromethoxy group and a chlorine or fluorine substituent [36,37,38]. The salicylic aldehyde starting materials and several MOM-protected salicylic aldehydes were purchased or synthesized according to the literature [39,40,41,42,43,44,45]. Analogs **17a**–**p** were synthesized according to Scheme 3. For the preparation of **1h**, an additional step was needed. According to a procedure of Li et al. for difluromethylation of phenols [46], the 2′-hydroxy group of intermediate **17b** was treated with aqueous KOH solution in dichloromethane. Dropwise addition of (bromodifluormethyl)trimethylsiliane as difluorocarbene precursor gave intermediate **19** with a yield of 78%. The final *ortho*-C(sp^2^)-H-hydroxylation of **17a**–**p** afforded the CF analogs **1a**–**p** with yields of 6–53% (Scheme 4).

The SAR for the B-ring was quite narrow, and even small structural changes resulted in a loss of antimycobacterial activity. This activity loss was observed for the bioisosteric replacement of the 2′-hydroxy group with potential bioisosteric groups (**1f**,**g**), the introduction of a fluorine or a methyl substituent in the 5′-position of the B-ring (**1h**–**j**), and the variation in the substitution pattern (**1k**–**o**). An explanation for the inactivity of these analogs could be their poor water solubility. For a more comprehensive assessment of the antimycobacterial activity, a better understanding of the biological functions of the different mycobacterial AHAS isoenzymes is needed. For this purpose, we are currently developing a new robust AHAS enzyme assay.

### 2.3. Evaluation of Selected Physicochemical Properties, Microsomal Stability, and Computations

For further preclinical profiling of CF and BF, selected physicochemical properties (e.g., water solubility), chemical and microsomal metabolic stability were determined. The aqueous solubility of both compounds was determined to be <5 μM at a pH of 7.4 using a miniaturized shake-flask method (Table 2). While the chemical stability of CF and BF in a phosphate buffer at pH 2.0 and 7.4 was excellent (99% drug content after 48 h), the metabolic stability in human liver microsomes was moderate, and both compounds can be classified as high extraction drugs (hepatic extraction ratio E_H_ ≥ 0.72) (Table 2).

To investigate the protonation state of CF and BF in physiological media (pH = 7.4) and these compounds’ ability to form the proposed salt bridge with Lys197, the p*K*_a_ values were determined by ^1^H-NMR titration (see Supplementary Data File; Figures S1–S4). For determination of the p*K*_a_ of the 5-hydroxy group, the shift of the singlet at 6.65 ppm, the triplet at 7.01 ppm for the 2′-hydroxyl group, and both double duplets at 7.42 and 7.59 ppm were used.

Furthermore, seven structurally diverse chlorflavonin derivatives (**1**, **1a**, **1b**, **1c**, **1d**, **1f**, **1i**) with substitutions at the B-ring were chosen to establish a quantitative structure–activity relationship model using the thermodynamic integration (TI) approach [49,50], as implemented in FEW [51] of Amber21 (see Supplementary Materials Data File) [52]. The computed relative free energies (ΔΔ*G*) were generally in qualitative agreement with the changes in the minimal inhibitory concentrations (MIC_90_) (see Supplementary Materials Data File; Figure S7). This suggests that changes in MIC_90_ were predominantly determined by differences in the derivatives’ affinities. Furthermore, such computations may suggest further structural changes to obtain more active chlorflavonin derivatives.

## 3. Materials and Methods

### 3.1. Chemistry

The syntheses of chlorflavonin and chlorflavonin analogs are described in detail in the Supplementary Materials Data File. Commercially available reagents and solvents were purchased from Apollo Scientific, Sigma-Aldrich, TCI, BLDpharm, Carbolution, ABCR GmbH, Acros Organics, or Alfa Aesar and were used without further purification. Dry solvents were purchased from Acros Organics. Analytical thin-layer chromatography was performed using silica gel 60 F254 aluminium plates. Compound spots were visualized either by UV light (254 nm) or by staining with a solution of 1% FeCl_3_ in ethanol. Flash chromatography was performed on CombiFlash^®^ Rf 200 using RediSep™ Rf-columns. ^1^H-, ^13^C-, and ^19^F-NMR spectra were recorded with Bruker Avance III—300, Bruker Avance III—600, or Bruker Avance DRX—500 spectrometers. ^1^H- and ^13^C-NMR signals were calibrated to the residual proton and carbon resonance of the solvent: CDCl_3_ (^1^H-NMR δ = 7.26 ppm, ^13^C-NMR δ = 77.2 ppm), DMSO (^1^H-NMR δ = 2.50 ppm, ^13^C-NMR δ = 39.5 ppm). The following abbreviations were used to describe peak splitting patterns when appropriate: s = singlet, d = doublet, t = triplet, q = quartet, h = hextet, m = multiplet, dd = doublet of doublet, td = doublet of triplet, ddd = doublet of doublet of doublet and brs = broad singlet. Coupling constants, J, were reported in the Hertz unit (Hz). ESI-MS data were recorded with UHR-QTOF maXis 4G. Melting points were measured on a Büchi M 565 instrument and were not corrected. Reverse-phase high-performance liquid chromatography data were measured with Varian ProStar 210 with a Phenomenex Luna C-18 (2) particle size 5 μm (250 × 4.6 mm) column. The detection took place with the UV detector Varian ProStar 330 at 220–254 nm, and eluents water/acetonitrile with 0.1% TFA were used.

### 3.2. Determination of Minimal Inhibitory Concentration (MIC)

To characterize the structure–activity-relationship, the MIC assay was employed as previously described. Briefly, *M. tuberculosis* H37Rv cells were precultured in Middlebrook 7H9 liquid medium supplemented with 0.5% (*v*/*v*) glycerol, 0.05% (*v*/*v*) tyloxapol, and 10% (*v*/*v*) ADS (0.81% NaCl, 5% BSA and 2% dextrose) at 37 °C to an OD_600 nm_ of 0.5–0.8. Cells were diluted with fresh medium and seeded into 96-well plates to yield a final density of 10^5^ cells in 100 µL medium per well. The compounds were tested in two-fold serial dilutions with a final concentration ranging from 100 to 0.05 µM. The microtiter plates were incubated at 37 °C, 5% CO_2_, and 80% humidity for 5 days. Then 10 µL of a 100 µg/mL resazurin solution were added to each well and incubated for 16 h at room temperature. After fixation of the bacteria for 30 min with a final concentration of 5% (*v*/*v*) formalin, the fluorescence was measured in a TECAN microplate reader (excitation of 540 nm, emission of 590 nm). The percentage of growth was calculated in comparison to dimethyl sulfoxide (DMSO)-treated cells (=100% growth) and the sterile control (=0% growth). All experiments were performed in triplicates.

### 3.3. Determination of Cytotoxcity

For the determination of the cytotoxicity of the compounds, different human cell lines were used. The human fetal lung fibroblast cell line MRC-5 (American Type Culture Collection) was incubated in Eagle’s Minimum Essential Medium (EMEM) containing 1% (*v*/*v*) Na-pyruvate, whereas the human embryonic kidney cell line HEK-293 (CLS Cell Lines Service GmbH) was cultivated in EMEM supplemented with 2 mM l-glutamine, 1% (*v*/*v*) non-essential amino acids, and 1 mM sodium pyruvate. The human monocyte cell line THP-1 (Deutsche Sammlung von Mikroogranismen und Zellkulturen GmbH) was cultured in RPMI 1640 medium. All media were supplemented with 10% (*v*/*v*) heat-inactivated fetal bovine serum (FBS).

The cell densities were quantified using a hemocytometer and then adjusted to 10^6^ cells/mL. Next, 100 µL per well were seeded in 96-well microtiter plates to yield an inoculation cell density of 5 × 10^5^ cells per well. Compounds were added employing two-fold serial dilutions resulting in final concentrations ranging from 100 to 0.78 µM. Cells were incubated for two days at 37 °C and 5% CO_2_ in a humidified atmosphere. Subsequently, 10 µL of a 100 µg/mL resazurin solution was added to each well, mixed thoroughly, and incubated for an additional 3–4 h. Fluorescence was measured in a TECAN microplate reader (excitation of 540 nm, emission of 590 nm). The percentage of growth was calculated with respect to DMSO (100% growth) and Triton X-100 (0% growth) controls.

### 3.4. Evaluation of Solubility

Solubility was measured at pH 7.4 using an adapted miniaturized shake-flask method in 96-well plate format [53,54]. Briefly, 4 μL of a 10 mM stock in DMSO was added to a 96-well plate and evaporated using a GeneVac^®^ system. Phosphate buffer pH 7.4 was then added to the wells, and the plate was incubated for 24 h at 25 °C with shaking. At the end of this incubation, the samples were centrifuged at 3500 *g* for 15 min then transferred to an analysis plate. A calibration curve in DMSO for each sample between 10 and 220 μM was prepared and included in the analysis plate. Analysis was then performed by HPLC-DAD, and the solubility of each sample was determined from the corresponding calibration curve. Reserpine and hydrocortisone were used as positive controls and treated similarly.

### 3.5. Evaluation of Compound Chemical Stability

Stability testing was performed in phosphate buffer pH 2.0 and 7.4 (prepared according to Ph. Eur. 10) at 20 °C over a period of 48 h. Therefore, 0.4 mg of substance was dissolved in Tween^®^ 20 und ethanol (7/3 *v*/*v*) and diluted with the respective phosphate buffer. The solution was shaken with an IKA KS 260 basic (250 min^−1^) at 25 °C for 48 h. Analysis was performed after 1, 3, 6, 24, and 48 h by comparing the area under the curve (A.U.C.), which was measured with HPLC-UV at 254 nm (*n* = 1).

### 3.6. Evaluation of Compound Metabolic Stability

The metabolic stability assay was performed in Human Liver Microsomes (HLM) using a single-point metabolic stability assay. Briefly, the compounds were incubated at 1 μM in 0.4 mg/mL human mixed-gender liver microsomes (Xenotech, Kansas City, KS, USA, pool of 50) for 30 min at 37 °C. Reactions were quenched by adding ice-cold acetonitrile containing internal standard. The samples were then centrifuged and analyzed by LC-MS/MS for the disappearance of the parent compound. Half-life, clearance, and hepatic excretion ratios were determined using standard equations [47,48]. Propranolol and midazolam were used as positive controls and treated similarly.

### 3.7. Determination of Compound pK_a_ Values

NMR experiments were performed at 298 K on a Bruker Avance III HD spectrometer operating at 600 MHz equipped with 5 mm triple resonance TCI (^1^H, ^13^C, ^15^N) cryoprobes and shielded z-gradients. For the p*K*_a_ value determination, 23 samples of 200 µM concentration of CF and BF were prepared with pH ranges from 2 to 13 (pH in 0.5 log unit steps) in 50 mM sodium phosphate, 100 mM sodium chloride, 10% (*v*/*v*) DMSO-d_6_. 1D ^1^H-NMR experiments were performed using 128 or 256 scans for each sample, and the data were processed and analyzed by the TopSpin 3.2 software (TopSpin, v3.2, Bruker BioSpin GmbH, Rheinstetten, Germany). Sodium 2,2-dimethyl-2-silapentane-5-sulfonate (DSS) was used for chemical shift referencing. ^1^H chemical shift values were extracted for the reporter protons using the TopSpin software (see Supplementary Materials Data File Figures S1 and S3). The p*K*_a_ values were calculated using the chemical shifts of the protonated and unprotonated forms and their corresponding mole fraction values applying the Henderson–Hasselbalch equation as explained by Gift et al. [55]. The data were plotted with the Origin software (Origin Pro 2019, v9.6.0.172, OriginLab Corporation, Northampton, MA, USA) (see Supplementary Data File Figures S2 and S4).

### 3.8. Analysis of a Quantitative Structure-Activity Relationship Model

Chlorflavonin derivates were prepared with the LigPrep module of the Schrödinger suite [56] and subsequently aligned with align_ligands of the Schrödinger suite to chlorflavonin docked into the homology model of the catalytic subunit of human AHAS [14]. The homology model [14] was prepared for calculations with the Protein Preparation Wizzard module of the Schrödinger suite. Files for relative free energy computations and analyses were prepared with the TI module of FEW [51], and AMBER version 21.1 [52] was used for simulations. The RESP charge model [57] was chosen for computing partial charges of the ligands.

Ligands in water and ligand–protein complexes in water were minimized with a three-step procedure, each with 2000 steps of steepest descent and 1000 steps of conjugate-gradient minimization in the presence of 25 kcal mol^−1^ A^−2^, 5 kcal mol^−1^ A^−2^, and no restraints on the solute, respectively. Subsequently, three replicas of each system were heated from 100 K to 300 K in the NVT (constant temperature and constant volume) ensemble during 50 ps of MD simulations followed by an adjustment of the density in the NPT (constant temperature and constant pressure) ensemble during a 50 ps MD run.

The transition map was based on Kruskal’s algorithm [58] with a modification introduced to close thermodynamic cycles for convergence control. As “cost” of the edges, 10^3^ × (2—TanimotoCombo) was calculated, where the TanimotoCombo score was computed with the ROCS module of OpenEye [56,59]. Production phase MD simulations were carried out at 300 K in the NVT ensemble for 9 *λ* windows, *λ* = 0.1, 0.2, …, 0.9, respectively, with lengths of 10 ns per *λ* window for ligands in solvent and 30 ns per *λ* window for complexes in solvent. The impact of the simulation time per *λ* window (10 ns and 30 ns) on the precision of the computations in the case of complex simulations was estimated by calculating the standard deviation over three replicas (Figure S5). The convergence of the computations was confirmed in three ways: First, the precision of average d*V*/d*λ* for each *λ* window was determined by employing the Student’s distribution for each replica separately as implemented in the FEW workflow (convergence_check_method 2) [51]; second, the average d*V*/d*λ* values were checked with respect to their consistency across the three replicas (Figure S6); third, the cycle-closure hysteresis of closed thermodynamic cycles was computed (Table S3).

### 3.9. Solubility and Partition Coefficient Predictions

QikProp [60] of the Schrödinger suite (version 6.7), Schrödinger, LLC, New York, NY, USA, 2015 was used to compute the solubility (QPlog*S*) and octanol/water partition coefficient (QPlog*P*_o/w_) for chlorflavonin derivates included in relative free energy computations (Table S4).

## 4. Conclusions

In conclusion, we developed an efficient five-step synthesis of the antimycobacterial natural flavonoid chlorflavonin (**1**) and 14 structural analogs, demonstrating the broad applicability of our method. The overall yield of CF (13.5%) was significantly improved compared to the original synthesis by Tökés et al. The SAR for the B-ring was surprisingly narrow, as small structural changes resulted in a loss of antimycobacterial activity. We will therefore extend the future optimization to rings A and C. The most active compound, BF, exhibited submicromolar antimycobacterial activity with no cytotoxicity toward human cells. The bromine substituent appeared to fill the proposed lipophilic sub-pocket ideally. While the chemical stability of CF and BF was excellent, the metabolic stability needs improvement through future lead optimization. The initially derived SARs should encourage and guide the synthesis of further analogs. For a successful and more comprehensive structure optimization of CF and BF, we are currently developing a robust AHAS enzyme assay. In addition, our quantitative structure-activity relationship model will be further developed to effectively support this lead optimization program.

## Figures and Tables

**Table 1:** Optimization of *ortho*-C(sp^2^)-H-hydroxylation of 3′-methoxyflanonol **17**.

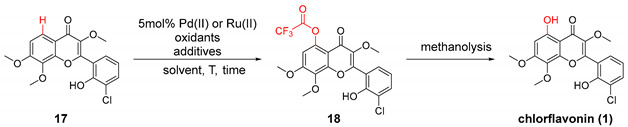							
Entry ^a^	Catalyst	Oxidant (Equiv.)	Additive (Equiv.)	Solvent (0.07 M)	T (°C)	Time (h)	Conversion (%)
**1**	Pd(TFA)_2_	PhI(TFA)_2_ (1.2)	-	DCE	80	8	trace
**2**	Pd(TFA)_2_	PhI(TFA)_2_ (2.0)	-	DCE	80	16	decomp.
**3**	Pd(TFA)_2_	PhI(TFA)_2_ (1.2)	-	9:1 TFA/TFAA	80	8	48
**4**	Pd(TFA)_2_	PhI(TFA)_2_ (1.2)	-	1:66 TFA/TFAA ^b^	80	8	52
**5**	[RuCl_2_(*p*-cymene)]_2_	PhI(TFA)_2_ (1.5)	Ag_2_CO_3_ (1.5)	1:66 TFA/TFAA ^b^	80	24	43
**6**	[RuCl_2_(*p*-cymene)]_2_	*selectfluor* (1.5)	Ag_2_CO_3_ (1.5)	1:66 TFA/TFAA ^b^	80	16	53
**7**	[RuCl_2_(*p*-cymene)]_2_	*selectfluor* (2.0)	Ag_2_CO_3_ (2.0)	1:66 TFA/TFAA ^b^	80	16	61
**8**	[RuCl_2_(p-cymene)]_2_	*selectfluor* (1.1)	Ag_2_CO_3_ (2.0)	1:66 TFA/TFAA ^b^	80	24	72
**9**	[RuCl_2_(*p*-cymene)]_2_	*selectfluor* (1.5)	Ag_2_CO_3_ (1.5)	1:66 TFA/TFAA ^b^	60	16	trace
**10**	[RuCl_2_(*p*-cymene)]_2_	*selectfluor* (1.2)	Ag_2_CO_3_ (1.2)	1:66 TFA/TFAA ^b^	120	2	74

^a^ Reaction conditions: Flavone 8 (0.5 mmole, 1.0 equiv.), 5 mol% catalyst, 1.1–2.0 equiv oxidant, 1.2–2.0 equiv additive. ^b^ 3.0 equiv. TFA were used.

**Table 2:** Determination of physicochemical properties.

	CF	BF
Water solubility at pH 7.4 *^a^*	<5 μM	<5 μM
p*K*_a1_ (2′-OH) *^b^* p*K*_a2_ (5-OH) *^b^*	6.80 ± 0.07 10.40 ± 0.03	6.74 ± 0.04 10.30 ± 0.04
remaining after 30 min *^c^*	48%	60%
CL_int, app_ *^d^*	76.3 mL/min/mg	53.4 mL/min/mg
E_H_ *^d^*	0.78	0.72

*^a^* 1 mg/mL compound was dissolved in phosphate buffer pH 7.4. The solubility was determined using the shake-flask method and measured with HPLC at 254 nm after 4 and 24 h. *^b^* p*K*_a_ values were determined by ^1^H-NMR titration. *^c^* The compounds were incubated at 1 μM in human liver microsomes (0.4 mg/mL) for 30 min at 37 °C. The samples were analyzed by LC-MS/MS for the disappearance of the parent compound. *^d^* Predicted in vivo intrinsic clearance (CL_int, app_) and hepatic extraction ratio (E_H_) were determined using standard equations established by Di et al. and Obach et al. [47,48].

## Data Availability

Data are contained within the article and Supplementary Materials.

## Supplementary Materials

The following Supplementary data files can be downloaded at: https://www.mdpi.com/article/10.3390/ph15080984/s1. References [55,56,57,58,59,60,61] are cited in the Supplementary Materials. In this file, we describe the synthesis, characterization, and analysis of chlorflavonin **1** and chlorflavonin analogs **1a**–**n**. Spectral copies of ^1^H-, ^13^C-, and ^19^F-NMR data of chlorflavonin **1** and chlorflavonin analogs **1a**–**n**. Table S1. Literature for known compounds. Table S2. Reaction optimization. Table S3. Relative free energy of binding calculated with the FEW free energy workflow. All values of cycle closure hysteresis are below the threshold of chemical accuracy (1 kcal mol^−1^). Figure S1: ^1^H-NMR-Spectra of chlorflavonin’s aromatic protons (6–8 ppm) at pH of 2.0–12.5. A–D are the four aromatic protons. Chemical shift in ppm (parts per million). Figure S2: Titration curves (chemical shift in ^1^H-NMR spectra vs. pH value) of chlorflavonin’s four aromatic protons plotted with Origin software (OriginLab Corporation, Northampton, MA, USA). Figure S3: ^1^H-NMR-Spectra of bromflavonin’s aromatic protons (6–8 ppm) at pH of 2.0–12.5. A–D are the four aromatic protons. Chemical shift in ppm (parts per million). Figure S4: Titration curves (chemical shift in ^1^H-NMR spectra vs. pH value) of bromflavonin’s four aromatic protons plotted with Origin software (OriginLab Corporation, Northampton, MA, USA). Table S4. Predicted solubility and octanol/water partition coefficient computed with QikProp for chlorflavonin derivatives included in the relative free energy computations. Figure S5. Average dV/dλ over three replicas of free energy calculations of complexes with respect to the simulation time (10 ns per λ step and 30 ns per λ step). Error bars denote the standard deviation from the three replicas. Figure S6. Ensemble-averaged d*V*/d*λ* after 10 ns per *λ* step (ligand in solvent) and 30 ns per *λ* step (complex in solvent) of sampling time for transitions of chlorflavonin derivatives. The standard error of the mean in all cases was < 0.1 kcal mol^−1^. Figure S7. Comparison of predicted relative free energy of binding (ΔΔ*G* predicted) with the corresponding relative free energy of binding calculated from difference in minimal inhibitory concentration (ΔΔ*G* calculated from MIC_90_) for the transitions V0 → V1 shown on the x-axis.
