# Supplementary material for: Total Synthesis of the Antimycobacterial Natural Product Chlorflavonin and Analogs via a Late-Stage Ruthenium(II)-Catalyzed ortho-C(sp2)-H-Hydroxylation
Source: Pharmaceuticals (Basel). 2022 Aug 10;15(8):984. doi: 10.3390/ph15080984 (PMC9415896; doi:10.3390/ph15080984)
Supplement: Supplementary file 1 [file pharmaceuticals-15-00984-s001.zip › pharmaceuticals-1832585-supplementary.pdf]

Supporting Information

Total Synthesis of the Antimycobacterial Natural Product Chlorflavonin and Analogs via a Late-stage Ruthenium(II)-Catalyzed *ortho*-C(sp^2)-H-Hydroxylation

Alexander Berger[a], Talea Knak[a], Anna-Lene Kiffe-Delf[b], Korana Mudrovcic[a], Vinayak Singh[c], Mathew Njoroge[d], Bjoern B. Burckhardt[e], Mohanraj Gopalswamy[a], Beate Lungerich[a], Lutz Ackermann[f], Holger Gohlke[a,g], Kelly Chibale[c], Rainer Kalscheuer[b], and Thomas Kurz[a]*

[a] Institute of Pharmaceutical and Medicinal Chemistry, Heinrich Heine University Düsseldorf, Universitätsstraße 1, 40225 Düsseldorf, Germany

[b] Institute of Pharmaceutical Biology and Biotechnology, Heinrich Heine University Düsseldorf, Universitätsstraße 1, 40225 Düsseldorf, Germany

[c] Drug Discovery and Development Centre (H3D), University of Cape Town; South African Medical Research Council Drug Discovery and Development Research Unit, Department of Chemistry and Institute of Infectious Disease and Molecular Medicine, University of Cape Town, Rondebosch 7701, South Africa.

[d] Drug Discovery and Development Centre (H3D), University of Cape Town, Rondebosch 7701, South Africa.

[e] Institute of Clinical Pharmacy and Pharmacotherapy, Heinrich Heine University Düsseldorf, Universitätsstraße 1, 40225 Düsseldorf, Germany

[f] Institut für Organische und Biomolekulare Chemie, Georg-August-Universität Göttingen, Tammannstraße 2, 37077 Göttingen, Germany

[g] John-von-Neumann-Institute for Computing (NIC), Jülich Supercomputing Centre (JSC), Institute of Biological Information Processing (IBI-7: Structural Biochemistry), and Institute of Bio- and Geosciences (IBG-4: Bioinformatics), Forschungszentrum Jülich GmbH, 52428 Jülich, Germany

*E-mail: thomas.kurz@hhu.de

Table of contents

1. General information ... 3
2. Experimental procedures ... 4
   2.1. Intermediates synthesized for synthetic approach A ... 4
   2.2. Intermediates synthesized for synthetic approach B ... 5
   2.3. Reaction optimization ... 7
   2.4. Experimental procedures for the synthesis of chlorflavonin analogs ... 11
      2.4.1. 3-Brom-2-hydroxy-5-methyl-benzaldehyd (2j) ... 11
      2.4.2. General procedure for MOM ether protection ... 11
      2.4.3. General procedure for flavonol synthesis ... 13
      2.4.4. General procedure for methylation of flavonols ... 17
      2.4.5. General procedure for deprotection of MOM ether ... 21
      2.4.6. Synthesis of 2-(3-bromo-2-(difluoromethoxy)phenyl)-3,7,8-trimethoxy-4H-chromen-4-one (19) 25
      2.4.7. General procedure for ruthenium(II)-catalyzed *ortho*-C(sp^2)-H-hydroxylation ... 25
3. Methods ... **Error! Bookmark not defined.**
3.1. Physicochemical properties ... **Error! Bookmark not defined.**
   3.1.1. Stability ... **Error! Bookmark not defined.**
   3.1.2. Solubility ... **Error! Bookmark not defined.**
   3.1.3. Metabolic stability ... **Error! Bookmark not defined.**
   3.1.4. NMR spectroscopy for pK_a determination ... **Error! Bookmark not defined.**
3.2. Determination of Minimal Inhibitory Concentration (MIC) ... **Error! Bookmark not defined.**
3.3. Determination of Cytotoxicity ... **Error! Bookmark not defined.**
3.4. Quantitative structure-activity relationship model ... **Error! Bookmark not defined.**
   3.4.1. Relative free energy calculations with FEW workflow ... **Error! Bookmark not defined.**
   3.4.2. Solubility and partition coefficient predictions ... **Error! Bookmark not defined.**
4. Results of relative free energy calculations ... 32
5. Spectral Copies of ^{1}H-, ^{13}C- and ^{19}F-NMR Data ... 38
6. References ... 56

1. General information

Reagents: Commercially available reagents and solvents were purchased from Apollo Scientific, Sigma-Aldrich, TCI, BLDpharm, Carbolution, ABCR GmbH, Acros Organics or Alfa Aesar and were used without further purification. Dry solvents were purchased from Acros Organics.

Chromatography: Analytical thin-layer chromatography was performed using silica gel 60 F254 aluminium plates. Compound spots were visualized either by UV light (254 nm) or by staining with a solution of 1% $FeCl_3$ in ethanol. Flash chromatography was performed on CombiFlash® Rf 200 using RediSep™ Rf-columns.

NMR Spectroscopy: ^{1}H-, ^{13}C-, and ^{19}F-NMR spectra were recorded with Bruker Avance III – 300, Bruker Avance III – 600 or Bruker Avance DRX – 500 spectrometers. ^{1}H- and ^{13}C-NMR signals were calibrated to the residual proton and carbon resonance of the solvent: $CDCl_3$ (^{1}H-NMR δ = 7.26 ppm, ^{13}C-NMR δ = 77.2 ppm), DMSO (^{1}H-NMR δ = 2.50 ppm, ^{13}C-NMR δ = 39.5 ppm). The following abbreviations were used to describe peak splitting patterns when appropriate: s = singlet, d = doublet, t = triplet, q = quartet, h = hextet, m = multiplet, dd = doublet of doublet, td = doublet of triplet, ddd = doublet of doublet of doublet and brs = broad singlet. Coupling constants, J, were reported in Hertz unit (Hz).

High Resolution Mass Spectrometry: ESI-MS data were recorded with UHR-QTOF maXis 4G.

Melting Points: Melting points were measured on a Büchi M 565 instrument and are not corrected.

HPLC: Reverse-phase high performance liquid chromatography data were measured with Varian ProStar 210 with a Phenomenex Luna C-18 (2) particle size 5 µm (250 x 4.6 mm) column. The detection took place with the UV detector Varian ProStar 330 at 220-254 nm and eluents water/acetonitrile with 0.1% TFA were used.

Table S1. Literature for known compounds.

Compound	Structure	Literature
	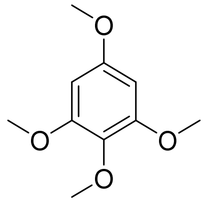	Y. Bai, X. He, Y. Bai, Y. Sun, Z. Zhao, X. Chen, B. Li, J. Xie, Y. Li, P. Jia, X. Meng, Y. Zhao, Y. Ding, C. Xiao, S. Wang, J. Yu, S. Liao, Y. Zhang, Z. Zhu, Q. Zhang, Y. Zhao, F. Qin, Y. Zhang, X. Wei, M. Zeng, J. Liang, Y. Cuan, G. Shan, T. P. Fan, B. Wu, X. Zheng, *Eur. J. Med. Chem.* **2019**, *183*, 111650.
**8**	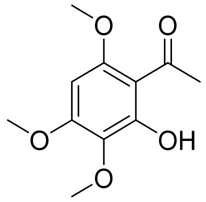	M. Tsukayama, Y. Kawamura, T. Ishizuka, S. Hayashi, F. Torii, *Heterocycles* **2003**, *60*, 2775–2784.
**9**	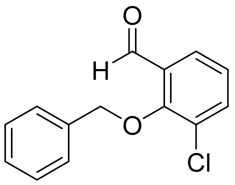	R. Bognár, A. L. Tökés, *Acta Chim. Acad. Sci. Hung* **1981**, *107*, 365–368.

**2d**	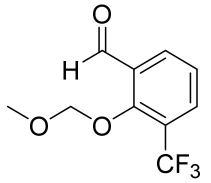	A. M. Daly, D. G. Gilheany, *Tetrahedron Asymmetry* **2003**, *14*, 127–137.

2. Experimental procedures

2.1. Intermediates synthesized for synthetic approach A

2-Methoxy-1-(2,3,4,6-tetramethoxyphenyl)ethan-1-one (6)

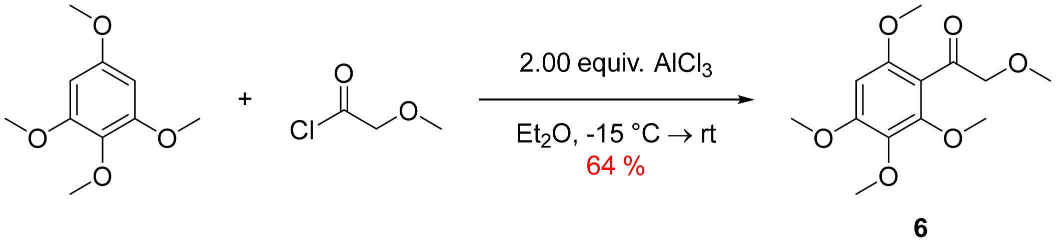

6

A solution of 1,2,3,5-tetramethoxybenzene (1.00 g, 5.04 mmol, 1.00 equiv.) and $AlCl_3$ (1.37 g, 10.1 mmol, 2.00 equiv.) in dry diethyl ether 30.0 mL was cooled to -20 °C under argon atmosphere. 2-Methoxyacetyl chloride (0.66 g, 6.05 mmol, 1.20 equiv.) was added dropwise and the reaction was stirred at ambient temperature for 24 h. The reaction mixture was quenched with the addition of 50 g ice and 1 N hydrochloric acid 50 mL. The aqueous phase was extracted four times with diethyl ether. The combined organic phase was washed with saturated NaCl solution, dried over Na_2SO_4, filtered, concentrated, and the remaining residue purified by flash chromatography (eluent: hexane/EtOAc = 9/1) to afford compound 6 as a brown solid (0.87 g, 64%); $\boldsymbol{R_f}$ 0.21 (hexane/EtOAc = 7/3); **m.p.** 90.1 °C; **^{1}H-NMR (300 MHz, $CDCl_3$)** δ 6.22 (s, 1H), 4.31 (d, *J* = 2.3 Hz, 2H), 3.85 (d, *J* = *1*.3 Hz, 6H), 3.75 (d, *J* = 3.3 Hz, 6H), 3.42 (s, 3H) ppm; **^{13}C-NMR (75 MHz, $CDCl_3$)** δ 200.6, 155.7, 153.4, 151.9, 136.5, 115.5, 92.7, 78.9, 62.2, 61.2, 59.4, 56.4 ppm.

1-(2-Hydroxy-3,4,6-trimethoxyphenyl)-2-methoxyethan-1-one (7)

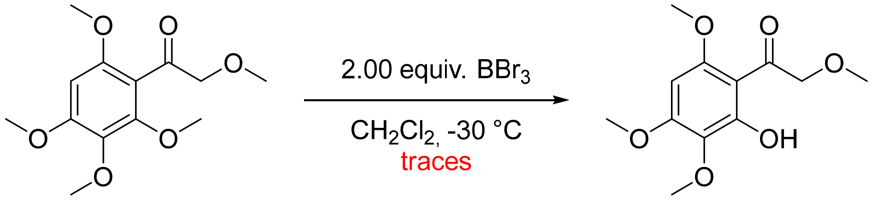

6 **7**

To a solution of 2-methoxy-1-(2,3,4,6-tetramethoxyphenyl)ethan-1-one (50 mg, 168 µmmol, 1.00 equiv.) in dry dichloromethane 5.0 mL was added BBr_3 (1 M in CH_2CL_2; 161 µL, 168 µmol, 1.00 equiv.) at -30 °C. After 30 min and 60 min additional BBr_3 (1 M in CH_2CL_2; 161 µL, 168 µmol, 1.00 equiv.) were added and the reaction was stirred at -30 °C for 4 h. The reaction was quenched by the addition of 6 N hydrochloric acid 5.0 mL and extracted thrice with dichloromethane. The combined organic phase was washed twice with 2 M LiOH 5.0 mL, the aqueous layer acidified with 6 N hydrochloric acid and extracted thrice with dichloromethane. The combined organic phase was washed with saturated NaCl solution, dried over Na_2SO_4, filtered, and concentrated to afford compound 7 as a light-yellow solid in traces; **^{1}H-NMR (300 MHz, $CDCl_3$)** δ 13.49 (s, 1H), 5.96 (s, 1H), 4.59 (s, 2H), 3.94 (s, 3H), 3.90 (s, 3H), 3.81 (s, 3H), 3.50 (s, 3H) ppm.

2.2.Intermediates synthesized for synthetic approach B

3-(2-(Benzyloxy)-3-chlorophenyl)-1-(2-hydroxy-3,4,6-trimethoxyphenyl)prop-2-en-1-one (10a)

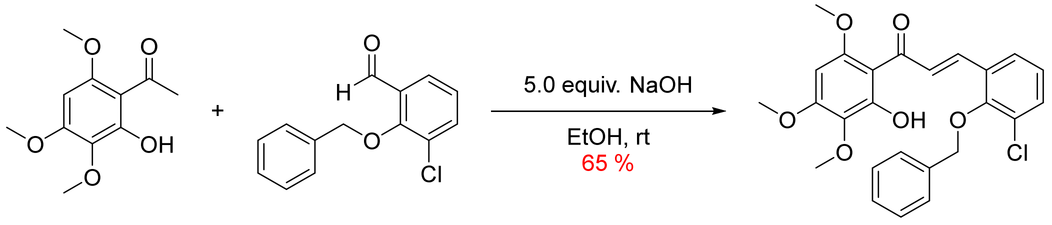

8 **9** **10a**

To a solution of 1-(2-hydroxy-3,4,6-trimethoxyphenyl)ethan-1-one (1.47 g, 6.49 mmol, 1.00 equiv.) and 2-(benzyloxy)-3-chlorobenzaldehyde (1.60 g, 6.49 mmol, 1.00 equiv.) in ethanol (25.0 mL) was added 5 N NaOH solution (6.49 mL, 32.4 mmol, 5.00 equiv.) and stirred at ambient temperature for 16 h. The solvent was removed under reduced pressure. The residue was treated with water 100 mL and at 0 °C the pH of the suspension was adjusted to 7 with 2 N hydrochloric acid solution. The aqueous phase was extracted four times with dichloromethane. The combined organic phase was washed with a saturated NaCl solution (50 mL), dried over Na_2SO_4, filtered, concentrated, and purified by flash chromatography (eluent: hexane/EtOAc + 0.1% TEA = 6/4) to afford compound 10a as an orange solid (1.91 g, 65%); $\boldsymbol{R_f}$ 0.35 (hexane/EtOAc = 1/1); **m.p.** 98.7 °C; **^{1}H-NMR (300 MHz, CDCl$_3$)** δ 13.88 (br s, 1H), 8.00 (d, *J* = *1*5.8 Hz, 1H), 7.90 (d, *J* = *1*5.7 Hz, 1H), 7.49 – 7.58 (m, 3H), 7.44 (dd, *J* = 7.9, 1.6 Hz, 1H), 7.29 – 7.42 (m, 3H), 7.12 (td, *J* = 7.9, 0.5 Hz, 1H), 5.96 (s, 1H), 4.99 (s, 2H), 3.95 (s, 3H), 3.85 (s, 3H), 3.79 (s, 3H) ppm; **^{13}C-NMR (75 MHz, CDCl$_3$)** δ 193.2, 159.5, 158.7, 158.7, 154.2, 137.0, 136.4, 131.9, 131.8, 131.0, 129.9, 129.4, 129.0, 128.7, 128.6, 127.1, 125.3, 107.0, 87.1, 76.2, 61.0, 56.2, 56.0 ppm.

2-(2-(benzyloxy)-3-chlorophenyl)-5,7,8-trimethoxy-4*H*-chromen-4-one (10)

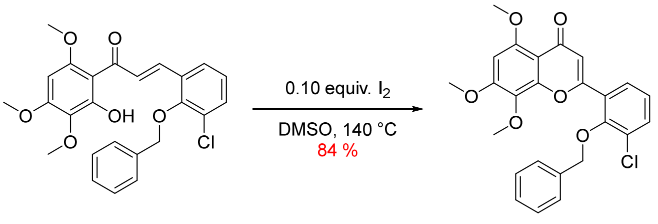

10a **10**

A solution of 3-(2-(benzyloxy)-3-chlorophenyl)-1-(2-hydroxy-3,4,6-trimethoxyphenyl)prop-2-en-1-one (4.82 g, 10.6 mmol, 1.00 equiv.) and iodine (0.27 g, 1.06 mmol, 0.10 equiv.) in dimethyl sulfoxide 40 mL was degassed with nitrogen for 10 min. Under nitrogen atmosphere the reaction mixture was stirred at 140 °C for 2 h 30 min. After cooling to ambient temperature, a solution of sodium thiosulfate 50 mL and EtOAc 50 mL were added. The organic phase was washed four times with a saturated NaCl solution (50 mL), dried over Na_2SO_4, filtered, concentrated, and the remaining residue purified by recrystallization from methanol to afford compound 10 as yellow crystals (4.05 g, 84%); $\boldsymbol{R_f}$ 0.11 (hexane/EtOAc = 1/3); **m.p.** 170.2 °C; **^{1}H-NMR (300 MHz, CDCl$_3$)** δ 7.67 (dd, *J* = 7.8, 1.7 Hz, 1H), 7.57 (dd, *J* = 8.0, 1.6 Hz, 1H), 7.33 – 7.40 (m, 2H), 7.19 – 7.33 (m, 4H), 6.81 (s, 1H), 6.45 (s, 1H), 5.00 (s, 2H), 4.00 (s, 3H), 3.99 (s, 3H), 3.84 (s, 3H) ppm; **^{13}C-NMR (75 MHz, CDCl$_3$)** δ 177.7, 158.4, 156.8, 156.5, 153.2, 152.3, 135.8, 133.1, 130.8, 129.9, 129.2, 128.8, 128.7, 128.6, 128.1, 125.4, 113.8, 109.1, 92.8, 75.8, 61.8, 56.8, 56.5 ppm; **MS (ESI)** *m/z* $[M+H]^+$ calculated for $C_{25}H_{21}ClNaO_6$: 475.1, observed: 475.1.

2-(2-(Benzyloxy)-3-chlorophenyl)-3-hydroxy-5,7,8-trimethoxy-4*H*-chromen-4-one (11)

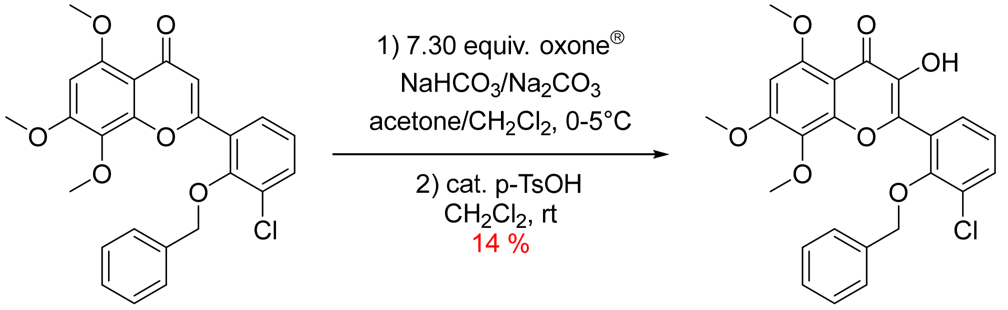

10 **11**

2-(2-(Benzyloxy)-3-chlorophenyl)-5,7,8-trimethoxy-4*H*-chromen-4-one (100 mg, 221 µmol, 1.00 equiv.) was dissolved in acetone 10.0 mL and dichloromethane 5.00 mL. At 0-5 °C a sodium bicarbonate-sodium bicarbonate buffer system 10.0 mL with the pH of 7 and oxone® (990 mg, 1.61 mmol, 7.30 equiv.) were added. The mixture was stirred at ambient temperature for 16 h. Thereafter the mixture was extracted three times with dichloromethane 20 mL and the combined organic phase concentrated under reduced pressure. The residue was dissolved in dichloromethane 10 mL and catalytic *p*-toluenesulfonic acid (5.00 mg) was added. The reaction was stirred at ambient temperature for 1 h. The solvent was removed under reduced pressure and the remaining residue purified by flash chromatography (eluent: hexane/EtOAc = 3/7) to afford compound 11 as an colourless oil (32 mg, 14%);**^{1}H-NMR (300 MHz, $CDCl_3$)** δ 7.42 – 7.51 (m, 2H), 7.03 – 7.25 (m, 7H), 6.67 (s, 1H), 6.37 (s, 1H), 4.96 (s, 2H), 3.95 (s, 3H), 3.94 (s, 3H), 3.69 (s, 3H) ppm; **^{13}C-NMR (75 MHz, $CDCl_3$)** δ 172.3, 156.8, 156.3, 153.5, 151.8, 141.8, 138.5, 136.5, 132.7, 130.0, 129.3, 128.9, 128.7, 128.5, 128.3, 128.3, 125.0, 106.8, 92.2, 76.1, 61.7, 56.7, 56.7 ppm.

2.3.Reaction optimization

Table S2. Reaction optimization.

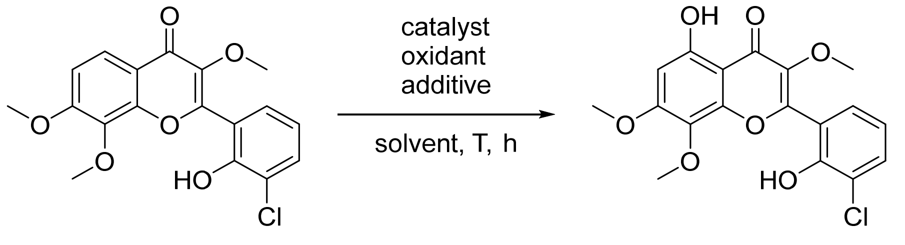

Entry	Cat. [mol%]	Oxidant [equiv.]	Additive [equiv.]	Additive [equiv.]	solvent	T [°C]	t [h]	conversion [%]
1	10.0 $Pd(OAc)_2$	1.00 $PhI(TFA)_2$	-	-	DCE	80	4	trace
2	5.00 $Pd(TFA)_2$	1.20 $PhI(TFA)_2$	-	-	DCE	80	8	trace
3	5.00 $Pd(TFA)_2$	2.00 $PhI(TFA)_2$	-	-	DCE	80	16	decomp.
4	5.00 $Pd(TFA)_2$	1.20 $PhI(TFA)_2$	-	-	9:1 TFA/TFAA	80	8	48
5	5.00 $Pd(TFA)_2$	1.50 $PhI(TFA)_2$	-	-	1:66 TFA/TFAA[1]	80	16	decomp.
6	5.00 $Pd(TFA)_2$	2.00 KPS	-	-	TFA	80	16	-
7	5.00 $Pd(OAc)_2$	2.00 *selectfluor*™	-	-	9:1 TFA/TFAA	80	16	15
8	5.00 $Pd(TFA)_2$	1.50 *selectfluor*™	-	-	1:66 TFA/TFAA[1]	80	16	43
9	7.50 $Pd(TFA)_2$	2.00 *selectfluor*™	-	-	1:66 TFA/TFAA[1]	90	20	decomp.
10	2.50 $[RuCl_2($*p*-cymene$)]_2$	1.50 *selectfluor*™	1.50 Ag_2CO_3	3.00 TFA/5.00 TFAA	DCE	80	16	-
11	5.00 $[RuCl_2($*p*-cymene$)]_2$	1.50 *selectfluor*™	1.50 Ag_2CO_3	3.00 TFA/5.00 TFAA	DCE	80	16	trace

12	5.00 $[RuCl_2(p\text{-cymene})]_2$	1.50 *selectfluor*™	0.20 Ag_2CO_3	3.00 TFA/5.00 TFAA	DCE	80	16	trace[2]
13	5.00 $[RuCl_2(p\text{-cymene})]_2$	1.20 oxone®	1.50 Ag_2CO_3	-	1:66 TFA/TFAA[1]	80	8	11
14	5.00 $[RuCl_2(p\text{-cymene})]_2$	1.20 KPS	1.50 Ag_2CO_3	-	1:66 TFA/TFAA[1]	80	8	trace
15	5.00 $[RuCl_2(p\text{-cymene})]_2$	1.00 $PhI(TFA)_2$	1.00 Ag_2CO_3	-	1:66 TFA/TFAA[1]	80	16	32
16	5.00 $[RuCl_2(p\text{-cymene})]_2$	1.20 $PhI(TFA)_2$	0.20 Ag_2CO_3	-	1:66 TFA/TFAA[1]	80	16	44[2]
17	5.00 $[RuCl_2(p\text{-cymene})]_2$	1.50 $PhI(TFA)_2$	1.50 Ag_2CO_3	-	1:66 TFA/TFAA[1]	80	24	43
18	5.00 $[RuCl_2(p\text{-cymene})]_2$	1.50 *selectfluor*™	1.50 Ag_2CO_3	-	1:66 TFA/TFAA[1]	80	16	51[3]
19	5.00 $[RuCl_2(p\text{-cymene})]_2$	1.10 *selectfluor*™	2.00 Ag_2CO_3	-	1:66 TFA/TFAA[1]	80	16	52[4]
20	5.00 $[RuCl_2(p\text{-cymene})]_2$	1.20 *selectfluor*™	1.20 Ag_2CO_3	-	1:66 TFA/TFAA[1]	80	6	36[4]
21	5.00 $[RuCl_2(p\text{-cymene})]_2$	1.50 *selectfluor*™	1.50 Ag_2CO_3	-	1:66 TFA/TFAA[1]	80	16	53
22	5.00 $[RuCl_2(p\text{-cymene})]_2$	1.50 *selectfluor*™	1.50 Ag_2CO_3	-	1:66 TFA/TFAA[1]	60	16	-
23	5.00 $[RuCl_2(\text{p-cymene})]_2$	1.10 *selectfluor*™	2.00 Ag_2CO_3	-	1:66 TFA/TFAA[1]	80	24	72
24	5.00 $[RuCl_2(p\text{-cymene})]_2$	1.10 *selectfluor*™	2.00 Ag_2CO_3	-	1:66 TFA/TFAA[1]	100	16	52
25	5.00 $[RuCl_2(p\text{-cymene})]_2$	1.20 *Selectfluor*™	1.20 Ag_2CO_3	-	1:66 TFA/TFAA[1]	120	2	74
26	5.00 $[RuCl_2(p\text{-cymene})]_2$	1.20 *selectfluor*™	1.20 Ag_2CO_3	-	1:66 TFA/TFAA[1]	80	16	36[3]
27	5.00 $[RuCl_2(p\text{-cymene})]_2$	1.50 *selectfluor*™	1.50 Ag_2CO_3	-	1:66 TFA/TFAA[1]	80	16	53
28	5.00 $[RuCl_2(p\text{-cymene})]_2$	1.80 *selectfluor*™	1.80 Ag_2CO_3	-	1:66 TFA/TFAA[1]	80	16	41

29	5.00 $[RuCl_2$(*p*-cymene)$]_2$	2.00 *selectfluor*™	2.00 Ag_2CO_3	-	1:66 TFA/TFAA[1]	80	16	61
30	5.00 $[RuCl_2$(*p*-cymene)$]_2$	1.10 *selectfluor*™	1.00 Ag_2CO_3	-	1:66 TFA/TFAA[1]	80	16	54
31	10.0 $[RuCl_2$(*p*-cymene)$]_2$	1.1 *selectfluor*™	1.00 Ag_2CO_3	-	1:66 TFA/TFAA[1]	80	16	60
32	5.00 $[RuCl_2$(*p*-cymene)$]_2$	1.10 *selectfluor*™	2.00 Ag_2CO_3	-	1:1.8 TFA/TFAA[1]	80	16	20
33	5.00 $[RuCl_2$(*p*-cymene)$]_2$	1.10 *selectfluor*™	2.00 Ag_2CO_3	-	1:66 TFA/TFAA[1]	80	16	48
34	5.00 $[RuCl_2$(*p*-cymene)$]_2$	1.10 *selectfluor*™	2.00 Ag_2CO_3	-	1:132 TFA/TFAA[1]	80	16	50
35	2.50 $[Ru(MesCO_2)_2$(*p*-cymene)]	1.20 APS	-	-	3:1 TFA/TFAA	80	16	-
36	2.50 $[Ru(MesCO_2)_2$(*p*-cymene)]	1.20 $PhI(TFA)_2$	-	-	DCE	80	4	-
37	2.50 $[Ru(MesCO_2)_2$(*p*-cymene)]	1.20 $PhI(OAc)_2$	-	-	DCE	80	4	-
38	2.50 $[Ru(MesCO_2)_2$(*p*-cymene)]	1.20 $PhI(OAc)_2$	-	-	3:2 TFA/TFAA	80	4	decomp.
39	2.50 $[Ru(MesCO_2)_2$(*p*-cymene)]	1.20 $PhI(OAc)_2$	-	-	2:1 TFA/TFAA	80	4	decomp.
40	5.00 $[Ru(MesCO_2)_2$(*p*-cymene)]	1.20 *selectfluor*™	-	-	1:66 TFA/TFAA[1]	80	6	54[4]
41	5.00 $[Ru(MesCO_2)_2$(*p*-cymene)]	1.50 *selectfluor*™	-	-	1:66 TFA/TFAA[1]	80	3	54
42	7.50 $[Ru(MesCO_2)_2$(*p*-cymene)]	1.50 *selectfluor*™	-	-	1:66 TFA/TFAA[1]	80	4	56

[1] 3.00 equiv. TFA; [2] pre-activation of catalyst; [3] Schlenk-technique (argon); [4] continuous addition of the oxidant

DCE: Dichloroethane; TFA: Trifluoroacetic acid; TFAA: Trifluoroacetic anhydride; KPS: Potassium persulfate; APS: ammonium persulfate, decomp. = decomposition

2.4. Experimental procedures for the synthesis of chlorflavonin analogs

2.4.1. 3-Brom-2-hydroxy-5-methyl-benzaldehyd (2j)

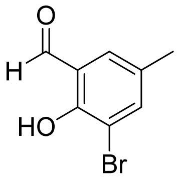

A solution of the 2-hydroxy-5-methylbenzaldehyd (5.04 g, 37.0 mmol, 1.00 equiv.) in 50 mL acetic acid was cooled to 0 °C. Bromine (2.09 mL, 40.7 mmol, 1.10 equiv) was added dropwise and the reaction mixture was stirred for 1 h at 0 °C. 50 mL ice water was added, and the formed precipitate was filtered and washed with 100 mL cold water. The obtained solid was dried under vacuum. Compound 2j as a yellow solid (6.88 g, 87%); R_f 0.64 (hexane/EtOAc = 95/5); **m.p.** 65.7 °C; **^{1}H-NMR (300 MHz, DMSO-d_6)** δ 11.01 (s, 1H), 10.03 (s, 1H), 7.75 (dd, J = 2.2, 0.7 Hz, 1H), 7.57 (dd, J = 2.3, 0.8 Hz, 1H), 2.29 (t, J = 0.7 Hz, 3H) ppm; **^{13}C-NMR (75 MHz, DMSO-d_6)** δ 195.01, 154.49, 139.82, 131.67, 130.61, 122.50, 110.70, 19.40 ppm.

2.4.2. General procedure for MOM ether protection

A solution of the respective phenol (1.00 equiv.) in dry dichloromethane (1.5 mL/mmol) was cooled to 0 °C and *N,N*-diisopropylethylamine (2.00 equiv.) was added. After 10 minutes chloromethyl methyl ether (1.20 equiv.) was added dropwise. The reaction was allowed to warm up to ambient temperature and was stirred overnight. The reaction was quenched with a saturated solution of NH_4Cl (2 mL/mmol) and extracted three times with dichloromethane (2 mL/mmol). The combined organic phase was washed with saturated NaCl solution, dried over Na_2SO_4, filtered, concentrated, and purified by flash chromatography to afford the desired product.

3-Chloro-2-(methoxymethoxy)benzaldehyde (12)[1]

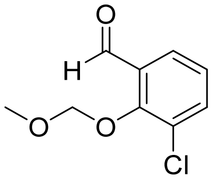

The reaction was performed according to the general procedure 2.4.2. to afford compound 12 as a white solid (12.1 g, 97%, eluent: hexane/EtOAc = 95/5); $\boldsymbol{R_f}$ 0.19 (hexane/EtOAc = 95/5); **m.p.** 43.2 °C; **^{1}H-NMR (300 MHz, CDCl$_3$)** δ 10.39 (d, J = 0.8 Hz, 1H), 7.80 (dd, J = 7.8, 1.7 Hz, 1H), 7.68 (dd, J = 7.9, 1.7 Hz, 1H), 7.23 (td, J = 7.8, 0.8 Hz, 1H), 5.24 (s, 2H), 3.64 (s, 3H) ppm; **^{13}C-NMR (75 MHz, CDCl$_3$)** δ 189.6, 156.2, 136.1, 131.6, 128.6, 126.9, 125.5, 100.7, 58.3 ppm.

3-Bromo-2-(methoxymethoxy)benzaldehyde (12a)[2]

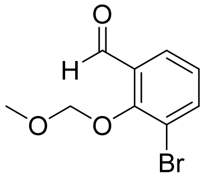

The reaction was performed according to the general procedure 2.4.2. to afford compound 12a as a white solid (5.89 g, 97%, eluent: hexane/EtOAc = 95/5); $\boldsymbol{R_f}$ 0.35 (hexane/EtOAc = 9/1); **m.p.** 54.2 °C; **^{1}H-NMR (300 MHz, CDCl$_3$)** δ 10.36 (d, J = 0.8 Hz, 1H), 7.85 (q, J = *1*.7 Hz, 1H), 7.82 (q, J = *1*.7 Hz, 1H), 7.17 (td, J = 7.8, 0.9 Hz, 1H), 5.22 (s, 2H), 3.64 (s, 3H) ppm; **^{13}C-NMR (75 MHz, CDCl$_3$)** δ 189.7, 157.3, 139.2, 131.7, 127.7, 126.0, 118.2, 101.0, 58.3 ppm.

3-Fluoro-2-(methoxymethoxy)benzaldehyde (12b)[3]

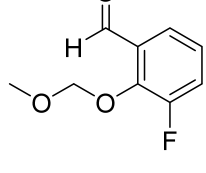

The reaction was performed according to the general procedure 2.4.2. to afford compound 12b as a colourless liquid (5.72 g, 87%, eluent: hexane/EtOAc = 95/5); $\boldsymbol{R_f}$ 0.19 (hexane/EtOAc = 95/5); **^{1}H-NMR (300 MHz, CDCl$_3$)** δ 10.42 (d, J = 0.8 Hz, 1H), 7.62 (ddd, J = 7.8, 1.7, 1.1 Hz, 1H), 7.34 (ddd, J = *1*1.2, 8.1, 1.7 Hz, 1H), 7.14 (tdd, J = 8.0, 4.5, 0.8 Hz, 1H), 5.27 (d, J = *1*.0 Hz, 2H), 3.57 (s, 3H) ppm; **^{13}C-NMR (75 MHz, CDCl$_3$)** δ 189.1 (d, $^4J(^{13}C,^{19}F)$ = 3.3 Hz), 155.3 (d, $^1J(^{13}C,^{19}F)$ = 248.9 Hz), 147.3 (d, $^2J(^{13}C,^{19}F)$ =

11.4 Hz), 131.0 (d, $^3J(^{13}C,^{19}F)$ = 1.7 Hz), 124.4 (d, $^3J(^{13}C,^{19}F)$ = 7.4 Hz), 123.9 (d, $^4J(^{13}C,^{19}F)$ = 3.4 Hz), 122.8 (d, $^2J(^{13}C,^{19}F)$ = 19.7 Hz), 99.9 (d, $^4J(^{13}C,^{19}F)$ = 7.5 Hz), 58.0 ppm; **^{19}F-NMR (282 MHz, $CDCl_3$)** δ -129.2 ppm; **HRMS (ESI)** *m/z* $[M+H]^+$ calculated for $C_9H_{10}FO_3$: 185.0608, observed: 185.0608.

3-Iodo-2-(methoxymethoxy)benzaldehyde (12c)[4]

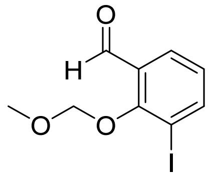

The reaction was performed according to the general procedure 2.4.2. to afford compound 12c as a colourless liquid (1.11 g, 98%, eluent: hexane/EtOAc = 95/5); ***R_f*** 0.47 (hexane/EtOAc = 9/1); **^{1}H-NMR (600 MHz, $CDCl_3$)** δ 10.31 (d, *J* = 0.9 Hz, 1H), 8.08 (dt, *J* = 7.8, 1.5 Hz, 1H), 7.86 (dt, *J* = 7.6, 1.5 Hz, 1H), 7.05 (tt, *J* = 7.8, 1.0 Hz, 1H), 5.19 (d, *J* = 1.1 Hz, 2H), 3.65 (d, *J* = 1.2 Hz, 3H) ppm; **^{13}C-NMR (75 MHz, $CDCl_3$)** δ 190.2, 160.1, 145.5, 131.3, 128.9, 126.9, 101.6, 93.6, 58.6 ppm.

2-(Methoxymethoxy)-3-methylbenzaldehyde (12e)[1]

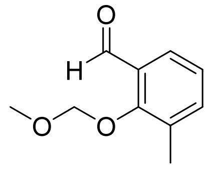

The reaction was performed according to the general procedure 2.4.2. to afford compound 12e as a colourless liquid (3.78 g, 64%, eluent: hexane/EtOAc = 95/5); ***R_f*** 0.24 (hexane/EtOAc = 9/1); **^{1}H-NMR (300 MHz, DMSO-*d*$_6$)** δ 10.23 (d, *J* = 0.8 Hz, 1H), 7.53 – 7.63 (m, 2H), 7.23 (tt, *J* = 7.5, 0.5 Hz, 1H), 5.09 (s, 2H), 3.50 (s, 3H), 2.31 (d, *J* = 0.8 Hz, 3H) ppm; **^{13}C-NMR (75 MHz, DMSO-*d*$_6$)** δ 191.1, 158.9, 137.8, 132.8, 129.8, 126.4, 125.1, 101.0, 57.8, 16.4 ppm.

3-Chloro-5-fluoro-2-(methoxymethoxy)benzaldehyde (12h)

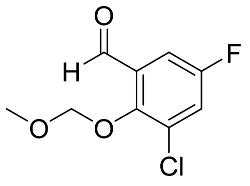

The reaction was performed according to the general procedure 2.4.2. to afford compound 12h as a white solid (4.99 g, 98%, eluent: hexane/EtOAc = 95/5); ***R_f*** 0.18 (hexane/EtOAc = 95/5); **m.p.** 72.6 °C**; ^{1}H-NMR (300 MHz, DMSO-*d*$_6$)** δ 10.16 (d, *J* = 3.0 Hz, 1H), 7.93 (dd, *J* = 8.0, 3.2 Hz, 1H), 7.50 (dd, *J* = 8.1, 3.2 Hz, 1H), 5.18 (s, 2H), 3.53 (s, 3H) ppm; **^{13}C-NMR (75 MHz, DMSO-*d*$_6$)** δ 188.6 (d, $^4J(^{13}C,^{19}F)$ = 1.7 Hz), 157.9 (d, $^1J(^{13}C,^{19}F)$ = 246.7 Hz), 152.0, 131.6 (d, $^3J(^{13}C,^{19}F)$ = 7.0 Hz), 129.3 (d, $^3J(^{13}C,^{19}F)$ = 10.3 Hz), 123.3 (d, $^2J(^{13}C,^{19}F)$ = 26.9 Hz), 112.9 (d, $^2J(^{13}C,^{19}F)$ = 23.4 Hz), 100.8 (d, $^4J(^{13}C,^{19}F)$ = 1.0 Hz), 57.8 ppm; **^{19}F-NMR (282 MHz, DMSO-*d*$_6$)** δ -114.6 ppm; **HRMS (ESI)** *m/z* $[M+H]^+$ calculated for $C_9H_9ClFO_3$: 219.0219, observed: 219.0218.

3-Bromo-5-fluoro-2-(methoxymethoxy)benzaldehyde (12i)

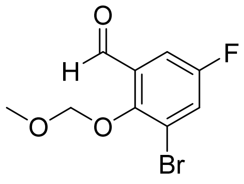

The reaction was performed according to the general procedure 2.4.2. to afford compound 12i as a white solid (2.26 g, 99%, eluent: hexane/EtOAc = 95/5); ***R_f*** 0.19 (hexane/EtOAc = 9/1); **m.p.** 73.1 °C; **^{1}H-NMR (600 MHz, DMSO-*d*$_6$)** δ 10.14 (d, *J* = 3.0 Hz, 1H), 8.04 (dd, *J* = 7.8, 3.2 Hz, 1H), 7.53 (dd, *J* = 8.1, 3.2 Hz, 1H), 5.17 (s, 2H), 3.54 (s, 3H) ppm; **^{13}C-NMR (151 MHz, DMSO-*d*$_6$)** δ 188.8, 158.2 (d, $^1J(^{13}C,^{19}F)$ = 247.6 Hz), 153.1 (d, $^4J(^{13}C,^{19}F)$ = 3.0 Hz), 131.6 (d, $^3J(^{13}C,^{19}F)$ = 6.6 Hz), 126.2 (d, $^2J(^{13}C,^{19}F)$ = 26.4 Hz), 118.9 (d, $^3J(^{13}C,^{19}F)$ = 9.4 Hz), 113.4 (d, $^2J(^{13}C,^{19}F)$ = 23.4 Hz), 101.0, 57.9 ppm; **^{19}F-NMR (565 MHz, DMSO-*d*$_6$)** δ -114.7 ppm; **HRMS (ESI)** *m/z* $[M+H]^+$ calculated for $C_9H_9BrFO_3$: 262.9714, observed: 262.9713.

3-Bromo-2-(methoxymethoxy)-5methylbenzaldehyde (12j)

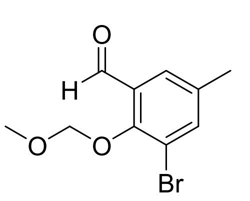

The reaction was performed according to the general procedure 2.4.2. to afford compound 12j as a white solid (7.5 g, 96%, eluent: hexane/EtOAc = 95/5); ***R_f*** 0.24 (hexane/EtOAc = 9/1); **m.p.** 47.1 °C; **^{1}H-NMR (300 MHz, DMSO-*d*$_6$)** δ 10.17 (s, 1H), 7.86 (dt, *J* = 2.3, 0.7 Hz, 1H), 7.56 (dq, *J* = 2.2, 0.7 Hz, 1H), 5.14 (s, 2H), 3.52 (s, 3H), 2.34 (t, *J* = 0.7 Hz, 3H) ppm; **^{13}C-NMR (75 MHz, DMSO-*d*$_6$)** δ: 189.79, 139.46, 136.29, 130.64, 127.82, 117.50, 100.76, 57.78, 19.78 ppm; **HRMS (ESI)** *m/z* $[M+H]^+$ calculated for $C_{10}H_{12}BrO_3$: 258.9964, observed 258.9961.

4-Chloro-2-(methoxymethoxy)benzaldehyde (12k)[5]

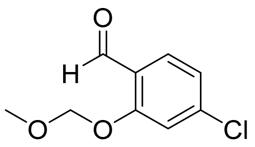

The reaction was performed according to the general procedure 2.4.2. to afford compound 12k as a white solid (3.76 g, 98%, eluent: hexane/EtOAc = 95/5); ***R_f*** 0.27 (hexane/EtOAc = 9/1); **m.p.** 58.2 °C; **^{1}H-NMR (600 MHz, CDCl$_3$)** δ 10.32 – 10.36 (m, 1H), 7.73 (dd, *J* = 8.3, 1.2 Hz, 1H), 7.40 (t, *J = 1*.5 Hz, 1H), 7.21 (ddt, *J* = 8.3, 1.8, 0.9 Hz, 1H), 5.42 (s, 2H), 3.45 (s, 3H) ppm**; ^{13}C-NMR (75 MHz, CDCl$_3$)** δ 188.3, 159.4, 140.3, 129.4, 123.8, 122.0, 115.9, 94.6, 56.3 ppm.

4-Chloro-3-(methoxymethoxy)benzaldehyde (12l)

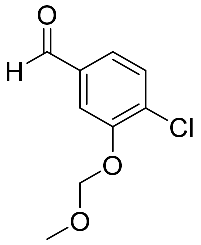

The reaction was performed according to the general procedure 2.4.2. to afford compound 12l as a white solid (3.78 g, 98%, eluent: hexane/EtOAc = 95/5); ***R_f*** 0.22 (hexane/EtOAc = 9/1); **m.p.** 29.2 °C; **^{1}H-NMR (300 MHz, DMSO-*d*$_6$)** δ 9.96 (s, 1H), 7.71 (dd, *J* = 4.9, 3.1 Hz, 2H), 7.58 (dd, *J* = 8.1, 1.8 Hz, 1H), 5.39 (s, 2H), 3.43 (s, 3H) ppm; **^{13}C-NMR (75 MHz, DMSO-*d*$_6$)** δ 192.0, 152.7, 136.0, 130.9, 128.8, 124.3, 115.1, 94.6, 56.0 ppm; **HRMS (ESI)** *m/z* $[M+H]^+$ calculated for $C_9H_{10}ClO_3$: 201.0313, observed: 201.0313.

2.4.3. General procedure for flavonol synthesis

To a solution of 1-(2-hydroxy-3,4-dimethoxyphenyl)ethan-1-one (1.00 equiv.) and the corresponding aldehyde (12a-I, 1.00 equiv.) in ethanol (2 mL/mmol) was added an aqueous solution of 5 N NaOH (5.0 equiv.). The solution was stirred at 50 °C for 24 h. After cooling to ambient temperature, the dispersion was cooled to 0 °C and slowly hydrogen peroxide 30% (w/w) in water (2.25 equiv.) was added. The dispersion was diluted with ethanol (3 mL/mmol) to allow further stirring, warmed up to ambient temperature and stirred overnight. The solvent was removed under reduced pressure. The residue was dissolved in water and at 0 °C and the pH of the suspension was adjusted to 7 with 2 N hydrochloric acid solution. The aqueous phase was extracted four times with dichloromethane (5 mL/mmol). The combined organic phase was washed with saturated NaCl solution (2 mL/mmol), dried over Na_2SO_4, filtered, concentrated, and purified by flash chromatography.

2-(3-Chloro-2-(methoxymethoxy)phenyl)-3-hydroxy-7,8-dimethoxy-4*H*-chromen-4-one (15)

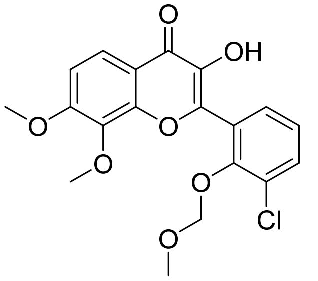

The reaction was performed according to the general procedure 2.4.3. to afford compound 15 as a yellow solid (6.83 g, 42%, eluent: hexane/EtOAc = 7/3); $\boldsymbol{R_f}$ 0.28 (hexane/EtOAc = 1/1); **m.p.** 166.7 °C; **^{1}H-NMR (300 MHz, DMSO-*d*$_6$)** δ 9.16 (s, 1H), 7.89 (d, *J* = 9.1 Hz, 1H), 7.70 (dd, *J* = 8.0, 1.6 Hz, 1H), 7.59 (dd, *J* = 7.7, 1.6 Hz, 1H), 7.35 (d, *J* = 7.9 Hz, 1H), 7.30 (d, *J* = 9.1 Hz, 1H), 4.98 (s, 2H), 3.97 (s, 3H), 3.83 (s, 3H), 3.14 (s, 3H) ppm; **^{13}C-NMR (75 MHz, DMSO-*d*$_6$)** δ 172.4, 155.9, 151.0, 149.3, 144.9, 138.7, 136.0, 132.1, 130.3, 127.4, 125.3, 120.3, 116.8, 110.7, 99.0, 60.8, 56.6, 56.5 ppm; **HRMS (ESI)** *m/z* $[M+H]^+$ calculated for $C_{19}H_{18}ClO_7$: 393.0736, observed: 393.0740.

2-(3-Bromo-2-(methoxymethoxy)phenyl)-3-hydroxy-7,8-dimethoxy-4*H*-chromen-4-one (15a)

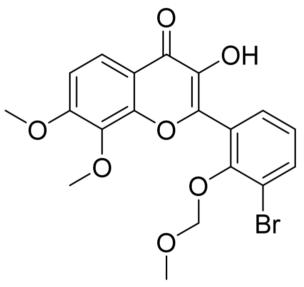

The reaction was performed according to the general procedure 2.4.3. to afford compound 15a as a yellow solid (4.04 g, 39%, eluent: hexane/EtOAc = 7/3); $\boldsymbol{R_f}$ 0.21 (hexane/EtOAc = 1/1); **m.p.** 126.3 °C; **^{1}H-NMR (300 MHz, DMSO-*d*$_6$)** δ 9.13 (s, 1H), 7.87 (d, *J* = 9.1 Hz, 1H), 7.84 (dd, *J* = 8.1, 1.6 Hz, 1H), 7.61 (dd, *J* = 7.6, 1.6 Hz, 1H), 7.29 (d, *J* = 9.2 Hz, 1H), 7.26 (t, *J* = 7.9 Hz, 1H), 4.94 (s, 2H), 3.96 (s, 3H), 3.83 (d, *J* = 0.8 Hz, 3H), 3.12 (s, 3H) ppm; **^{13}C-NMR (75 MHz, DMSO-*d*$_6$)** δ 172.4, 155.9, 152.1, 149.3, 145.1, 138.6, 136.0, 135.2, 131.0, 127.3, 125.7, 120.3, 117.3, 116.8, 110.8, 99.1, 60.9, 56.7, 56.5 ppm; **HRMS (ESI)** *m/z* $[M+H]^+$ calculated for $C_{19}H_{18}BrO_7$: 437.0230, observed: 437.0231.

2-(3-Fluoro-2-(methoxymethoxy)phenyl)-3-hydroxy-7,8-dimethoxy-4*H*-chromen-4-one (15b)

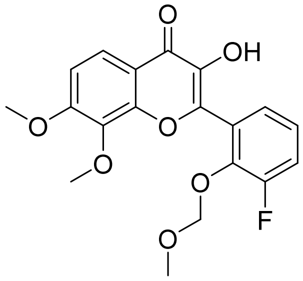

The reaction was performed according to the general procedure 2.4.3. to afford compound 15b as a yellow solid (4.98 g, 43%, eluent: hexane/EtOAc = 7/3); $\boldsymbol{R_f}$ 0.22 (hexane/EtOAc = 3/2); **m.p.** 148.2 °C; **^{1}H-NMR (300 MHz, DMSO-*d*$_6$)** δ 9.12 (s, 1H), 7.88 (d, *J* = 9.1 Hz, 1H), 7.48 (ddd, *J* = *1*1.4, 8.2, 1.6 Hz, 1H), 7.43 (dt, *J* = 7.8, 1.2 Hz, 1H), 7.26 – 7.34 (m, 2H), 5.05 (s, 2H), 3.97 (s, 3H), 3.83 (s, 3H), 3.19 (s, 3H) ppm; **^{13}C-NMR (75 MHz, DMSO-*d*$_6$)** δ 172.4, 155.9, 155.1 (d, $^{1}J(^{13}C,^{19}F)$ = 245.9 Hz), 149.3, 144.6 (d, $^{4}J(^{13}C,^{19}F)$ = 3.1 Hz), 142.4 (d, $^{2}J(^{13}C,^{19}F)$ = 12.2 Hz), 138.7, 136.0, 127.2 (d, $^{4}J(^{13}C,^{19}F)$ = 2.5 Hz), 126.8 (d, $^{3}J(^{13}C,^{19}F)$ = 3.3 Hz), 124.6 (d, $^{3}J(^{13}C,^{19}F)$ = 8.5 Hz), 120.3, 118.5 (d, $^{2}J(^{13}C,^{19}F)$ = 19.5 Hz), 116.8, 110.8, 98.8 (d, $^{4}J(^{13}C,^{19}F)$ = 5.2 Hz), 60.8, 56.5, 56.2 ppm; **^{19}F-NMR (282 MHz, DMSO-*d*$_6$)** δ -129.2 ppm; **HRMS (ESI)** *m/z* $[M+H]^+$ calculated for $C_{19}H_{18}FO_7$: 377.1031, observed: 377.1034.

3-Hydroxy-2-(3-iodo-2-(methoxymethoxy)phenyl)-7,8-dimethoxy-4*H*-chromen-4-one (15c)

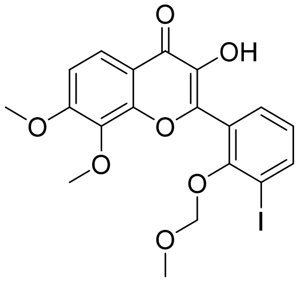

The reaction was performed according to the general procedure 2.4.3. to afford compound 15c as a yellow solid (0.67 g, 37%, eluent: hexane/EtOAc = 7/3); $\boldsymbol{R_f}$ 0.42 (hexane/EtOAc = 1/1); **m.p.** 130.2 °C; **^{1}H-NMR (300 MHz, DMSO-*d*$_6$)** δ 9.09 (s, 1H), 8.03 (dd, *J* = 7.9, 1.6 Hz, 1H), 7.87 (d, *J* = 9.1 Hz, 1H), 7.61 (dd, *J* = 7.6, 1.6 Hz, 1H), 7.29 (d, *J* = 9.2 Hz, 1H), 7.10 (t, *J* = 7.7 Hz, 1H), 4.89 (s, 2H), 3.96 (s, 3H), 3.82 (s, 3H), 3.12 (s, 3H) ppm; **^{13}C-NMR (75 MHz, DMSO-*d*$_6$)** δ 172.9, 156.4, 155.3, 149.8, 145.9, 141.8, 139.0, 136.5, 132.2, 126.6, 126.6, 120.8, 117.3, 111.2, 99.8, 94.2, 61.3, 57.4, 57.0 ppm; **HRMS (ESI)** *m/z* $[M+H]^+$ calculated for $C_{18}H_{16}ClO_7$: 485.0092, observed: 485.0094.

3-Hydroxy-7,8-dimethoxy-2-(2-(methoxymethoxy)-3-(trifluoromethyl)phenyl)-4*H*-chromen-4-one (15d)

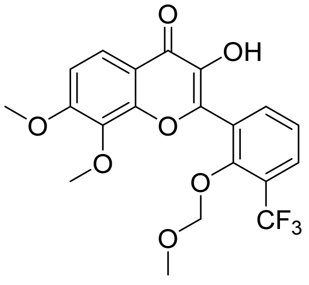

The reaction was performed according to the general procedure 2.4.3. to afford compound 15d as a yellow solid (6.37 g, 32%, eluent: hexane/EtOAc = 7/3); ***R*** R_f 0.34 (hexane/EtOAc = 1/1); **m.p.** 174.1 °C**;** ^{1}**H-NMR (300 MHz, DMSO-*d*$_6$)** δ 9.24 (br s, 1H), 7.90 (dd, *J* = 12.1, 7.8 Hz, 1H), 7.88 (d, *J* = 9.1 Hz, 1H), 7.73 (ddd, *J* = 15.5, 7.7, 1.7 Hz, 1H), 7.48 (t, *J* = 7.8 Hz, 1H), 7.30 (d, *J* = 9.1 Hz, 1H), 4.89 (s, 2H), 3.96 (s, 3H), 3.83 (s, 3H), 3.13 (s, 3H) ppm; ^{13}**C-NMR (75 MHz, DMSO-*d*$_6$)** δ 172.7, 155.8, 149.8, 144.2, 138.9, 136.1, 135.2, 120.5, 120.2, 120.1, 118.9, 117.4, 117.1, 110.8, 110.5, 99.5, 89.4, 60.9, 56.5, 56.5 ppm; ^{19}**F-NMR (565 MHz, DMSO-*d*$_6$)** δ -59.3 ppm; **HRMS (ESI)** *m/z* $[M+H]^+$ calculated for $C_{20}H_{18}F_3O_7$: 427.0999, observed: 427.0999.

3-Hydroxy-7,8-dimethoxy-2-(2-(methoxymethoxy)-3-methylphenyl)-4*H*-chromen-4-one (15e)

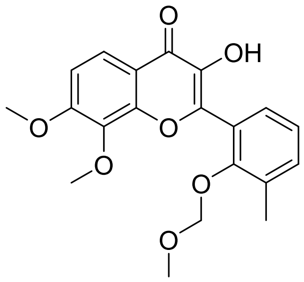

The reaction was performed according to the general procedure 2.4.3. to afford compound 15e as a yellow solid (3.57 g, 56%, eluent: hexane/EtOAc = 7/3); R_f 0.42 (hexane/EtOAc = 1/1); **m.p.** 130.3 °C**;** ^{1}**H-NMR (300 MHz, DMSO-*d*$_6$)** δ 8.91 (s, 1H), 7.88 (d, *J* = 9.1 Hz, 1H), 7.37 – 7.44 (m, 2H), 7.29 (d, *J* = 9.2 Hz, 1H), 7.20 (t, *J* = 7.6 Hz, 1H), 4.85 (s, 2H), 3.96 (s, 3H), 3.83 (s, 3H), 3.15 (s, 3H), 2.34 (s, 3H) ppm; ^{13}**C-NMR (75 MHz, DMSO-*d*$_6$)** δ 172.9, 156.2, 154.4, 149.8, 147.1, 138.9, 136.6, 133.5, 132.1, 129.5, 125.7, 124.3, 120.7, 117.3, 111.1, 99.3, 61.3, 57.0, 56.8, 16.9 ppm; **HRMS (ESI)** *m/z* $[M+H]^+$ calculated for $C_{20}H_{21}O_7$: 373.1282, observed: 373.1282.

2-(3-Bromo-2-fluorophenyl)-3-hydroxy-7,8-dimethoxy-*4H*-chromen-4-one (15f)

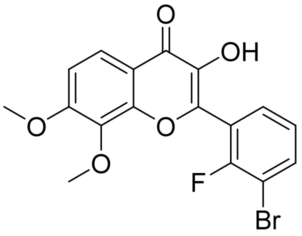

The reaction was performed according to the general procedure 2.4.3. and purified by flash column chromatography (eluent: hexane/EtOAc = 7/3). A mixture of compound 15f (approx. 80 %), the corresponding flavanone (2-(3-bromo-2-fluorophenyl)-7,8-dimethoxychroman-4-one) and 2-(3-bromo-2-ethoxyphenyl)-3-hydroxy-7,8-dimethoxy-*4H*-chromen-4-one was obtained. The crude product was used without further purification. ^{1}**H-NMR (600 MHz, DMSO-*d*$_6$)** δ 9.49 (s, 1H), 7.91 (ddd, *J* = 8.1, 6.5, 1.6 Hz, 1H), 7.88 (d, *J* = 9.0 Hz, 1H), 7.78 (ddd, *J* = 7.9, 6.3, 1.6 Hz, 1H), 7.36 (t, *J* = 7.9 Hz, 1H), 7.31 (dd, *J* = 9.2, 2.7 Hz, 1H), 3.96 (s, 3H), 3.84 (s, 2H) ppm; ^{13}**C-NMR (75 MHz, DMSO-*d*$_6$)** δ 172.41, 157.03, 156.12, 149.42, 145.15, 141.73 (d, $^{4}J(^{13}C,^{19}F)$ = 1.6 Hz), 139.13, 136.09, 135.34, 130.70 (d, $^{4}J(^{13}C,^{19}F)$ = 2.0 Hz), 125.93 (d, $^{3}J(^{13}C,^{19}F)$ = 4.2 Hz), 120.57 (d, $^{2}J(^{13}C,^{19}F)$ = 26.1 Hz), 116.76, 110.95, 109.06 (d, $^{2}J(^{13}C,^{19}F)$ = 20.9 Hz), 60.92, 56.55 ppm; ^{19}**F-NMR (282 MHz, DMSO-*d*$_6$)** δ -104.85 ppm; **HRMS (ESI)** *m/z* $[M+H]^+$ calculated for $C_{17}H_{13}BrFO_7$: 394.9925, observed: 394.9927.

2-(3-Chloro-5-fluoro-2-(methoxymethoxy)phenyl)-3-hydroxy-7,8-dimethoxy-4*H*-chromen-4-one (15h)

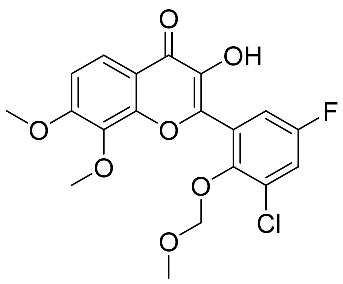

The reaction was performed according to the general procedure 2.4.3. to afford compound 15h as a yellow solid (2.11 g, 34%, eluent: hexane/EtOAc = 7/3); R_f 0.18 (hexane/EtOAc = 3/2); ^{1}**H-NMR (300 MHz, DMSO-*d*$_6$)** δ 9.29 (s, 1H), 7.87 (d, *J* = 9.0 Hz, 1H), 7.75 (dd, *J* = 8.2, 3.1 Hz, 1H), 7.48 – 7.55 (m, 1H), 7.27 – 7.33

(m, 1H), 4.94 (s, 2H), 3.96 (s, 3H), 3.83 (s, 3H), 3.11 (s, 3H) ppm; **^{13}C-NMR (75 MHz, DMSO-*d*$_6$)** δ 172.9, 157.8 (d, $^1J(^{13}C,^{19}F)$ = 244.9 Hz), 156.5, 149.8, 148.4 (d, $^4J(^{13}C,^{19}F)$ = 3.4 Hz), 144.2 (d, $^4J(^{13}C,^{19}F)$ = 1.3 Hz), 139.3, 136.5, 128.9 (d, $^3J(^{13}C,^{19}F)$ = 11.6 Hz), 128.6 (d, $^3J(^{13}C,^{19}F)$ = 9.7 Hz), 120.8, 119.7 (d, $^2J(^{13}C,^{19}F)$ = 26.2 Hz), 117.4 (d, $^2J(^{13}C,^{19}F)$ = 31.8 Hz), 117.4, 111.3, 99.7, 61.4, 57.2, 57.0 ppm; **^{19}F-NMR (565 MHz, DMSO-*d*$_6$)** δ -116.3 ppm; **HRMS (ESI)** *m/z* $[M+H]^+$ calculated for $C_{19}H_{17}ClFO_7$: 411.0641, observed: 411.0644.

2-(3-Bromo-5-fluoro-2-(methoxymethoxy)phenyl)-3-hydroxy-7,8-dimethoxy-4*H*-chromen-4-one (15i)

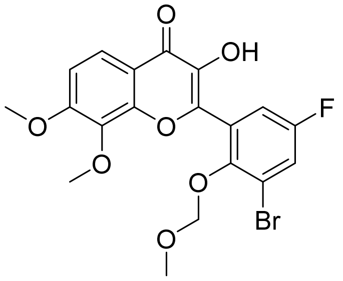

The reaction was performed according to the general procedure 2.4.3. to afford compound 15i as a yellow solid (1.26 g, 32%, eluent: hexane/EtOAc = 7/3); ***R$_f$*** 0.19 (hexane/EtOAc = 3/2); **m.p.** 230.8 °C; **^{1}H-NMR (300 MHz, CDCl$_3$)** δ 8.00 (d, *J* = 9.1 Hz, 1H), 7.49 (dd, *J* = 7.5, 3.1 Hz, 1H), 7.34 (dd, *J* = 8.1, 3.1 Hz, 1H), 7.11 (d, *J* = 9.1 Hz, 1H), 5.00 (s, 2H), 4.02 (s, 3H), 3.99 (s, 3H), 3.23 (s, 3H) ppm; **^{13}C-NMR (75 MHz, CDCl$_3$)** δ 175.3, 158.4, 156.9, 151.6, 150.5, 143.4, 138.4, 137.0, 136.7, 122.7, 121.2, 117.5, 116.2, 110.7, 110.6, 100.3, 61.9, 57.7, 56.8 ppm; **^{19}F-NMR (282 MHz, CDCl$_3$)** δ -115.9 ppm; **HRMS (ESI)** *m/z* $[M+H]^+$ calculated for $C_{19}H_{17}BrFO_7$: 455.0136, observed: 455.0138.

2-(3-Bromo-2-(methoxymethoxy)-5-methylphenyl)-3-hydroxy-7,8-dimethoxy-*4H*-chromen-4-one (15j)

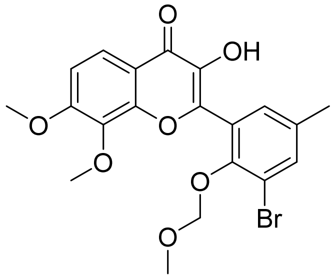

The reaction was performed according to the general procedure 2.4.3. to afford compound 15j as a yellow solid (3.86 g, 31%, eluent: hexane/EtOAc = 7/3); ***R$_f$*** 0.21 (hexane/EtOAc = 3/2); **m.p.** 182.6 °C; **^{1}H-NMR (300 MHz, DMSO-*d*$_6$)** δ 9.09 (s, 1H), 7.87 (d, *J* = 9.0 Hz, 1H), 7.68 (dd, *J* = 2.2, 0.8 Hz, 1H), 7.40 (dd, *J* = 2.2, 0.8 Hz, 1H), 7.29 (d, *J* = 9.1 Hz, 1H), 4.90 (s, 2H), 3.96 (s, 3H), 3.82 (s, 3H), 3.10 (s, 3H), 2.34 (t, *J* = 0.7 Hz, 3H) ppm; **^{13}C-NMR (75 MHz, DMSO-*d*$_6$)** δ 172.39, 155.87, 149.80, 149.29, 145.24, 138.57, 136.03, 135.37, 135.30, 131.18, 126.84, 120.28, 116.94, 116.80, 110.76, 99.06, 60.85, 56.62, 56.48, 19.77 ppm; **HRMS (ESI)** *m/z* $[M+H]^+$ calculated for $C_{20}H_{20}BrO_7$: 451.0387, observed: 451.0393.

2-(4-Chloro-2-(methoxymethoxy)phenyl)-3-hydroxy-7,8-dimethoxy-4*H*-chromen-4-one (15k)

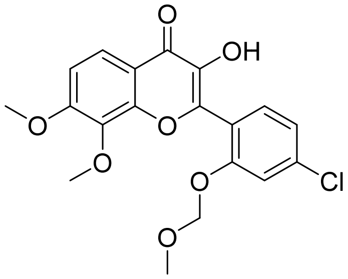

The reaction was performed according to the general procedure 2.4.3. to afford compound 15k as a yellow solid (3.62 g, 51%, eluent: hexane/EtOAc = 7/3); ***R$_f$*** 0.47 (hexane/EtOAc = 1/1); **m.p.** 193.4 °C; **^{1}H-NMR (300 MHz, DMSO-*d*$_6$)** δ 9.01 (s, 1H), 7.86 (d, *J* = 9.0 Hz, 1H), 7.56 (d, *J* = 8.2 Hz, 1H), 7.35 (d, *J* = 2.0 Hz, 1H), 7.28 (d, *J* = 9.1 Hz, 1H), 7.22 (dd, *J* = 8.2, 2.0 Hz, 1H), 5.27 (s, 2H), 3.95 (s, 3H), 3.80 (s, 3H), 3.35 (s, 3H) ppm; **^{13}C-NMR (75 MHz, DMSO-*d*$_6$)** δ 172.8, 156.2, 155.8, 149.8, 145.5, 139.0, 136.6, 135.9, 132.9, 121.7, 120.5, 120.3, 117.3, 115.9, 111.2, 94.8, 61.2, 56.9, 56.2 ppm; **HRMS (ESI)** *m/z* $[M+H]^+$ calculated for $C_{19}H_{18}ClO_7$: 393.0736, observed: 393.0741.

2-(4-Chloro-3-(methoxymethoxy)phenyl)-3-hydroxy-7,8-dimethoxy-4*H*-chromen-4-one (12l)

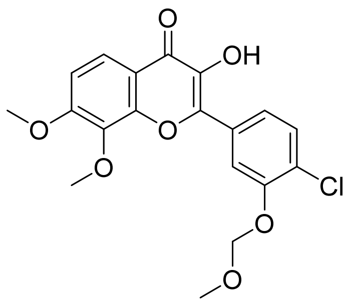

The reaction was performed according to the general procedure 2.4.3. to afford compound 12l as a yellow solid (2.38 g, 33%, eluent: hexane/EtOAc = 7/3); ***R**_f_* 0.48 (hexane/EtOAc = 1/1); **m.p.** 223.2 °C; **^{1}H-NMR (600 MHz, $CDCl_3$)** δ 8.18 (br s, 1H), 7.86 – 8.06 (m, 2H), 7.53 (d, *J* = 8.5 Hz, 1H), 7.00 – 7.15 (m, 2H), 5.36 (s, 2H), 4.05 (s, 3H), 4.02 (s, 3H), 3.58 (s, 3H) ppm; **^{13}C-NMR (151 MHz, $CDCl_3$)** δ 167.9, 163.6, 156.9, 153.2, 150.0, 146.4, 134.0, 131.2, 130.6, 122.2, 121.1, 115.5, 115.0, 112.5, 110.5, 95.6, 61.8, 56.8, 56.7 ppm; **HRMS (ESI)** *m/z* $[M+H]^+$ calculated for $C_{19}H_{18}ClO_7$: 393.0736, observed: 393.0735.

2-(2,3-Dichlorophenyl)-3-hydroxy-7,8-dimethoxy-4*H*-chromen-4-one (12m)

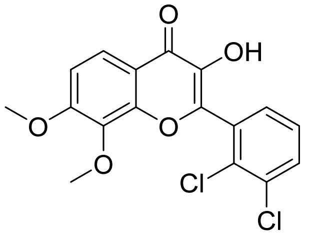

The reaction was performed according to the general procedure 2.4.3. to afford compound 12m as a yellow solid (3.34 g, 46%, eluent: hexane/EtOAc = 7/3); ***R**_f_* 0.25 (hexane/EtOAc = 1/1); **m.p.** 196.8 °C; **^{1}H-NMR (300 MHz, DMSO-d_6)** δ 9.38 (s, 1H), 7.90 (d, *J* = 9.1 Hz, 1H), 7.85 (dd, *J* = 8.1, 1.6 Hz, 1H), 7.70 (dd, *J* = 7.7, 1.6 Hz, 1H), 7.56 (t, *J* = 7.9 Hz, 1H), 7.32 (d, *J* = 9.1 Hz, 1H), 3.97 (s, 3H), 3.82 (s, 3H), 3.57 (s, 3H) ppm; **^{13}C-NMR (75 MHz, DMSO-d_6)** δ 172.6, 156.1, 149.3, 144.8, 138.6, 136.1, 132.3, 132.3, 132.1, 131.0, 130.8, 128.5, 120.4, 116.9, 110.9, 61.0, 56.6 ppm; **HRMS (ESI)** *m/z* $[M+H]^+$ calculated for $C_{17}H_{13}Cl_2O_5$: 367.0135, observed: 367.0139.

2-(3-Chlorophenyl)-3-hydroxy-7,8-dimethoxy-4*H*-chromen-4-one (12n)

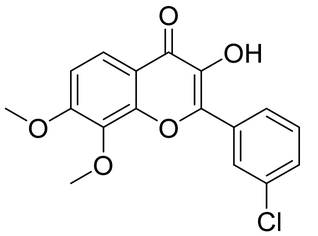

The reaction was performed according to the general procedure 2.4.3. to afford compound 12n as a yellow solid (2.77 g, 33%, eluent: hexane/EtOAc = 7/3); ***R**_f_* 0.43 (hexane/EtOAc = 1/1); **m.p.** 187.5 °C; **^{1}H-NMR (300 MHz, DMSO-d_6)** δ 9.86 (s, 1H), 8.24 (s, 1H), 8.14 (d, *J* = 6.5 Hz, 1H), 7.86 (d, *J* = 8.5 Hz, 1H), 7.52 – 7.70 (m, 2H), 7.30 (d, *J* = 8.7 Hz, 1H), 3.98 (s, 3H), 3.95 (s, 3H) ppm; **^{13}C-NMR (75 MHz, DMSO-d_6)** δ 172.7, 156.2, 148.8, 142.7, 139.0, 136.0, 133.5, 133.3, 130.6, 129.3, 126.8, 125.5, 120.3, 116.1, 110.8, 61.0, 56.5 ppm; **HRMS (ESI)** *m/z* $[M+H]^+$ calculated for $C_{17}H_{14}ClO_5$: 333.0524, observed: 333.0524.

2.4.4. General procedure for methylation of flavonols

To a solution of the respective flavonol (12a-n, 1.00 equiv.) and caesium carbonate (2.25 equiv.) in *N,N*-dimethylformamide (6-8 mL/mmol) was added iodomethane (1.50 equiv.). At ambient temperature the reaction mixture was stirred until complete conversion of the starting material. The mixture was diluted with EtOAc (20 mL/mmol) and the organic phase was washed six times with saturated NaCl solution (20 mL/mmol). The organic phase was dried over Na_2SO_4, filtered, concentrated, and the residue purified by flash chromatography on silica gel to afford the desired product.

2-(3-Chloro-2-(methoxymethoxy)phenyl)-3,7,8-trimethoxy-4*H*-chromen-4-one (16)

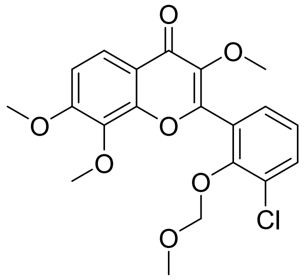

The reaction was performed according to the general procedure 2.4.4. to afford compound 16 as a yellow oil (4.12 g, 80%, eluent: hexane/EtOAc = 7/3); ***R**$_f$* 0.23 (hexane/EtOAc = 3/2); **^{1}H-NMR (300 MHz, DMSO-*d*$_6$)** δ 7.86 (d, *J* = 9.1 Hz, 1H), 7.74 (dd, *J* = 8.0, 1.6 Hz, 1H), 7.59 (dd, *J* = 7.7, 1.7 Hz, 1H), 7.25 – 7.41 (m, 2H), 5.01 (s, 2H), 3.96 (s, 3H), 3.81 (s, 3H), 3.74 (s, 3H), 3.10 (s, 3H) ppm; **^{13}C-NMR (75 MHz, DMSO-*d*$_6$)** δ 173.1, 156.3, 154.0, 151.1, 149.3, 140.5, 136.1, 132.6, 129.9, 127.4, 127.3, 125.6, 120.4, 118.5, 111.0, 99.3, 60.9, 59.6, 56.6, 56.5 ppm; **HRMS (ESI)** *m/z* $[M+H]^+$ calculated for $C_{20}H_{20}ClO_7$: 407.0892, observed: 407.0891.

2-(3-Bromo-2-(methoxymethoxy)phenyl)-3,7,8-trimethoxy-4*H*-chromen-4-one (16a)

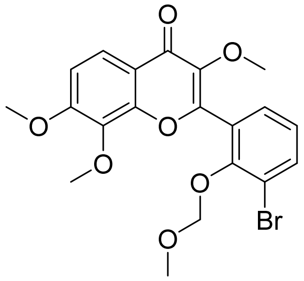

The reaction was performed according to the general procedure 2.4.4. to afford compound 16a as an orange oil (3.28 g, 79%, eluent: hexane/EtOAc = 7/3); ***R**$_f$* 0.21 (hexane/EtOAc = 3/2); **^{1}H-NMR (300 MHz, DMSO-*d*$_6$)** δ 7.81 – 7.94 (m, 2H), 7.62 (dd, *J* = 7.7, 1.6 Hz, 1H), 7.23 – 7.35 (m, 2H), 4.99 (s, 2H), 3.96 (s, 3H), 3.81 (s, 3H), 3.74 (s, 3H), 3.09 (s, 3H) ppm; **^{13}C-NMR (75 MHz, DMSO-*d*$_6$)** δ 173.1, 156.3, 154.1, 152.2, 149.3, 140.5, 136.1, 135.7, 130.6, 127.2, 126.0, 120.4, 118.6, 117.3, 111.0, 99.5, 60.9, 59.5, 56.7, 56.5 ppm; **HRMS (ESI)** *m/z* $[M+H]^+$ calculated for $C_{20}H_{20}BrO_7$: 451.0387, observed: 451.0393.

2-(3-Fluoro-2-(methoxymethoxy)phenyl)-3,7,8-trimethoxy-4*H*-chromen-4-one (16b)

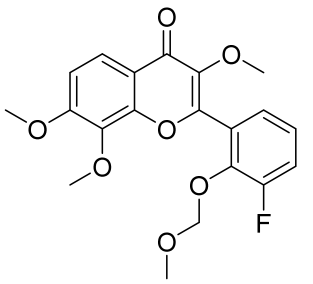

The reaction was performed according to the general procedure 2.4.4. to afford compound 16b as a yellow oil (2.29 g, 83%, eluent: hexane/EtOAc = 7/3); ***R**$_f$* 0.20 (hexane/EtOAc = 3/2); **^{1}H-NMR (300 MHz, DMSO-*d*$_6$)** δ 7.86 (d, *J* = 9.0 Hz, 1H), 7.51 (ddd, *J* = *1*1.4, 8.2, 1.7 Hz, 1H), 7.43 (ddd, *J* = 7.7, 1.7, 1.0 Hz, 1H), 7.27 – 7.38 (m, 2H), 5.07 (d, *J* = 0.7 Hz, 2H), 3.96 (s, 3H), 3.81 (s, 3H), 3.74 (s, 3H), 3.16 (s, 3H) ppm; **^{13}C-NMR (151 MHz, DMSO-*d*$_6$)** δ 173.6, 156.8, 155.4 (d, $^{1}J(^{13}C,^{19}F)$ = 246.0 Hz), 154.1 (d, $^{4}J(^{13}C,^{19}F)$ = 3.0 Hz), 149.8, 142.9 (d, $^{2}J(^{13}C,^{19}F)$ = 12.2 Hz), 141.2, 136.6, 127.4 (d, $^{4}J(^{13}C,^{19}F)$ = 2.3 Hz), 126.9 (d, $^{3}J(^{13}C,^{19}F)$ = 3.3 Hz), 125.3 (d, $^{3}J(^{13}C,^{19}F)$ = 8.1 Hz), 120.8, 119.5 (d, $^{2}J(^{13}C,^{19}F)$ = 19.3 Hz), 119.0, 99.5 (d, $^{4}J(^{13}C,^{19}F)$ = 5.7 Hz), 61.4, 60.2, 57.0, 56.9 ppm; **^{19}F-NMR (565 MHz, DMSO-*d*$_6$)** δ -129.0 ppm; **HRMS (ESI)** *m/z* $[M+H]^+$ calculated for $C_{20}H_{20}FO_7$: 391.1188, observed: 391.1191.

2-(3-Iodo-2-(methoxymethoxy)phenyl)-3,7,8-trimethoxy-4*H*-chromen-4-one (16c)

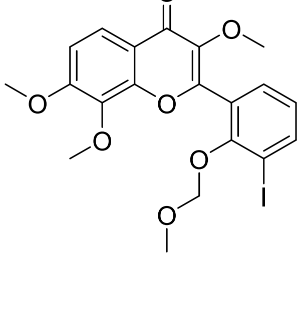

The reaction was performed according to the general procedure 2.4.4. to afford compound 16c as a yellow oil (0.47 g, 76%, eluent: hexane/EtOAc = 7/3); ***R**$_f$* 0.21 (hexane/EtOAc = 3/2); **^{1}H-NMR (600 MHz, $CDCl_3$)** δ 8.01 (dd, *J* = 9.0, 1.0 Hz, 1H), 7.98 (dd, *J* = 7.9, 1.6 Hz, 1H), 7.52 (dd, *J* = 7.6, 1.6 Hz, 1H), 7.07 (d, *J* = 9.0 Hz, 1H), 7.02 (t, *J* = 7.8 Hz, 1H), 5.02 (s, 2H), 4.01 (s, 3H), 3.96 (s, 3H), 3.86 (s, 3H), 3.22 (s, 3H) ppm; **^{13}C-NMR (75 MHz, $CDCl_3$)** δ 174.4, 156.4, 155.5, 155.0, 149.9, 141.9, 141.1, 136.7, 131.4, 126.5, 125.9, 121.1, 119.3, 110.1, 100.3, 93.2, 61.7, 60.3, 57.5, 56.5, 31.6, 14.1 ppm; **HRMS (ESI)** *m/z* $[M+H]^+$ calculated for $C_{20}H_{20}ClO_7$: 499.0248, observed: 499.0242.

3,7,8-Trimethoxy-2-(2-(methoxymethoxy)-3-(trifluoromethyl)phenyl)-4*H*-chromen-4-one (16d)

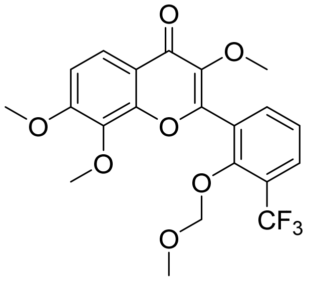

The reaction was performed according to the general procedure 2.4.4. to afford compound 16d as a yellow oil (2.89 g, 53%, eluent: hexane/EtOAc = 7/3); ***Rf*** 0.40 (hexane/EtOAc = 3/2); **^{1}H-NMR (600 MHz, $CDCl_3$)** δ 7.92 (d, *J* = 9.1 Hz, 1H), 7.70 (dd, *J* = 7.9, 1.7 Hz, 1H), 7.64 (dd, *J* = 7.7, 1.7 Hz, 1H), 7.25 – 7.31 (m, 1H), 6.99 (d, *J* = 9.1 Hz, 1H), 4.89 (s, 2H), 3.92 (s, 3H), 3.87 (s, 3H), 3.79 (s, 3H), 3.13 (s, 3H) ppm; **^{13}C-NMR (75 MHz, $CDCl_3$)** δ 174.5, 156.6, 154.3 – 154.8 (m), 150.1, 141.3, 136.9, 135.2, 129.4 (q, $^{3}J(^{13}C,^{19}F)$ = 5.2 Hz), 127.1, 125.1 (q, $^{2}J(^{13}C,^{19}F)$ = 30.6 Hz), 124.7, 124.1, 123.4 (q, $^{1}J(^{13}C,^{19}F)$ = 273.1 Hz), 121.2, 119.5, 110.3, 100.9 – 101.3 (m), 61.7, 60.4, 57.5, 56.6 ppm; **^{19}F-NMR (565 MHz, $CDCl_3$)** δ -60.7 ppm; **HRMS (ESI)** *m/z* $[M+H]^+$ calculated for $C_{21}H_{20}F_3O_7$: 441.1156, observed: 441.1162.

3,7,8-Trimethoxy-2-(2-(methoxymethoxy)-3-methylphenyl)-4*H*-chromen-4-one (16e)

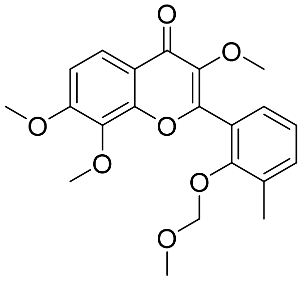

The reaction was performed according to the general procedure 2.4.4. to afford compound 16e as a yellow oil (2.48 g, 70%, eluent: hexane/EtOAc = 7/3); ***Rf*** 0.20 (hexane/EtOAc = 3/2); **^{1}H-NMR (600 MHz, $CDCl_3$)** δ 8.03 (dd, *J* = 9.0, 0.8 Hz, 1H), 7.33 – 7.41 (m, 2H), 7.18 (t, *J* = 7.6 Hz, 1H), 7.07 (dd, *J* = 9.0, 0.8 Hz, 1H), 4.95 (d, *J* = 0.8 Hz, 2H), 4.01 (d, *J* = 0.8 Hz, 3H), 3.96 (d, *J* = 0.8 Hz, 3H), 3.83 (d, *J* = 0.8 Hz, 3H), 3.27 (d, *J* = 0.8 Hz, 3H), 2.41 (s, 3H) ppm; **^{13}C-NMR (75 MHz, DMSO-d_6)** δ 173.2, 156.1, 155.8, 153.8, 149.3, 140.4, 136.1, 133.6, 131.7, 128.5, 125.2, 124.0, 120.3, 118.6, 110.9, 99.0, 60.9, 59.5, 56.5, 56.3, 16.4 ppm; **HRMS (ESI)** *m/z* $[M+H]^+$ calculated for $C_{21}H_{23}O_7$: 387.1438, observed: 387.1438.

2-(3-Bromo-2-fluorophenyl)-3,7,8-trimethoxy-4H-chromen-4-one (16f)

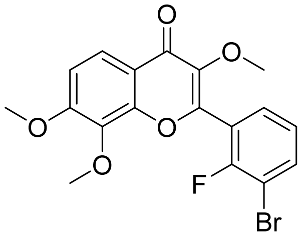

The reaction was performed according to the general procedure 2.4.4. to afford compound 16f as a yellow solid (620 mg, 47%, eluent: hexane/EtOAc = 3/2); ***Rf*** 0.16 (hexane/EtOAc = 7/3); **m.p.** 182.9 °C; **^{1}H-NMR (300 MHz, DMSO-d_6)** δ 7.97 (ddd, *J* = 8.2, 6.7, 1.6 Hz, 1H), 7.87 (d, *J* = 9.0 Hz, 1H), 7.78 (ddd, *J* = 7.9, 6.4, 1.7 Hz, 1H), 7.40 (td, *J* = 7.9, 0.9 Hz, 1H), 7.34 (d, *J* = 9.1 Hz, 1H), 3.97 (s, 3H), 3.83 (s, 3H), 3.79 (s, 3H) ppm; **^{13}C-NMR (75 MHz, DMSO-d_6)** δ 173.08, 156.44, 155.39 (d, $^{1}J(^{13}C,^{19}F)$ = 250.9 Hz), 150.43 (d, $^{4}J(^{13}C,^{19}F)$ = 1.0 Hz), 149.36, 140.97, 136.07 (d, $^{3}J(^{13}C,^{19}F)$ = 13.3 Hz), 130.70 (d, $^{4}J(^{13}C,^{19}F)$ = 1.7 Hz), 126.18 (d, $^{3}J(^{13}C,^{19}F)$ = 4.4 Hz), 120.44, 120.12 (d, $^{2}J(^{13}C,^{19}F)$ = 15.4 Hz), 118.52, 111.22, 108.97 (d, $^{2}J(^{13}C,^{19}F)$ = 21.0 Hz), 60.97, 60.05, 56.60 ppm; **^{19}F-NMR (282 MHz, DMSO-d_6)** δ -106.22 ppm; **HRMS (ESI)** *m/z* $[M+H]^+$ calculated for $C_{18}H_{15}BrFO_5$: 409.0081, observed: 409.0085.

2-(3-Chloro-5-fluoro-2-(methoxymethoxy)phenyl)-3,7,8-trimethoxy-4*H*-chromen-4-one (16h)

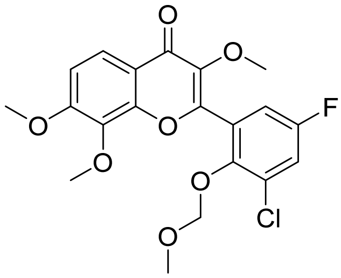

The reaction was performed according to the general procedure 2.4.4. to afford compound 16h as a yellow oil (0.83 g, 67%, eluent: hexane/EtOAc = 7/3); ***Rf*** 0.51 (hexane/EtOAc = 1/1); **^{1}H-NMR (600 MHz, $CDCl_3$)** δ 7.95 (d, *J* = 9.0 Hz, 1H), 7.29 (dd, *J* = 7.8, 3.1 Hz, 1H), 7.17 (dd, *J* = 8.0, 3.1 Hz, 1H), 7.05 (d, *J* = 9.1 Hz,

1H), 5.00 (s, 2H), 3.98 (s, 4H), 3.92 (s, 3H), 3.85 (s, 3H), 3.18 (s, 3H) ppm; **^{13}C-NMR (75 MHz, CDCl$_3$)** δ 174.2, 158.0 (d, $^1J(^{13}C,^{19}F)$ = 247.8 Hz), 156.6, 153.4 (d, $^4J(^{13}C,^{19}F)$ = 2.1 Hz), 149.9, 148.4 (d, $^4J(^{13}C,^{19}F)$ = 3.6 Hz), 141.3, 136.7, 129.4 (d, $^3J(^{13}C,^{19}F)$ = 11.0 Hz), 128.2 (d, $^3J(^{13}C,^{19}F)$ = 9.3 Hz), 121.1, 119.7 (d, $^2J(^{13}C,^{19}F)$ = 25.7 Hz), 119.2, 116.4 (d, $^2J(^{13}C,^{19}F)$ = 23.9 Hz), 110.3, 99.9, 61.6, 60.4, 57.4, 56.5 ppm; **^{19}F-NMR (282 MHz, CDCl$_3$)** δ -115.8 ppm; **HRMS (ESI)** *m/z* [M+H]$^+$ calculated for $C_{20}H_{19}ClFO_7$: 425.0798, observed: 425.0803.

2-(3-Bromo-5-fluoro-2-(methoxymethoxy)phenyl)-3,7,8-trimethoxy-4*H*-chromen-4-one (16i)

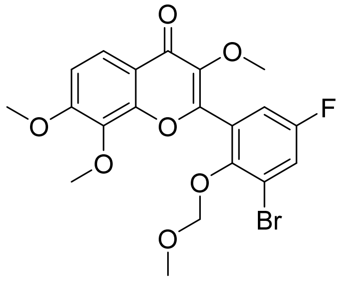

The reaction was performed according to the general procedure 2.4.4. to afford compound 16i as a yellow oil (0.48 g, 78%, eluent: hexane/EtOAc = 7/3); ***R$_f$*** 0.21 (hexane/EtOAc = 3/2); **^{1}H-NMR (600 MHz, CDCl$_3$)** δ 7.93 (d, *J* = 9.0 Hz, 1H), 7.43 (dd, *J* = 7.5, 3.1 Hz, 1H), 7.19 (dd, *J* = 7.9, 3.1 Hz, 1H), 7.02 (d, *J* = 9.0 Hz, 1H), 4.96 (s, 2H), 3.95 (s, 3H), 3.90 (s, 3H), 3.82 (s, 3H), 3.14 (s, 3H) ppm; **^{13}C-NMR (151 MHz, CDCl$_3$)** δ 174.2, 158.1 (d, $^1J(^{13}C,^{19}F)$ = 248.7 Hz), 156.6, 153.5, 149.9, 149.5 (d, $^4J(^{13}C,^{19}F)$ = 3.3 Hz), 141.2, 136.7, 128.1 (d, $^3J(^{13}C,^{19}F)$ = 8.9 Hz), 122.6 (d, $^2J(^{13}C,^{19}F)$ = 25.4 Hz), 121.1, 119.2, 118.5 (d, $^3J(^{13}C,^{19}F)$ = 10.1 Hz), 117.1 (d, $^2J(^{13}C,^{19}F)$ = 23.9 Hz), 110.3, 100.1, 61.6, 60.4, 57.4, 56.5 ppm; **^{19}F-NMR (565 MHz, CDCl$_3$)** δ -115.9 ppm; **HRMS (ESI)** *m/z* [M+H]$^+$ calculated for $C_{20}H_{19}BrFO_7$: 469.0293, observed: 469.0295.

2-(3-Bromo-2-(methoxymethoxy)-5-methylphenyl)-3,7,8-trimethoxy-*4H*-chromen-4-one (16j)

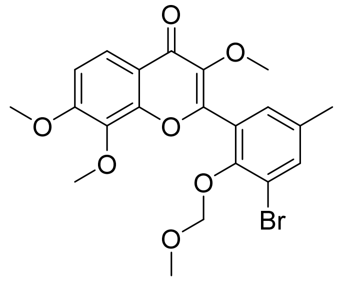

The reaction was performed according to the general procedure 2.4.4. to afford compound 16j as a yellow oil (3.98 g, 95%, eluent: hexane/EtOAc = 7/3); **R$_f$** 0.24 (hexane/EtOAc = 3/2); **^{1}H-NMR (300 MHz**, DMSO-*d$_6$*) δ 7.86 (d, *J* = 9.0 Hz, 1H), 7.72 (dd, *J* = 2.2, 0.9 Hz, 1H), 7.45 – 7.38 (m, 1H), 7.32 (d, *J* = 9.2 Hz, 1H), 4.96 (s, 2H), 3.97 (s, 3H), 3.82 (s, 3H), 3.74 (s, 3H), 3.07 (s, 3H), 2.36 (s, 3H) ppm; **^{13}C-NMR (75 MHz, DMSO-*d$_6$*)** δ 173.13, 156.26, 154.30, 149.88, 149.29, 140.46, 136.12, 135.83, 135.72, 130.75, 126.79, 120.39, 118.55, 116.92, 111.04, 99.42, 60.91, 59.54, 56.65, 56.53, 19.82 ppm; **HRMS (ESI)** *m/z* [M+H]$^+$ calculated for $C_{21}H_{22}BrO_7$: 465.0543, observed: 465.0544.

2-(4-Chloro-2-(methoxymethoxy)phenyl)-3,7,8-trimethoxy-4*H*-chromen-4-one (16k)

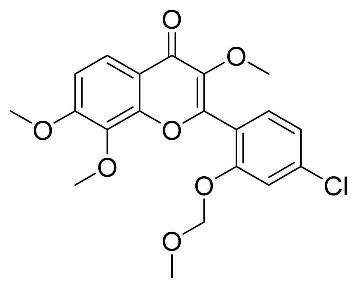

The reaction was performed according to the general procedure 2.4.4. to afford compound 16k as a yellow solid (2.29 g, 84%, eluent: hexane/EtOAc = 7/3); ***R$_f$*** 0.47 (hexane/EtOAc = 1/1); **m.p.** 116.7 °C; **^{1}H-NMR (300 MHz, DMSO-*d$_6$*)** δ 7.86 (d, *J* = 9.0 Hz, 1H), 7.59 (d, *J* = 8.2 Hz, 1H), 7.40 (d, *J* = *1*.9 Hz, 1H), 7.31 (d, *J* = 9.2 Hz, 1H), 7.26 (dd, *J* = 8.2, 1.9 Hz, 1H), 5.30 (s, 2H), 3.96 (s, 4H), 3.80 (s, 3H), 3.71 (s, 3H), 3.34 (s, 3H) ppm; **^{13}C-NMR (75 MHz, DMSO-*d$_6$*)** δ 173.1, 156.2, 155.2, 154.2, 149.3, 140.7, 136.1, 136.0, 132.1, 121.4, 120.3, 119.5, 118.6, 115.3, 111.0, 94.3, 60.9, 59.9, 56.5, 55.8 ppm; **HRMS (ESI)** *m/z* [M+H]$^+$ calculated for $C_{20}H_{20}ClO_7$: 407.0892, observed: 407.0891.

2-(4-Chloro-3-(methoxymethoxy)phenyl)-3,7,8-trimethoxy-4*H*-chromen-4-one (16l)

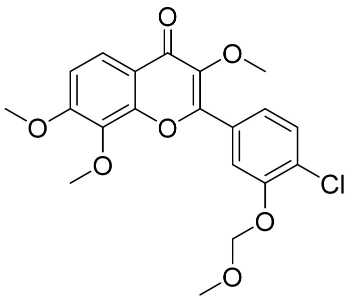

The reaction was performed according to the general procedure 2.4.4. to afford compound 16l as a beige solid (1.93 g, 79%, eluent: hexane/EtOAc = 7/3); ***R**$_f$* 0.21 (hexane/EtOAc = 3/2); **m.p.** 145.2 °C**; ^{1}H-NMR (600 MHz, DMSO-*d*$_6$)** δ 8.00 (d, *J* = 1.9 Hz, 1H), 7.83 (d, *J* = 9.0 Hz, 1H), 7.74 (dd, *J* = 8.4, 1.9 Hz, 1H), 7.70 (d, *J* = 8.4 Hz, 1H), 7.31 (d, *J* = 9.1 Hz, 1H), 5.38 (s, 2H), 3.97 (s, 3H), 3.93 (s, 3H), 3.84 (s, 3H), 3.48 (s, 3H) ppm; **^{13}C-NMR (151 MHz, DMSO-*d*$_6$)** δ 173.5, 156.4, 152.9, 152.3, 148.9, 140.5, 136.1, 130.5, 130.4, 124.8, 122.4, 120.3, 118.2, 115.8, 111.0, 95.0, 61.1, 59.7, 56.5, 56.0 ppm; **HRMS (ESI)** *m/z* $[M+H]^+$ calculated for $C_{20}H_{20}ClO_7$: 407.0892, observed: 407.0983.

2-(2,3-Dichlorophenyl)-3,7,8-trimethoxy-4*H*-chromen-4-one (16m)

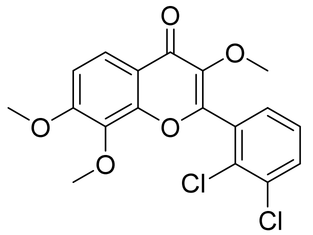

The reaction was performed according to the general procedure 2.4.4. to afford compound 16m as a yellow solid (1.38 g, 74%, eluent: hexane/EtOAc = 7/3); ***R**$_f$* 0.25 (hexane/EtOAc = 3/2); **m.p.** 148.4 °C**; ^{1}H-NMR (600 MHz, DMSO-*d*$_6$)** δ 7.88 (dd, *J* = 8.2, 1.5 Hz, 1H), 7.88 (d, *J* = 9.0 Hz, 1H), 7.74 (dd, *J* = 7.7, 1.5 Hz, 1H), 7.55 – 7.62 (m, 1H), 7.34 (d, *J* = 9.1 Hz, 1H), 3.96 (s, 3H), 3.80 (s, 3H), 3.71 (s, 3H) ppm; **^{13}C-NMR (126 MHz, DMSO-*d*$_6$)** δ 173.1, 156.4, 153.7, 149.2, 140.5, 136.2, 132.4, 132.3, 131.9, 130.6, 130.2, 128.6, 120.3, 118.6, 111.2, 61.0, 59.9, 56.5 ppm; **HRMS (ESI)** *m/z* $[M+H]^+$ calculated for $C_{18}H_{15}Cl_2O_5$: 381.0291, observed: 381.0295.

2-(3-Chlorophenyl)-3,7,8-trimethoxy-4*H*-chromen-4-one (16n)

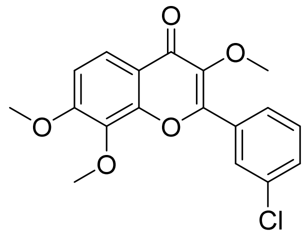

The reaction was performed according to the general procedure 2.4.4. to afford compound 16n as a yellow solid (0.92 g, 72%, eluent: hexane/EtOAc = 7/3); ***R**$_f$* 0.25 (hexane/EtOAc = 3/2); **m.p.** 152.7 °C**; ^{1}H-NMR (600 MHz, DMSO-*d*$_6$)** δ 8.02 (td, *J* = 1.7, 0.8 Hz, 1H), 7.99 (ddd, *J* = 6.4, 2.4, 1.7 Hz, 1H), 7.82 (d, *J* = 9.0 Hz, 1H), 7.61 – 7.67 (m, 2H), 7.30 (d, *J* = 9.0 Hz, 1H), 3.96 (s, 3H), 3.91 (s, 3H), 3.84 (s, 3H) ppm; **^{13}C-NMR (75 MHz, DMSO-*d*$_6$)** δ 173.4, 156.4, 152.7, 148.9, 140.6, 136.1, 133.3, 132.6, 130.7, 130.5, 127.6, 126.7, 120.3, 118.2, 111.0, 61.0, 59.8, 56.5 ppm; **HRMS (ESI)** *m/z* $[M+H]^+$ calculated for $C_{18}H_{16}ClO_5$: 347.0681, observed: 347.0685.

2.4.5. General procedure for deprotection of MOM ether

To a solution of the respective MOM-protected flavone (16a-n, 1.00 equiv.) in methanol (9.0 mL/mmol) was added 2 N hydrochloric acid (9.0 mL/mmol). The reaction was stirred at 50 °C until complete conversion of the starting material, as indicated by TLC.

Work-up method A. The reaction mixture was diluted with ice cold water (20 mL/mmol) and the suspension was stored overnight at 7 °C. The precipitated product was filtered off and washed with ice cold water. The filter cake was suspended in ethanol (3.0 mL/mmol) and the solvent was removed by distillation as an azeotrope on a rotary evaporator with a bath temperature of 45 °C. This process was repeated twice, and the product was used for the next step without further purification steps.

Work-up method B. The solvent volume was reduced by half under reduced pressure and the aqueous suspension was extracted four times with dichloromethane (10 mL/mmol). The combined organic phase was washed with

saturated NaCl solution (10 mL/mmol), dried over Na_2SO_4, filtered, concentrated, and the residue was purified by flash chromatography on silica gel or recrystallization to afford the desired product.

2-(3-Chloro-2-hydroxyphenyl)-3,7,8-trimethoxy-4*H*-chromen-4-one (17)

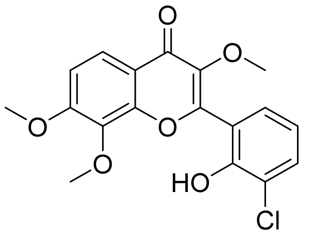

The reaction was performed according to the general procedure 2.4.5. and work-up method B to afford compound 17 as a white solid (2.12 g, 79%, eluent: hexane/EtOAc + 0.1% TEA = 2/3); ***R$_f$*** 0.35 (hexane/EtOAc = 3/1); **m.p.** 77.9 °C; **^{1}H-NMR (600 MHz, DMSO-*d$_6$*)** δ 10.02 (s, 1H), 7.86 (d, *J* = 9.0 Hz, 1H), 7.57 (dd, *J* = 8.0, 1.6 Hz, 1H), 7.42 (dd, *J* = 7.7, 1.6 Hz, 1H), 7.31 (d, *J* = 9.1 Hz, 1H), 7.01 (t, *J* = 7.8 Hz, 1H), 3.96 (s, 3H), 3.81 (s, 3H), 3.72 (s, 3H) ppm; **^{13}C-NMR (75 MHz, DMSO-*d$_6$*)** δ 173.4, 156.1, 154.4, 150.9, 149.6, 140.8, 136.2, 131.9, 129.5, 121.5, 120.4, 120.2, 120.1, 118.8, 110.8, 60.9, 59.8, 56.5 ppm; **HRMS (ESI)** *m/z* $[M+H]^+$ calculated for $C_{18}H_{16}ClO_6$: 363.0630, observed: 363.0632.

2-(3-Bromo-2-hydroxyphenyl)-3,7,8-trimethoxy-4*H*-chromen-4-one (17a)

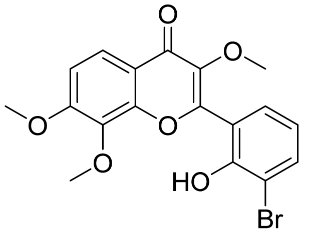

The reaction was performed according to the general procedure 2.4.5. and work-up method B to afford compound 17a as a white solid (1.51 g, 83%, eluent: hexane/EtOAc + 0.1% TEA = 3/2); ***R$_f$*** 0.41 (hexane/EtOAc = 1/1); **m.p.** 176.3 °C; **^{1}H-NMR (300 MHz, DMSO-*d$_6$*)** δ 9.98 (s, 1H), 7.85 (d, *J* = 9.0 Hz, 1H), 7.72 (dd, *J* = 8.0, 1.6 Hz, 1H), 7.44 (dd, *J* = 7.7, 1.6 Hz, 1H), 7.30 (d, *J* = 9.1 Hz, 1H), 6.94 (t, *J* = 7.8 Hz, 1H), 3.96 (s, 3H), 3.80 (s, 3H), 3.71 (s, 3H) ppm; **^{13}C-NMR (75 MHz, DMSO-*d$_6$*)** δ 173.5, 156.1, 154.3, 151.7, 149.6, 140.9, 136.2, 135.0, 130.2, 120.6, 120.2, 120.1, 118.9, 111.5, 110.7, 60.9, 59.8, 56.5 ppm; **HRMS (ESI)** *m/z* $[M+H]^+$ calculated for $C_{18}H_{16}BrO_6$: 407.0125, observed: 407.0129.

2-(3-Fluoro-2-hydroxyphenyl)-3,7,8-trimethoxy-4*H*-chromen-4-one (17b)

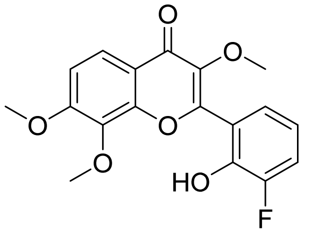

The reaction was performed according to the general procedure 2.4.5. and work-up method B to afford compound 17b as a white solid (0.86 g, 79%, eluent: hexane/EtOAc + 0.1% TEA = 1/1); ***R$_f$*** 0.37 (hexane/EtOAc = 1/1); **m.p.** 130.9 °C; **^{1}H-NMR (300 MHz, DMSO-*d$_6$*)** δ 10.18 (s, 1H), 7.86 (d, *J* = 9.0 Hz, 1H), 7.38 (ddd, *J* = 11.2, 8.2, 1.6 Hz, 1H), 7.31 (d, *J* = 9.1 Hz, 1H), 7.27 (dt, *J* = 7.8, 1.4 Hz, 1H), 6.97 (td, *J* = 8.0, 4.9 Hz, 1H), 3.96 (s, 3H), 3.82 (s, 3H), 3.72 (s, 3H) ppm; **^{13}C-NMR (75 MHz, DMSO-*d$_6$*)** δ 173.3, 156.1, 154.2 (d, $^4J(^{13}C,^{19}F)$ = 3.5 Hz), 151.6 (d, $^1J(^{13}C,^{19}F)$ = 240.2 Hz), 149.5, 143.4 (d, $^2J(^{13}C,^{19}F)$ = 14.8 Hz), 140.7, 136.2, 125.9 (d, $^4J(^{13}C,^{19}F)$ = 3.2 Hz), 121.0 (d, $^4J(^{13}C,^{19}F)$ = 3.4 Hz), 120.2, 119.1 (d, $^3J(^{13}C,^{19}F)$ = 7.3 Hz), 118.7, 117.8 (d, $^2J(^{13}C,^{19}F)$ = 18.5 Hz), 110.8, 60.9, 59.8, 56.5 ppm; **^{19}F-NMR (282 MHz, DMSO-*d$_6$*)** δ -134.5 ppm; **HRMS (ESI)** *m/z* $[M+H]^+$ calculated for $C_{18}H_{16}FO_6$: 347.0925, observed: 347.0930.

2-(2-Hydroxy-3-iodophenyl)-3,7,8-trimethoxy-4*H*-chromen-4-one (17c)

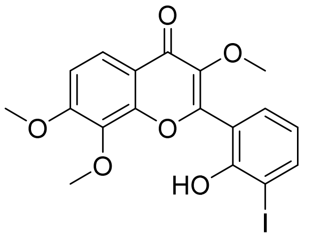

The reaction was performed according to the general procedure 2.4.5. and work-up method A to afford compound 17c as a yellow solid (341 mg, 87%); ***R$_f$*** 0.43 (hexane/EtOAc = 1/1); **m.p.** 177.4 °C; **^{1}H-NMR (600 MHz, $CDCl_3$)** δ 8.49 (s, 1H), 8.00 (d, *J* = 9.0 Hz, 1H), 7.99 (dd, *J* = 7.7, 1.6 Hz, 1H), 7.78 (dd, *J* = 7.9,

1.6 Hz, 1H), 7.08 (d, J = 9.0 Hz, 1H), 6.87 (t, J = 7.8 Hz, 1H), 4.01 (s, 3H), 3.96 (s, 3H), 3.93 (s, 3H) ppm; **^{13}C-NMR (151 MHz, CDCl$_3$)** δ 173.4, 156.8, 154.0, 153.9, 150.1, 142.6, 139.2, 136.9, 130.3, 122.4, 121.1, 118.8, 118.8, 110.4, 88.5, 62.1, 61.7, 56.6 ppm; **HRMS (ESI)** *m/z* $[M+H]^+$ calculated for $C_{18}H_{16}IO_6$: 454.9986, observed: 454.9990.

2-(2-Hydroxy-3-(trifluoromethyl)phenyl)-3,7,8-trimethoxy-4*H*-chromen-4-one (17d)

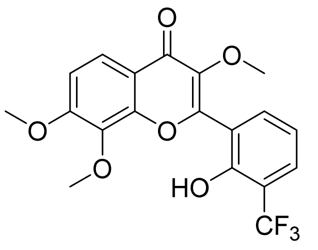

The reaction was performed according to the general procedure 2.4.5. and work-up method B to afford compound 17d as a white solid (2.14 g, 68%, eluent: hexane/EtOAc = 3/2); ***R$_f$*** 0.45 (hexane/EtOAc = 1/1); **m.p.** 167.8 °C; **^{1}H-NMR (600 MHz, CDCl$_3$)** δ 8.50 (s, 1H), 7.93 (dd, J = 8.3, 6.2 Hz, 2H), 7.73 (dd, J = 7.8, 1.6 Hz, 1H), 7.14 (t, J = 7.8 Hz, 1H), 7.02 (d, J = 9.1 Hz, 1H), 3.95 (s, 3H), 3.89 (s, 3H), 3.89 (s, 3H) ppm; **^{13}C-NMR (151 MHz, CDCl$_3$)** δ 173.5, 157.1, 154.0, 153.5, 150.3, 139.3, 137.1, 133.7, 130.6 (d, $^3J(^{13}C,^{19}F)$ = 5.0 Hz), 124.5, 121.4, 121.1, 120.8, 120.5, 118.8, 110.6, 62.5, 61.8, 56.8 ppm; **^{19}F-NMR (565 MHz, CDCl$_3$)** δ -62.5 ppm; **HRMS (ESI)** *m/z* $[M+H]^+$ calculated for $C_{19}H_{16}F_3O_7$: 397.0893, observed: 397.0895.

2-(2-Hydroxy-3-methylphenyl)-3,7,8-trimethoxy-4*H*-chromen-4-one (17e)

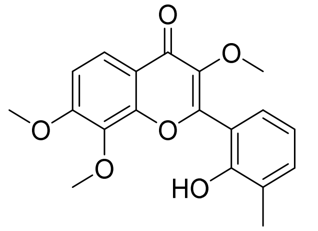

The reaction was performed according to the general procedure 2.4.5. and work-up method B to afford compound 17e as a white solid (1.22 g, 69%, recrystallization: hexane/EtOAc); ***R$_f$*** 0.29 (hexane/EtOAc = 1/1); **m.p.** 145.9 °C; **^{1}H-NMR (300 MHz, DMSO-*d*$_6$)** δ 9.04 (s, 1H), 7.84 (d, J = 9.0 Hz, 1H), 7.28 (dd, J = 8.5, 3.2 Hz, 2H), 7.23 (dd, J = 8.1, 1.6 Hz, 1H), 6.88 (t, J = 7.5 Hz, 1H), 3.95 (s, 3H), 3.80 (s, 3H), 3.69 (s, 3H), 2.24 (s, 3H) ppm; **^{13}C-NMR (75 MHz, DMSO-*d*$_6$)** δ 173.5, 156.0, 155.9, 153.0, 149.6, 140.7, 136.2, 132.8, 128.1, 125.8, 120.1, 118.9, 118.9, 118.2, 110.6, 60.9, 59.7, 56.5, 16.5 ppm; **HRMS (ESI)** *m/z* $[M+H]^+$ calculated for $C_{19}H_{19}O_6$: 343.1176, observed: 343.1182.

2-(3-Chloro-5-fluoro-2-hydroxyphenyl)-3,7,8-trimethoxy-4*H*-chromen-4-one (17h)

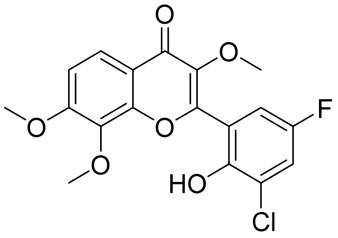

The reaction was performed according to the general procedure 2.4.5. and work-up method B to afford compound 17h as a yellow solid (342 mg, 64%, eluent: hexane/EtOAc = 3/2); ***R$_f$*** 0.16 (hexane/EtOAc = 3/2); **m.p.** 165.3 °C; **^{1}H-NMR (300 MHz, DMSO-*d*$_6$)** δ 10.02 (s, 1H), 7.85 (d, J = 9.1 Hz, 1H), 7.61 (dd, J = 8.3, 3.2 Hz, 1H), 7.38 (dd, J = 8.6, 3.2 Hz, 1H), 7.31 (d, J = 9.1 Hz, 1H), 3.96 (s, 3H), 3.81 (s, 3H), 3.73 (s, 3H) ppm; **^{13}C-NMR (75 MHz, DMSO-*d*$_6$)** δ 173.4, 156.2, 154.1 (d, $^1J(^{13}C,^{19}F)$ = 239.0 Hz), 153.0 (d, $^4J(^{13}C,^{19}F)$ = 1.5 Hz), 149.6, 147.8 (d, $^4J(^{13}C,^{19}F)$ = 2.8 Hz), 140.9, 136.2, 122.2 (d, $^3J(^{13}C,^{19}F)$ = 11.1 Hz), 120.6 (d, $^3J(^{13}C,^{19}F)$ = 9.3 Hz), 120.2, 119.2, 118.8, 116.0 (d, $^2J(^{13}C,^{19}F)$ = 23.9 Hz), 110.9, 61.0, 59.8, 56.6 ppm; **^{19}F-NMR (282 MHz, DMSO-*d*$_6$)** δ -123.0 ppm; **HRMS (ESI)** *m/z* $[M+H]^+$ calculated for $C_{18}H_{15}ClFO_6$: 381.0536, observed: 381.0536.

2-(3-Bromo-5-fluoro-2-hydroxyphenyl)-3,7,8-trimethoxy-4*H*-chromen-4-one (17i)

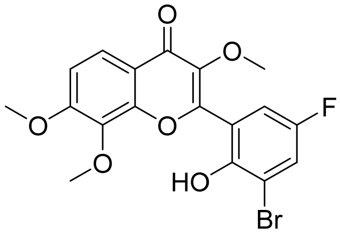

The reaction was performed according to the general procedure 2.4.5. and work-up method A to afford compound 17i as a yellow solid (257 mg, 69%); ***R**_f* 0.25 (hexane/EtOAc = 3/2); **m.p.** 173.8 °C; **^{1}H-NMR (300 MHz, CDCl$_3$)** δ 8.11 (br s, 1H), 7.99 (d, *J* = 9.0 Hz, 1H), 7.41 – 7.56 (m, 2H), 7.08 (d, *J* = 9.1 Hz, 1H), 4.01 (s, 3H), 3.97 (s, 3H), 3.95 (s, 3H) ppm; **^{13}C-NMR (75 MHz, CDCl$_3$)** δ 173.6, 157.2, 156.0 (d, $^1J(^{13}C,^{19}F)$ = 243.4 Hz), 152.8 (d, $^4J(^{13}C,^{19}F)$ = 1.4 Hz), 150.2, 148.6 (d, $^4J(^{13}C,^{19}F)$ = 2.7 Hz), 139.8, 137.0, 123.6 (d, $^2J(^{13}C,^{19}F)$ = 25.8 Hz), 121.3, 120.2 (d, $^3J(^{13}C,^{19}F)$ = 8.2 Hz), 118.9, 115.4 (d, $^2J(^{13}C,^{19}F)$ = 24.5 Hz), 114.1 (d, $^3J(^{13}C,^{19}F)$ = 10.1 Hz), 110.7, 62.4, 61.9, 56.8 ppm; **^{19}F-NMR (282 MHz, CDCl$_3$)** δ -121.3 ppm; **HRMS (ESI)** *m/z* [M+H]$^+$ calculated for $C_{18}H_{15}BrFO_6$: 425.0031, observed: 425.0034.

2-(3-Bromo-2-hydroxy-5-methylphenyl)-3,7,8-trimethoxy-*4H*-chromen-4-one (17j)

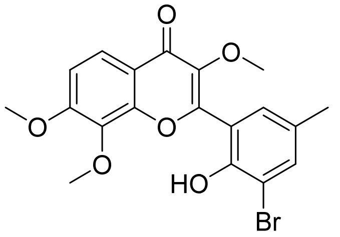

The reaction was performed according to the general procedure 2.4.5. and work-up method A to afford compound 17j as a white solid (2.49 g, 74%); ***R**_f* 0.33 (hexane/EtOAc = 1/1); **m.p.** 174.8 °C**; ^{1}H-NMR (300 MHz, DMSO-*d*$_6$)** δ 9.69 (s, 1H), 7.84 (d, *J* = 9.0 Hz, 1H), 7.55 (dd, *J* = 2.1, 0.8 Hz, 1H), 7.30 (d, *J* = 9.1 Hz, 1H), 7.22 (dd, *J* = 2.2, 0.8 Hz, 1H), 3.95 (s, 3H), 3.80 (s, 3H), 3.70 (s, 3H), 3.29 (s, 1H), 2.27 (d, *J* = 0.8 Hz, 3H) ppm; **^{13}C-NMR (136 MHz, DMSO-*d*$_6$)** δ 173.84, 156.47, 154.88, 150.01, 149.83, 141.28, 136.74, 135.63, 130.64, 130.25, 120.52, 120.33, 119.36, 111.74, 111.24, 61.35, 60.18, 56.98, 19.88 ppm; **(ESI)** *m/z* [M+H]$^+$ calculated for $C_{19}H_{18}BrO_6$: 421.0281, observed: 421.0284.

2-(4-Chloro-2-hydroxyphenyl)-3,7,8-trimethoxy-4*H*-chromen-4-one (17k)

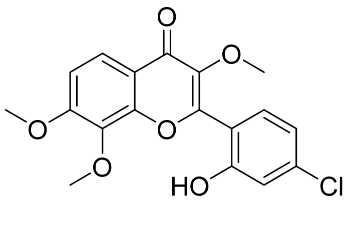

The reaction was performed according to the general procedure 2.4.5. and work-up method B to afford compound 17k as a yellow solid (1.78 g, 95%, eluent: hexane/EtOAc = 7/3); ***R**_f* 0.37 (hexane/EtOAc = 1/1); **m.p.** 194.2 °C; **^{1}H-NMR (600 MHz, DMSO-*d*$_6$)** δ 10.54 (s, 1H), 7.84 (d, *J* = 9.0 Hz, 1H), 7.46 (d, *J* = 8.2 Hz, 1H), 7.29 (d, *J* = 9.1 Hz, 1H), 7.04 (d, *J* = 2.0 Hz, 1H), 7.02 (dd, *J* = 8.1, 2.1 Hz, 1H), 3.95 (s, 3H), 3.81 (s, 3H), 3.71 (s, 3H) ppm; **^{13}C-NMR (75 MHz, DMSO-*d*$_6$)** δ 173.2, 156.6, 156.1, 154.7, 149.4, 140.6, 136.1, 135.6, 132.1, 120.2, 118.9, 118.7, 117.2, 116.0, 110.8, 60.9, 59.7, 56.5 ppm; **HRMS (ESI)** *m/z* [M+H]$^+$ calculated for $C_{18}H_{16}ClO_6$: 363.0630, observed: 363.0626.

2-(4-Chloro-3-hydroxyphenyl)-3,7,8-trimethoxy-4*H*-chromen-4-one (17l)

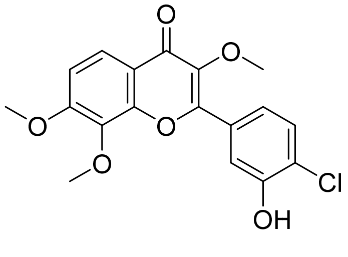

The reaction was performed according to the general procedure 2.4.5. and work-up method B to afford compound 17l as a beige solid (1.38 g, 74%, eluent: hexane/EtOAc = 7/3); ***R**_f* 0.29 (hexane/EtOAc = 1/1); **m.p.** 217.2 °C; **^{1}H-NMR (300 MHz, DMSO-*d*$_6$)** δ 10.69 (s, 1H), 7.81 (d, *J* = 9.0 Hz, 1H), 7.75 (d, *J* = *1*.8 Hz, 1H), 7.47 – 7.60 (m, 2H), 7.29 (d, *J* = 9.1 Hz, 1H), 3.96 (s, 3H), 3.92 (s, 3H), 3.82 (s, 3H) ppm; **^{13}C-NMR (75 MHz, DMSO-*d*$_6$)** δ 173.5, 156.3, 153.2,

153.2, 148.9, 140.4, 136.2, 130.3, 130.2, 122.3, 120.3, 119.7, 118.2, 115.8, 110.9, 61.2, 59.7, 56.5 ppm; **HRMS (ESI)** *m/z* $[M+H]^+$ calculated for $C_{18}H_{16}ClO_6$: 363.0630, observed: 363.0629.

2.4.6. Synthesis of 2-(3-bromo-2-(difluoromethoxy)phenyl)-3,7,8-trimethoxy-4H-chromen-4-one (19)

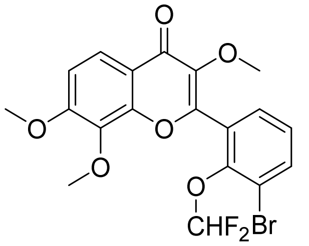

The reaction was performed according to the procedure of Li *et al.*[6] 2-(3-Bromo-2-hydroxyphenyl)-3,7,8-trimethoxy-4H-chromen-4-one (407 mg, 1 mmol, 1.0 equiv.) was dissolved in 4 mL dichloromethane at 0 °C. An aqueous KOH solution (20 wt%, 1.4 mL, 6.0 mmol) was added and stirred vigorously. (Bromodifluoromethyl)trimethyl-silane (518 mg, 2.5 mmol, 2.5 equiv.) was added in three portions after 0, 30 and 60 min under ice bath cooling. The mixture was stirred for 16 h at ambient temperature and the reaction quenched by adding water (5 mL). The aqueous phase was extracted with dichloromethane (2 x 30 mL). The organic layers were combined and dried over anhydrous Na_2SO_4. The solvents were removed in vacuo and the crude product was purified by flash-column chromatography on silica gel (hexane/EtOAc) to afford the titel compound as a white solid (360 mg, 78%, recrystallization: hexane/EtOAc); $\boldsymbol{R_f}$ 0.22 (hexane/EtOAc = 1/1); **m.p.** 116.5 °C; **^{1}H-NMR (300 MHz, $CDCl_3$)** δ δ 8.01 (d, *J* = 9.1 Hz, 1H), 7.80 (dd, *J* = 8.1, 1.6 Hz, 1H), 7.58 (dd, *J* = 7.7, 1.6 Hz, 1H), 7.30 (t, *J* = 7.9 Hz, 1H), 7.07 (d, *J* = 9.1 Hz, 1H), 6.51 (d, *J* = 74.0 Hz, 1H), 4.00 (s, 3H), 3.94 (s, 3H), 3.86 (s, 3H) ppm; **^{13}C-NMR (75 MHz, $CDCl_3$)** δ 174.47, 156.70, 153.07, 150.08, 146.23 (t, $^3J(^{13}C,^{19}F)$ = 3.3 Hz), 141.53, 136.96, 136.03, 131.05, 128.37, 127.88, 121.31, 119.56, 118.23, 116.67 (t, $^1J(^{13}C,^{19}F)$ = 264.8 Hz), 110.31, 61.88, 60.63, 56.66 ppm; **^{19}F-NMR (282 MHz, DMSO-d_6)** δ -80.48. ppm; **HRMS (ESI)** *m/z* $[M+H]^+$ calculated for $C_{19}H_{16}BrF_2O_6$: 457.0093, observed: 457.0100.

2.4.7. General procedure for ruthenium(II)-catalyzed *ortho*-C(sp^2)-H-hydroxylation

To a solution of the respective 3'-methoxyflavonol (1-1n, 1.00 equiv.), selectfluor (1.10 equiv.), silver carbonate (2.00 equiv.) and dichloro(*p*-cymene)ruthenium(II) dimer (5.00mol%) in trifluoroacetic anhydride (15.2 mL/mmol) was added trifluoroacetic acid (3.00 equiv.). The reaction vessel was closed and stirred in a pre-heated oil bath at 80 °C for 24 h. After cooling to ambient temperature dichloromethane (30.0 mL/mmol) was added and the reaction mixture was filtered through a pad of celite that was subsequently washed with multiple small portions of dichloromethane. The filtrate was concentrated under reduced pressure and the residue was dissolved in methanol (50.0 mL/mmol). The solvent was removed on a rotary evaporator with a bath temperature of 50 °C. The crude product was purified by flash chromatography on a short silica gel plug (eluent: hexane/EtOAc = 8/2) and following recrystallization to afford the desired product.

2-(3-Chloro-2-hydroxyphenyl)-5-hydroxy-3,7,8-trimethoxy-4*H*-chromen-4-one (1)

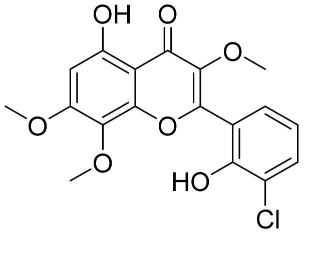

The reaction was performed according to the general procedure 2.4.7. to afford compound 1 as a yellow solid (192 mg, 51%, recrystallization: methanol); $\boldsymbol{R_f}$ 0.34 (hexane/EtOAc = 3/2); **m.p.** 213.7 °C; **^{1}H-NMR (600 MHz, DMSO-d_6)** δ 12.43

(s, 1H), 10.09 (s, 1H), 7.59 (dd, J = 8.0, 1.7 Hz, 1H), 7.42 (dd, J = 7.7, 1.7 Hz, 1H), 7.01 (t, J = 7.8 Hz, 1H), 6.65 (s, 1H), 3.93 (s, 3H), 3.72 (s, 3H), 3.71 (s, 3H) ppm; **^{13}C-NMR (75 MHz, DMSO-*d*$_6$)** δ 178.6, 158.2, 156.5, 155.6, 150.9, 148.7, 139.4, 132.2, 129.5, 128.3, 121.6, 120.2, 119.8, 105.2, 95.8, 60.9, 60.1, 56.6 ppm; **HRMS (ESI)** *m/z* $[M+H]^+$ calculated for $C_{18}H_{16}ClO_7$: 379.0585, observed: 379.0584.

2-(3-Bromo-2-hydroxyphenyl)-5-hydroxy-3,7,8-trimethoxy-4*H*-chromen-4-one (1a)

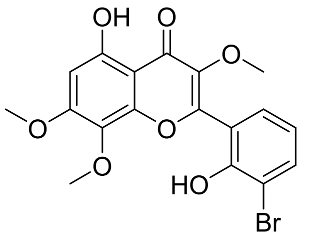

The reaction was performed according to the general procedure 2.4.7. to afford compound 1a as a yellow solid (195 mg, 46%, recrystallization: hexane/EtOAc); ***R$_f$*** 0.24 (hexane/EtOAc = 7/3); **m.p.** 210.2 °C; **^{1}H-NMR (600 MHz, DMSO-*d*$_6$)** δ 12.42 (s, 1H), 10.04 (s, 1H), 7.73 (dd, J = 8.0, 1.6 Hz, 1H), 7.44 (dd, J = 7.6, 1.6 Hz, 1H), 6.95 (t, J = 7.8 Hz, 1H), 6.64 (s, 1H), 3.92 (s, 3H), 3.71 (s, 3H), 3.70 (s, 3H) ppm; **^{13}C-NMR (75 MHz, DMSO-*d*$_6$)** δ 178.7, 158.2, 156.5, 155.6, 151.7, 148.8, 139.5, 135.4, 130.2, 128.4, 120.7, 119.5, 111.6, 105.2, 95.8, 60.9, 60.1, 56.6 ppm; **HRMS (ESI)** *m/z* $[M+H]^+$ calculated for $C_{18}H_{16}BrO_7$: 423.0079, observed: 423.0075.

2-(3-Fluoro-2-hydroxyphenyl)-5-hydroxy-3,7,8-trimethoxy-4*H*-chromen-4-one (1b)

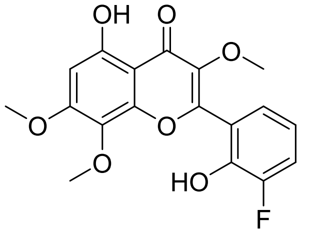

The reaction was performed according to the general procedure 2.4.7. to afford compound 1b as a yellow solid (157 mg, 43%, recrystallization: hexane/EtOAc); ***R$_f$*** 0.35 (hexane/EtOAc = 3/2); **m.p.** 208.6 °C; **^{1}H-NMR (600 MHz, DMSO-*d*$_6$)** δ 12.41 (s, 1H), 10.25 (s, 1H), 7.38 (ddd, J = *1*1.2, 8.2, 1.6 Hz, 1H), 7.27 (dt, J = 7.8, 1.3 Hz, 1H), 6.97 (td, J = 8.0, 4.7 Hz, 1H), 6.63 (s, 1H), 3.92 (s, 3H), 3.73 (s, 3H), 3.70 (s, 3H) ppm; **^{13}C-NMR (126 MHz, DMSO-*d*$_6$)** δ 178.4, 158.1, 156.4, 155.3 (d, $^4J(^{13}C,^{19}F)$ = 3.3 Hz), 151.5 (d, $^1J(^{13}C,^{19}F)$ = 240.7 Hz), 148.5, 143.4 (d, $^2J(^{13}C,^{19}F)$ = 15.1 Hz), 139.2, 128.3, 125.8 (d, $^4J(^{13}C,^{19}F)$ = 3.2 Hz), 120.4 (d, $^3J(^{13}C,^{19}F)$ = 3.7 Hz), 119.1 (d, $^3J(^{13}C,^{19}F)$ = 7.0 Hz), 118.0 (d, $^2J(^{13}C,^{19}F)$ = 18.3 Hz), 105.0, 95.8, 60.8, 60.0, 56.5 ppm; **^{19}F-NMR (282 MHz, DMSO-*d*$_6$)** δ -134.3 ppm; **HRMS (ESI)** *m/z* $[M+H]^+$ calculated for $C_{18}H_{16}FO_7$: 363.0880, observed: 363.0873.

5-Hydroxy-2-(2-hydroxy-3-iodophenyl)-3,7,8-trimethoxy-4*H*-chromen-4-one (1c)

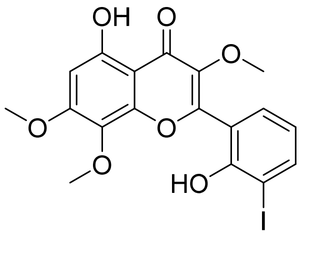

The reaction was performed according to the general procedure 2.4.7. to afford compound 1c as a yellow solid (129 mg, 39%, recrystallization: hexane/EtOAc); ***R$_f$*** 0.36 (hexane/EtOAc = 3/2); **m.p.** 223.4 °C; **^{1}H-NMR (600 MHz, DMSO-*d*$_6$)** δ 12.43 (s, 1H), 10.05 (s, 1H), 7.92 (dd, J = 7.8, 1.7 Hz, 1H), 7.44 (dd, J = 7.6, 1.7 Hz, 1H), 6.79 (t, J = 7.7 Hz, 1H), 6.63 (s, 1H), 3.92 (s, 3H), 3.70 (s, 3H), 3.69 (s, 3H) ppm; **^{13}C-NMR (75 MHz, DMSO-*d*$_6$)** δ 178.7, 158.2, 156.5, 155.7, 153.8, 148.8, 141.5, 139.5, 130.9, 128.4, 121.3, 118.0, 105.3, 95.7, 87.7, 60.9, 60.1, 56.6 ppm; **HRMS (ESI)** *m/z* $[M+H]^+$ calculated for $C_{18}H_{16}IO_7$: 470.9941, observed: 470.9939.

5-Hydroxy-2-(2-hydroxy-3-(trifluoromethyl)phenyl)-3,7,8-trimethoxy-4*H*-chromen-4-one (1d)

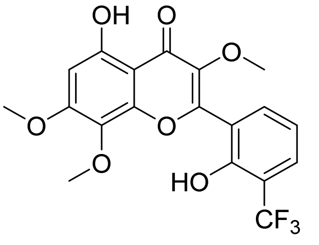

The reaction was performed according to the general procedure 2.4.7. to afford compound 1d as a yellow solid (184 mg, 45%, recrystallization: methanol); ***R****f* 0.34 (hexane/EtOAc = 3/2); **m.p.** 195.4 °C; **^{1}H-NMR (600 MHz, DMSO-*d*$_6$)** δ 12.41 (s, 1H), 10.41 (s, 1H), 7.76 (dd, *J* = 7.9, 1.7 Hz, 1H), 7.71 (dd, *J* = 7.7, 1.7 Hz, 1H), 7.14 (t, *J* = 7.8 Hz, 1H), 6.64 (s, 1H), 3.92 (s, 3H), 3.73 (s, 3H), 3.70 (s, 3H) ppm; **^{13}C-NMR (151 MHz, DMSO-*d*$_6$)** δ 178.8, 158.2, 156.5, 154.6, 152.9 – 153.9 (m), 148.9, 139.9, 135.1, 129.3 (q, $^3J(^{13}C,^{19}F)$ = 5.2 Hz), 128.4, 123.7 (q, $^1J(^{13}C,^{19}F)$ = 272.4 Hz), 119.6, 119.1, 117.7 (q, $^2J(^{13}C,^{19}F)$ = 30.0 Hz), 105.4, 95.8, 60.9, 60.1, 56.6 ppm; **^{19}F-NMR (565 MHz, DMSO-*d*$_6$)** δ -61.0 ppm; **HRMS (ESI)** *m/z* $[M+H]^+$ calculated for $C_{19}H_{16}F_3O_7$: 413.0848, observed: 413.0844.

5-Hydroxy-2-(2-hydroxy-3-methylphenyl)-3,7,8-trimethoxy-4*H*-chromen-4-one (1e)

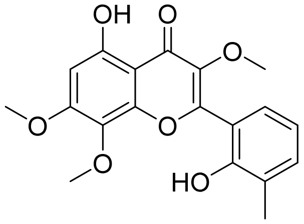

The reaction was performed according to the general procedure 2.4.7. to afford compound 1e as a yellow solid (171 mg, 48%, recrystallization: hexane/EtOAc); ***R****f* 0.40 (hexane/EtOAc = 3/2); **m.p.** 213.3 °C; **^{1}H-NMR (600 MHz, DMSO-*d*$_6$)** δ 12.50 (s, 1H), 9.11 (s, 1H), 7.29 (dd, *J* = 7.5, 1.7 Hz, 1H), 7.23 (dd, *J* = 7.6, 1.7 Hz, 1H), 6.89 (t, *J* = 7.5 Hz, 1H), 6.62 (s, 1H), 3.91 (s, 3H), 3.70 (s, 3H), 3.69 (s, 3H), 2.24 (s, 3H) ppm; **^{13}C-NMR (75 MHz, DMSO-*d*$_6$)** δ 178.7, 158.1, 157.3, 156.5, 153.1, 148.8, 139.3, 133.1, 128.3, 128.0, 125.9, 119.0, 117.7, 105.2, 95.6, 60.9, 60.0, 56.5, 16.4 ppm; **HRMS (ESI)** *m/z* $[M+H]^+$ calculated for $C_{19}H_{19}O_7$: 359.1131, observed: 359.1123.

2-(3-Bromo-2-fluorophenyl)-5-hydroxy-3,7,8-trimethoxy-*4H*-chromen-4-one (1f)

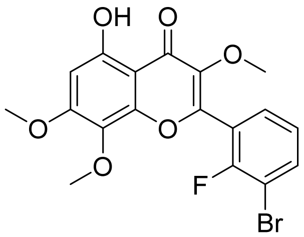

The reaction was performed according to the general procedure 2.4.7. to afford compound 1f as a yellow solid (50 mg, 6%, recrystallization: hexane/EtOAc); ***R****f* 0.45 (hexane/EtOAc = 3/2); **m.p.** 217.4 °C; **^{1}H-NMR (600 MHz, DMSO-*d*$_6$)** δ 12.25 (s, 1H), 7.98 (ddd, *J* = 8.2, 6.7, 1.7 Hz, 1H), 7.77 (ddd, *J* = 7.9, 6.3, 1.7 Hz, 1H), 7.40 (t, *J* = 7.9 Hz, 1H), 6.66 (s, 1H), 3.93 (s, 3H), 3.79 (s, 3H), 3.72 (s, 3H) ppm; **^{13}C-NMR (126 MHz, Acetone-*d*$_6$)** δ 180.08, 160.36, 158.73, 157.38 (d, $^1J(^{13}C,^{19}F)$ = 252.1 Hz), 152.96 (d, $^2J(^{13}C,^{19}F)$ = 8.2 Hz), 150.28, 141.32, 137.30, 131.82 (d, ^4J $(^{13}C,^{19}F)$ = 1.4 Hz), 130.38 (d, $^3J(^{13}C,^{19}F)$ = 5.1 Hz), 126.97 (d, $^3J(^{13}C,^{19}F)$ = 4.6 Hz), 121.63 (d, $^2J(^{13}C,^{19}F)$ = 15.6 Hz), 110.45 (d, ^2J $(^{13}C,^{19}F)$ = 21.0 Hz), 106.78, 97.10, 61.83, 61.28, 57.30 ppm; **^{19}F-NMR (565 MHz, Acetone-*d*$_6$)** δ -106.18 ppm; **HRMS (ESI)** *m/z* $[M+H]^+$ calculated for $C_{18}H_{15}BrFO_6$: 425.0031, observed: 425.0030.

2-(3-Bromo-2-(difluoromethoxy)phenyl)-5-hydroxy-3,7,8-trimethoxy-*4H*-chromen-4-one (1g)

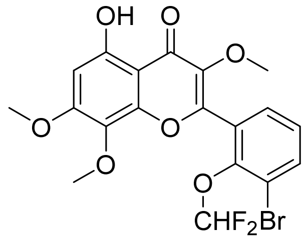

The reaction was performed according to the general procedure 2.4.7. to afford compound 1g as a yellow solid (30 mg, 11%, recrystallization: hexane/EtOAc); ***R****f* 0.27 (hexane/EtOAc = 3/2); **m.p.** 162.4 °C; **^{1}H-NMR (300 MHz, DMSO-*d*$_6$)** δ 12.28 (s, 1H), 8.02 (dd, *J* = 8.1, 1.6 Hz, 1H), 7.76 (dd, *J* = 7.7, 1.6 Hz, 1H), 7.50 (t, *J* = 7.9 Hz, 1H), 7.32 – 6.79 (m, 1H), 6.66 (s, 1H), 3.93 (s, 3H), 3.74 (s, 3H), 3.70 (s, 3H) ppm; **^{13}C-NMR (75 MHz, DMSO-*d*$_6$)** δ 178.27, 158.55, 156.56, 153.63, 148.40, 145.32 (t, $^3J(^{13}C,^{19}F)$ *J* = 3.6 Hz), 139.17, 136.30, 130.90, 128.56, 128.40, 127.02, 117.17 (t, $^1J(^{13}C,^{19}F)$ = 264.0 Hz) 116.98, 105.00, 96.16, 60.91,

60.07, 56.63 ppm; **^{19}F-NMR (282 MHz, DMSO-*d*$_6$)** δ -80.65 ppm; **HRMS (ESI)** *m/z* $[M+H]^+$ calculated for $C_{19}H_{16}BrF_2O_7$: 473.0042, observed: 473.0044.

2-(3-Chloro-5-fluoro-2-hydroxyphenyl)-5-hydroxy-3,7,8-trimethoxy-4*H*-chromen-4-one (1h)

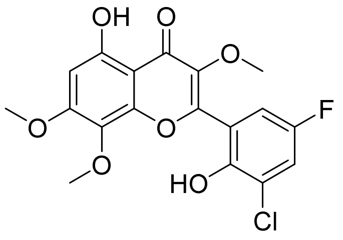

The reaction was performed according to the general procedure 2.4.7. to afford compound 1h as a yellow solid (101 mg, 34%, recrystallization: hexane/EtOAc); ***R$_f$*** 0.34 (hexane/EtOAc = 3/2); **m.p.** 245.0 °C; **^{1}H-NMR (600 MHz, DMSO-*d*$_6$)** δ 12.36 (s, 1H), 10.09 (s, 1H), 7.63 (dd, *J* = 8.2, 3.2 Hz, 1H), 7.38 (dd, *J* = 8.4, 3.2 Hz, 1H), 6.65 (s, 1H), 3.92 (s, 3H), 3.74 (s, 3H), 3.70 (s, 3H) ppm; **^{13}C-NMR (75 MHz, DMSO-*d*$_6$)** δ 178.6, 162.4 (d, $^1J(^{13}C,^{19}F)$ = 238.4 Hz), 158.3, 156.5, 154.2 (d, $^4J(^{13}C,^{19}F)$ = 1.9 Hz), 148.7, 147.9 (d, $^4J(^{13}C,^{19}F)$ = 2.6 Hz), 139.5, 128.4, 122.3 (d, $^3J(^{13}C,^{19}F)$ = 11.2 Hz), 120.0 (d, $^3J(^{13}C,^{19}F)$ = 8.9 Hz), 119.3 (d, $^2J(^{13}C,^{19}F)$ = 25.7 Hz), 115.9 (d, $^2J(^{13}C,^{19}F)$ = 23.9 Hz), 105.2, 95.9, 61.0, 60.2, 56.6 ppm; **^{19}F-NMR (565 MHz, DMSO-*d*$_6$)** δ -122.8 ppm; **HRMS (ESI)** *m/z* $[M+H]^+$ calculated for $C_{18}H_{15}ClFO_7$: 397.0490, observed: 397.0487.

2-(3-Bromo-5-fluoro-2-hydroxyphenyl)-5-hydroxy-3,7,8-trimethoxy-4*H*-chromen-4-one (1i)

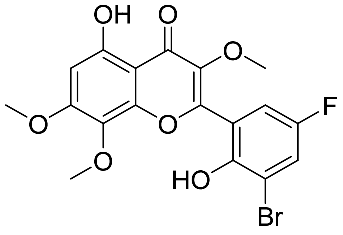

The reaction was performed according to the general procedure 2.4.7. to afford compound 1i as a yellow solid (137 mg, 53%, recrystallization: hexane/EtOAc); ***R$_f$*** 0.38 (hexane/EtOAc = 3/2); **m.p.** 251.4 °C; **^{1}H-NMR (600 MHz, DMSO-*d*$_6$)** δ 12.36 (s, 1H), 10.04 (s, 1H), 7.74 (dd, *J* = 7.9, 3.2 Hz, 1H), 7.41 (dd, *J* = 8.4, 3.2 Hz, 1H), 6.65 (s, 1H), 3.92 (s, 3H), 3.73 (s, 3H), 3.70 (s, 3H) ppm; **^{13}C-NMR (75 MHz, DMSO-*d*$_6$)** δ 178.6, 158.3, 156.5, 154.9, 154.3 (d, $^1J(^{13}C,^{19}F)$ = 239.8 Hz), 154.2 (d, $^4J(^{13}C,^{19}F)$ = 2.0 Hz), 148.7, 139.5, 128.4, 122.2 (d, $^2J(^{13}C,^{19}F)$ = 25.4 Hz), 119.6 (d, $^3J(^{13}C,^{19}F)$ = 8.8 Hz), 116.4 (d, $^2J(^{13}C,^{19}F)$ = 23.7 Hz), 111.9 (d, $^3J(^{13}C,^{19}F)$ = 10.6 Hz), 105.2, 95.9, 61.0, 60.1, 56.6 ppm; **^{19}F-NMR (565 MHz, DMSO-*d*$_6$)** δ -123.0 ppm; **HRMS (ESI)** *m/z* $[M+H]^+$ calculated for $C_{18}H_{15}BrFO_7$: 440.9985, observed: 440.9982.

2-(3-Bromo-2-hydroxy-5-methylphenyl)-5-hydroxy-3,7,8-trimethoxy-4H-chromen-4-one (1j)

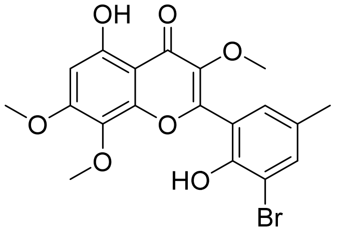

The reaction was performed according to the general procedure 2.4.7. to afford compound 1j as a yellow solid (120 mg, 12%, recrystallization: hexane/EtOAc); ***R$_f$*** 0.40 (hexane/EtOAc = 3/2); **m.p.** 251.8 °C; **^{1}H-NMR (300 MHz, DMSO-*d*$_6$)** δ 12.43 (s, 1H), 9.76 (s, 1H), 7.60 – 7.53 (m, 1H), 7.23 (dd, *J* = 2.2, 0.8 Hz, 1H), 6.63 (s, 1H), 3.92 (s, 3H), 3.71 (s, 3H), 3.69 (s, 3H), 2.27 (s, 3H) ppm; **^{13}C-NMR (136 MHz, DMSO-*d*$_6$)** δ 179.05, 158.62, 156.91, 156.10, 149.88, 149.18, 139.85, 135.95, 130.55, 130.32, 128.89, 119.73, 111.80, 105.67, 96.22, 61.34, 60.53, 56.98, 19.87 ppm; **HRMS (ESI)** *m/z* $[M+H]^+$ calculated for $C_{19}H_{18}BrO_6$: 421.0281, observed: 421.0284.

2-(4-Chloro-2-hydroxyphenyl)-5-hydroxy-3,7,8-trimethoxy-4*H*-chromen-4-one (1k)

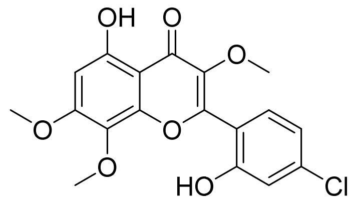

The reaction was performed according to the general procedure 2.4.7. to afford compound 1k as a yellow solid (169 mg, 45%, recrystallization: hexane/EtOAc); ***R**$_f$* 0.29 (hexane/EtOAc = 1/1); **m.p.** 240.7 °C; **^{1}H-NMR (600 MHz, DMSO-*d*$_6$)** δ 12.41 (s, 1H), 10.62 (s, 1H), 7.46 (d, *J* = 8.0 Hz, 1H), 7.03 (d, *J* = 8.2 Hz, 2H), 6.62 (s, 1H), 3.91 (s, 3H), 3.72 (s, 2H), 3.70 (s, 3H) ppm**; ^{13}C-NMR (151 MHz, DMSO-*d*$_6$)** δ 178.9, 158.7, 157.1, 157.0, 156.4, 149.1, 139.6, 136.5, 132.5, 128.8, 119.4, 117.1, 116.6, 105.5, 96.3, 61.4, 60.6, 57.0 ppm; **HRMS (ESI)** *m/z* [M+H]$^+$ calculated for $C_{18}H_{16}ClO_7$: 379.0585, observed: 379.0579.

2-(4-Chloro-3-hydroxyphenyl)-5-hydroxy-3,7,8-trimethoxy-4*H*-chromen-4-one (1l)

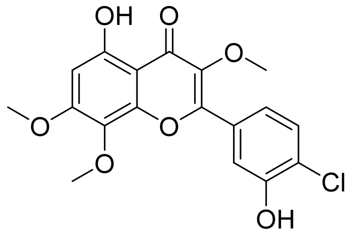

The reaction was performed according to the general procedure 2.4.7. to afford compound 1l as a yellow solid (122 mg, 32%, recrystallization: hexane/EtOAc); ***R**$_f$* 0.28 (hexane/EtOAc = 3/2); **m.p.** 48.0 °C; **^{1}H-NMR (600 MHz, DMSO-*d*$_6$)** δ 12.35 (s, 1H), 10.74 (s, 1H), 7.76 (s, 1H), 7.57 (d, *J* = 8.5 Hz, 1H), 7.49 – 7.56 (m, 1H), 6.62 (s, 1H), 3.93 (s, 3H), 3.84 (s, 3H), 3.82 (s, 3H) ppm; **^{13}C-NMR (75 MHz, DMSO-*d*$_6$)** δ 178.5, 158.4, 156.4, 154.2, 153.2, 147.9, 138.9, 130.3, 129.7, 128.4, 122.8, 119.7, 115.8, 104.7, 95.9, 61.2, 60.0, 56.5 ppm; **HRMS (ESI)** *m/z* [M+H]$^+$ calculated for $C_{18}H_{16}ClO_7$: 379.0585, observed: 379.0581.

2-(2,3-Dichlorophenyl)-5-hydroxy-3,7,8-trimethoxy-4*H*-chromen-4-one (1m)

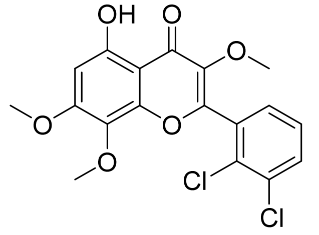

The reaction was performed according to the general procedure 2.4.7. to afford compound 1m as a yellow solid (197 mg, 50%, recrystallization: hexane/EtOAc); ***R**$_f$* 0.32 (hexane/EtOAc = 4/1); **m.p.** 184.8 °C; **^{1}H-NMR (600 MHz, DMSO-*d*$_6$)** δ 12.24 (s,1H), 7.90 (dd, *J* = 8.1, 1.6 Hz, 1H), 7.74 (dd, *J* = 7.7, 1.6 Hz, 1H), 7.59 (t, *J* = 7.9 Hz, 1H), 6.68 (s, 1H), 3.93 (s, 4H), 3.72 (s, 3H), 3.69 (s, 3H) ppm; **^{13}C-NMR (75 MHz, DMSO-*d*$_6$)** δ 178.4, 158.6, 156.6, 154.7, 148.3, 139.1, 132.8, 132.4, 131.4, 130.6, 130.3, 128.8, 128.4, 105.1, 96.3, 61.1, 60.4, 56.7 ppm; **HRMS (ESI)** *m/z* [M+H]$^+$ calculated for $C_{18}H_{15}Cl_2O_6$: 397.0246, observed: 397.0241.

2-(3-Chlorophenyl)-5-hydroxy-3,7,8-trimethoxy-4*H*-chromen-4-one (1n)

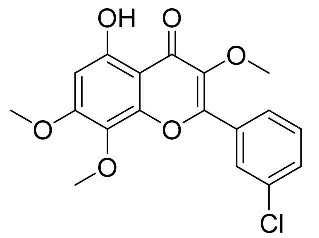

The reaction was performed according to the general procedure 2.4.7. to afford compound 1n as a yellow solid (63 mg, 17%, recrystallization: hexane/EtOAc); ***R**$_f$* 0.40 (hexane/EtOAc = 4/1); **m.p.** 188.3 °C; **^{1}H-NMR (600 MHz, DMSO-*d*$_6$)** δ 12.30 (s, 1H), 8.02 (t, *J* = *1*.9 Hz, 1H), 7.99 (dt, *J* = 7.4, 1.6 Hz, 1H), 7.61 – 7.72 (m, 2H), 6.64 (s, 1H), 3.93 (s, 3H), 3.85 (s, 3H), 3.81 (s, 3H) ppm; **^{13}C-NMR (75 MHz, DMSO-*d*$_6$)** δ 178.5, 158.5, 156.4, 153.7, 148.0, 139.1, 133.4, 132.1, 130.9, 130.8, 128.4, 127.6, 126.8, 104.8, 96.0, 61.0, 60.1, 56.6 ppm; **HRMS (ESI)** *m/z* [M+H]$^+$ calculated for $C_{18}H_{16}ClO_6$: 363.0635, observed: 363.0628.

3. ^{1}H-NMR-Spectra at different pH values of CF and BF

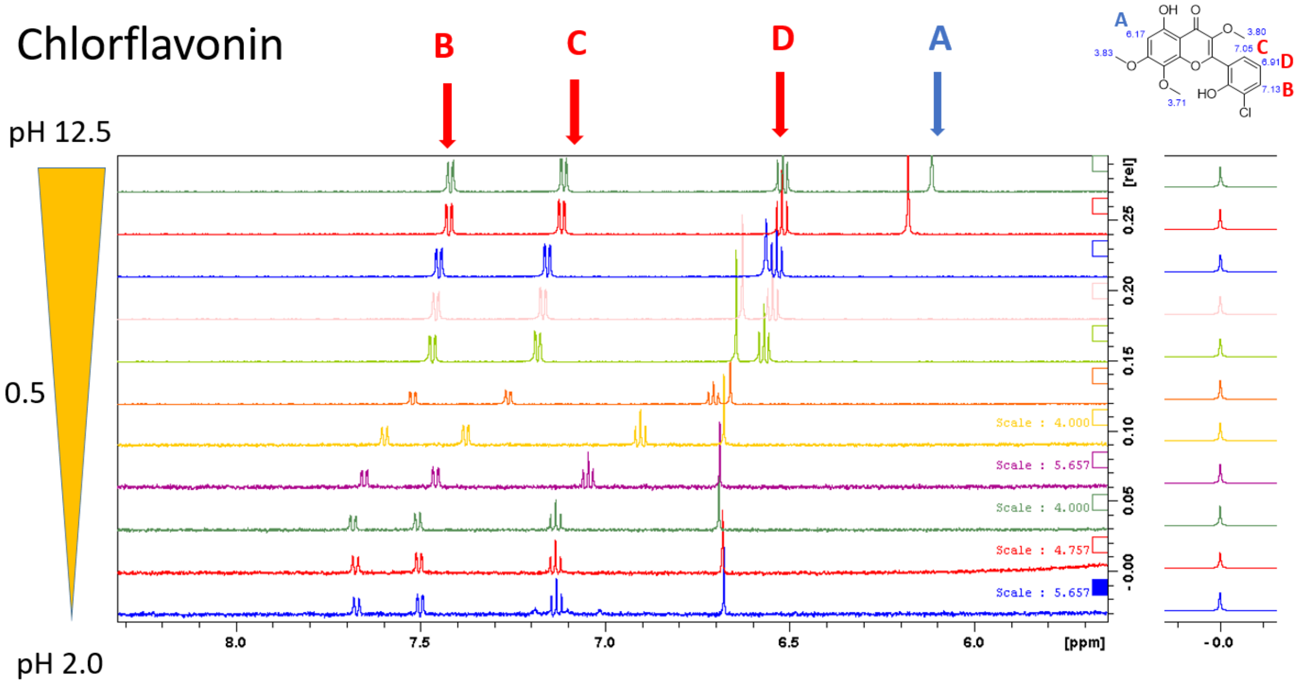

Figure S1: ^{1}H-NMR-Spectra of chlorflavonin's aromatic protons (6-8 ppm) at pH of 2.0-12.5. A-D are the four aromatic protons. Chemical shift in ppm (parts per million).

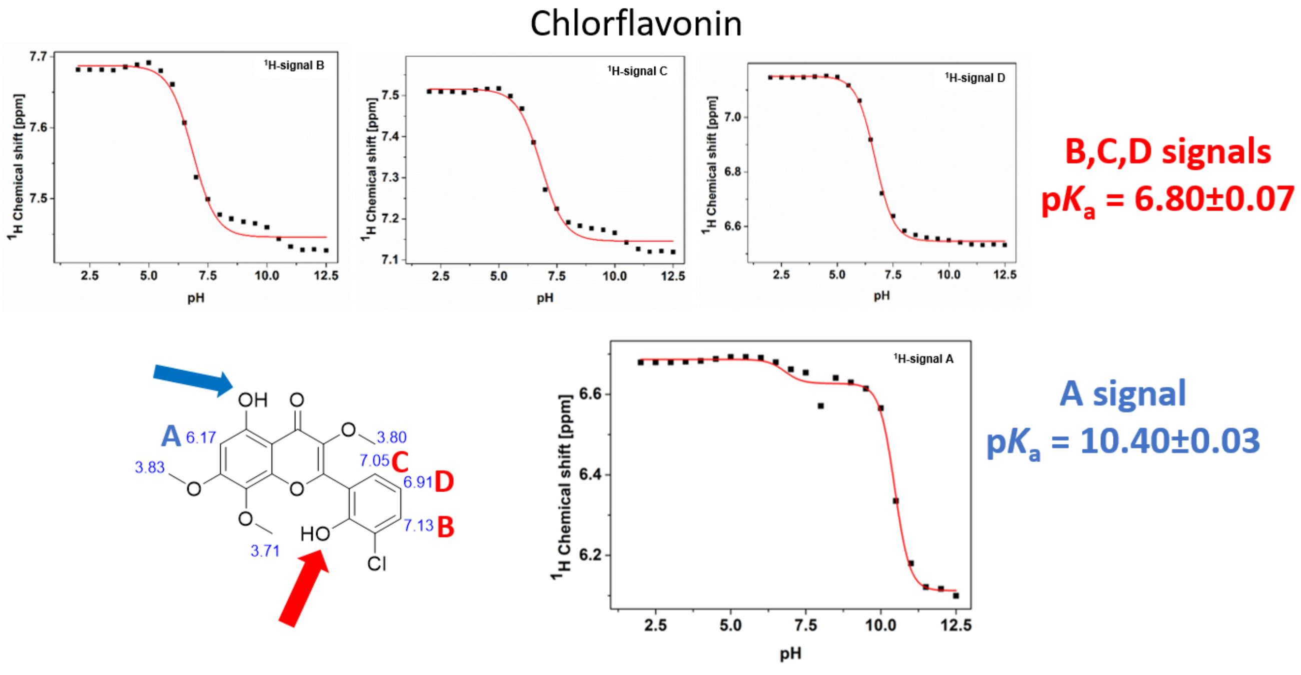

Figure S2: Titration curves (chemical shift in ^{1}H-NMR spectra *vs.* pH value) of chlorflavonin's four aromatic protons plotted with Origin software (OriginLab Corporation, USA)

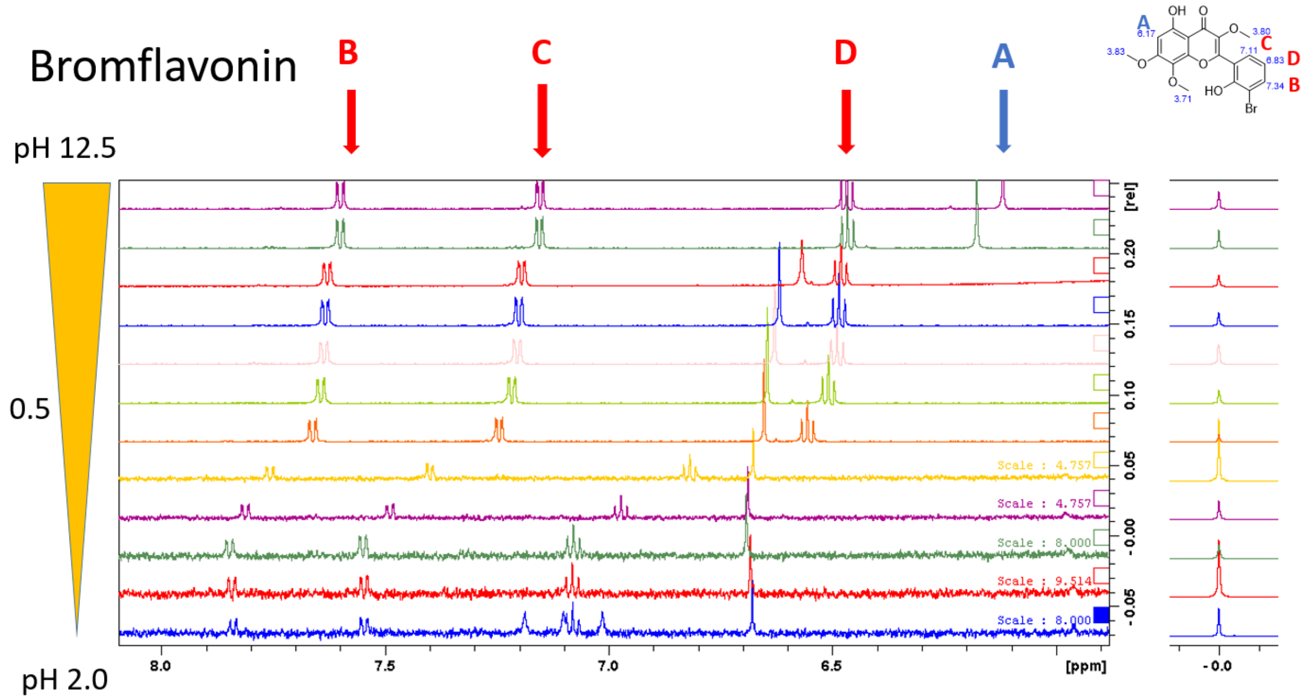

Figure S3: ^{1}H-NMR-Spectra of bromflavonin's aromatic protons (6-8 ppm) at pH of 2.0-12.5. A-D are the four aromatic protons. Chemical shift in ppm (parts per million).

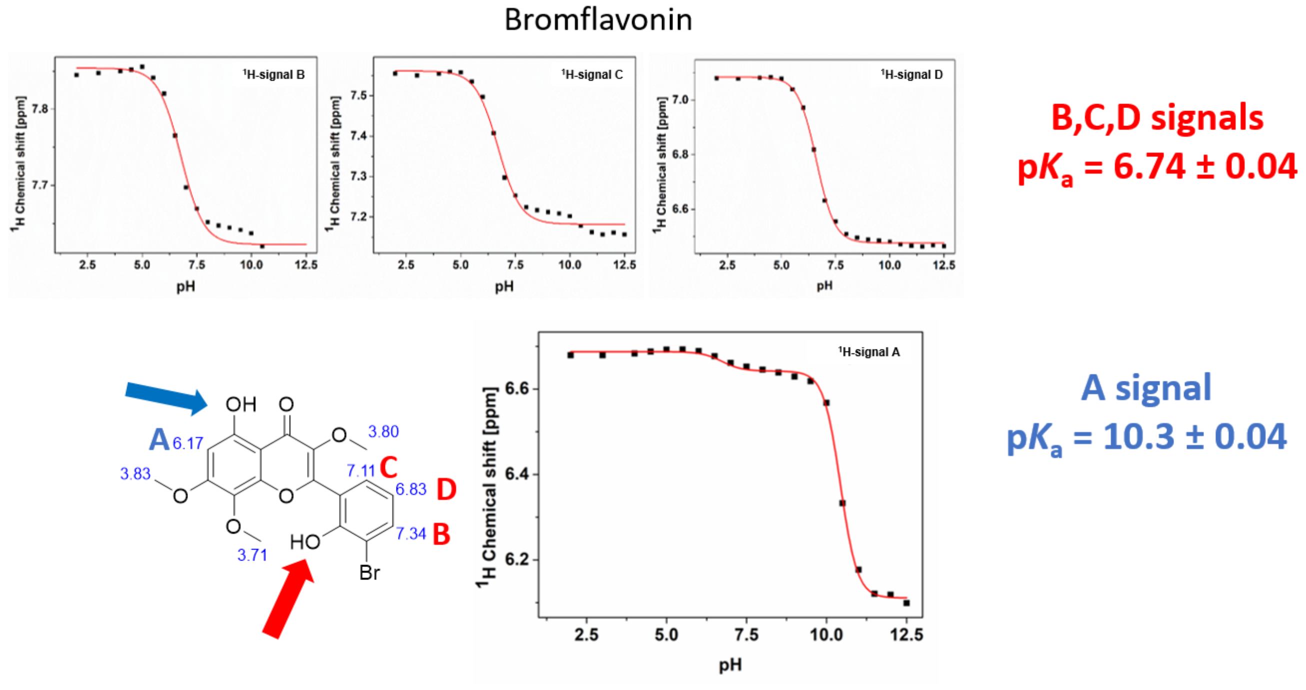

Figure S4: Titration curves (chemical shift in ^{1}H-NMR spectra vs. pH value) of bromflavonin's four aromatic protons plotted with Origin software (OriginLab Corporation, USA).

4. Results of relative free energy calculations

Seven structurally diverse chlorflavonin derivatives with substitutions of ring B that likely bind to AHAS in the predicted binding site1 were chosen to establish a quantitative structure-activity relationship model. Note that the estimated changes in activity among these derivatives are likely < 3 kcal mol^{-1}, which requires using a precise free energy estimation, as is possible with the thermodynamic integration (TI) approach [15,16]. For setting up, performing, and analyzing the TI computations, the TI module of the FEW workflow[9] within Amber21[10] was used. All calculations have converged (Figure S4 and Table S3), and the computed relative free energies ($\Delta\Delta G$) are generally in qualitative agreement with the corresponding relative free energies calculated from difference in minimal inhibitory concentrations (MIC_{90}) (Figure S7). This suggests that changes in MIC_{90} are predominantly determined by differences in the derivatives' affinities. Furthermore, such computations may be used for suggesting further structural changes to obtain more active chlorflavonin derivatives. Exceptions are the transitions **1** -> **1i** and **1** -> **1d**, where the $\Delta\Delta G$ indicates more favorable binding in contrast to the change in MIC_{90}. To explore these cases further, the solubility and octanol/water partition coefficient of all seven derivatives were computed (Table S4) with the QikProp module[14] of the Schrödinger suite. Compounds **1i** and **1d** have the lowest predicted solubility and highest octanol/water partition coefficient among the derivatives, suggesting that differential membrane permeability might underlie the deviation between $\Delta\Delta G$ and the change in MIC_{90}.

Table S3. Relative free energy of binding calculated with the FEW free energy workflow.[9] All values of cycle closure hysteresis are below the threshold of chemical accuracy (1 kcal mol^{-1}).[17]

<table>
<tr><th>Transition</th><th>ΔΔG (for replica) [a]</th><th>$\overline{\Delta\Delta G}$ [a][b]</th><th>Cycle closure hysteresis [a]</th></tr>
<tr><td rowspan="3">1 -> 1a</td><td>-0.84</td><td rowspan="3">-0.71±0.12</td><td rowspan="9">0.15</td></tr>
<tr><td>-0.80</td></tr>
<tr><td>-0.47</td></tr>
<tr><td rowspan="3">1a -> 1c</td><td>4.01</td><td rowspan="3">2.99±0.51</td></tr>
<tr><td>2.42</td></tr>
<tr><td>2.52</td></tr>
<tr><td rowspan="3">1 -> 1c</td><td>2.52</td><td rowspan="3">2.13±0.28</td></tr>
<tr><td>1.60</td></tr>
<tr><td>2.28</td></tr>
<tr><td rowspan="3">1 -> 1i</td><td>-0.64</td><td rowspan="3">-0.88±0.12</td><td rowspan="9">0.56</td></tr>
<tr><td>-1.03</td></tr>
<tr><td>-0.97</td></tr>
<tr><td rowspan="3">1i -> 1d</td><td>0.24</td><td rowspan="3">0.12±0.06</td></tr>
<tr><td>0.03</td></tr>
<tr><td>0.09</td></tr>
<tr><td rowspan="3">1 -> 1d</td><td>-1.07</td><td rowspan="3">-1.32±0.31</td></tr>
<tr><td>-1.93</td></tr>
<tr><td>-0.95</td></tr>
<tr><td rowspan="2">1 -> 1f</td><td>1.39</td><td rowspan="2">0.70±0.43</td><td rowspan="2">0.21</td></tr>
<tr><td>0.81</td></tr>
</table>

	-0.09		
**1-> 1b**	0.69	0.98±0.37	
	0.54		
	1.72		
**1b -> 1f**	-0.20	0.49±0.45	
	0.32		
	1.34		

[a] In kcal mol^{-1}. [b] Mean ± standard error of the mean (SEM). The SEM was computed according to the laws of error propagation.

Table S4. Predicted solubility and octanol/water partition coefficient computed with QikProp [14] for chlorflavonin derivatives included in the relative free energy computations.

Compound	QPlogS [a]	QPlogPo/w[b]
**1**	-4.13	3.35
**1a**	-4.24	3.43
**1b**	-3.79	3.07
**1c**	-4.36	3.51
**1d**	-5.07	3.80
**1f**	-4.13	3.19
**1i**	-4.48	3.58

[a] Predicted aqueous solubility, log *S*. *S* in mol dm^{-3} is the concentration of the solute in a saturated solution that is in equilibrium with the crystalline solid. [b] Predicted octanol/water partition coefficient.

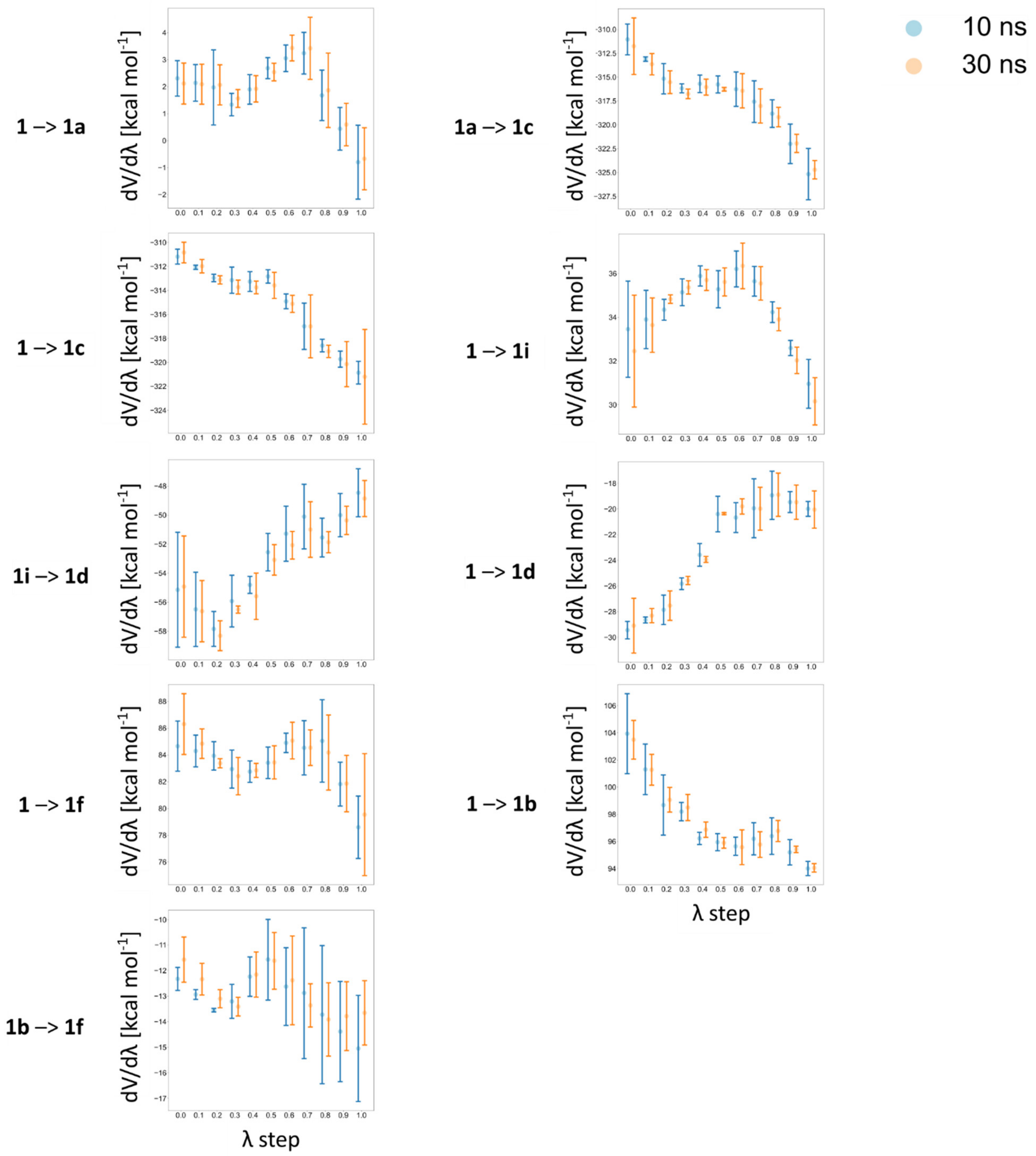

Figure S5. Average dV/dλ over three replicas of free energy calculations of complexes with respect to the simulation time (10 ns per λ step and 30 ns per λ step). Error bars denote the standard deviation from the three replicas.

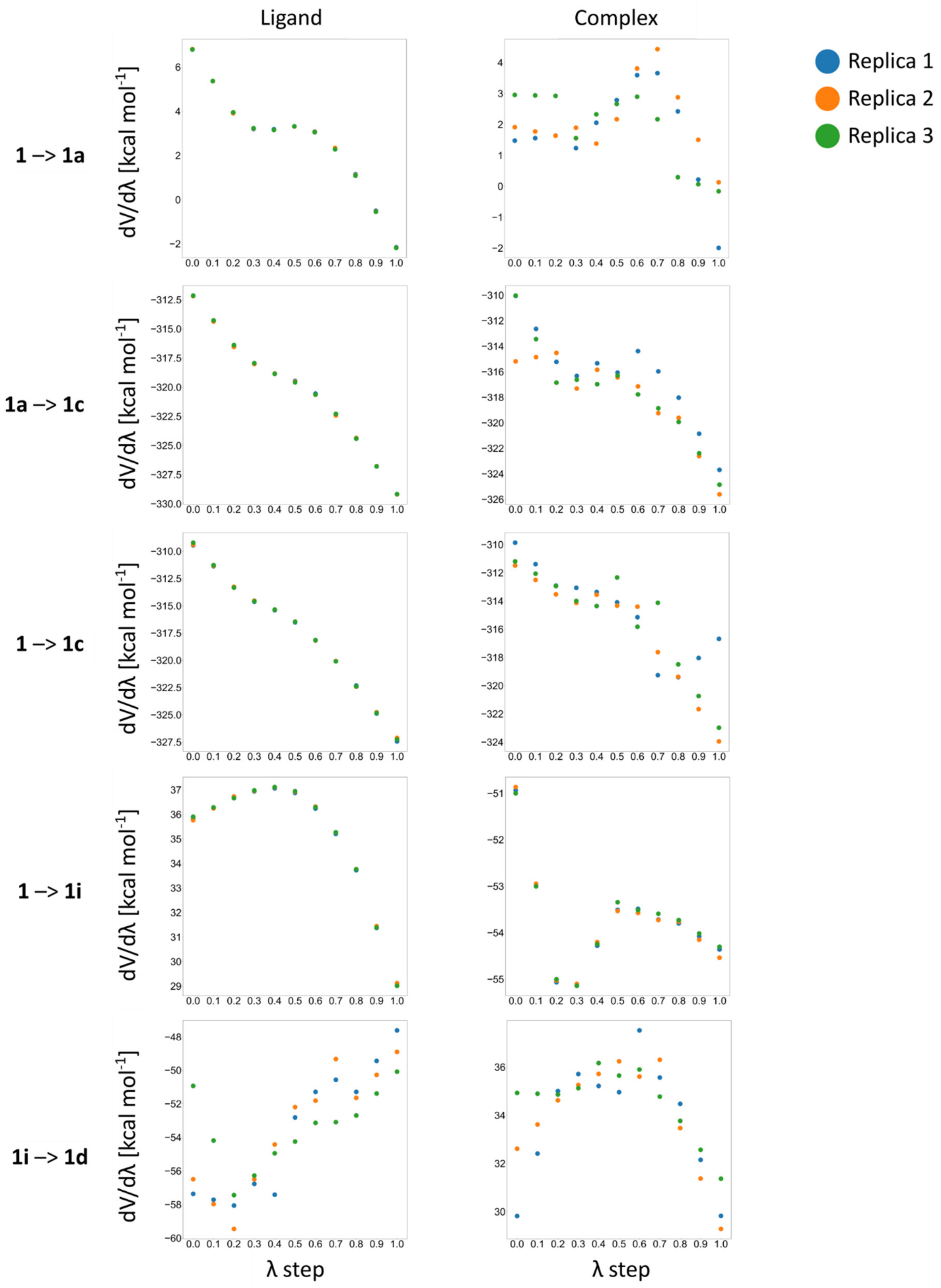
Ligand
Complex
Replica 1
Replica 2
Replica 3
1 –> 1a
1a –> 1c
1 –> 1c
1 –> 1i
1i –> 1d
dV/dλ [kcal mol-1]
λ step

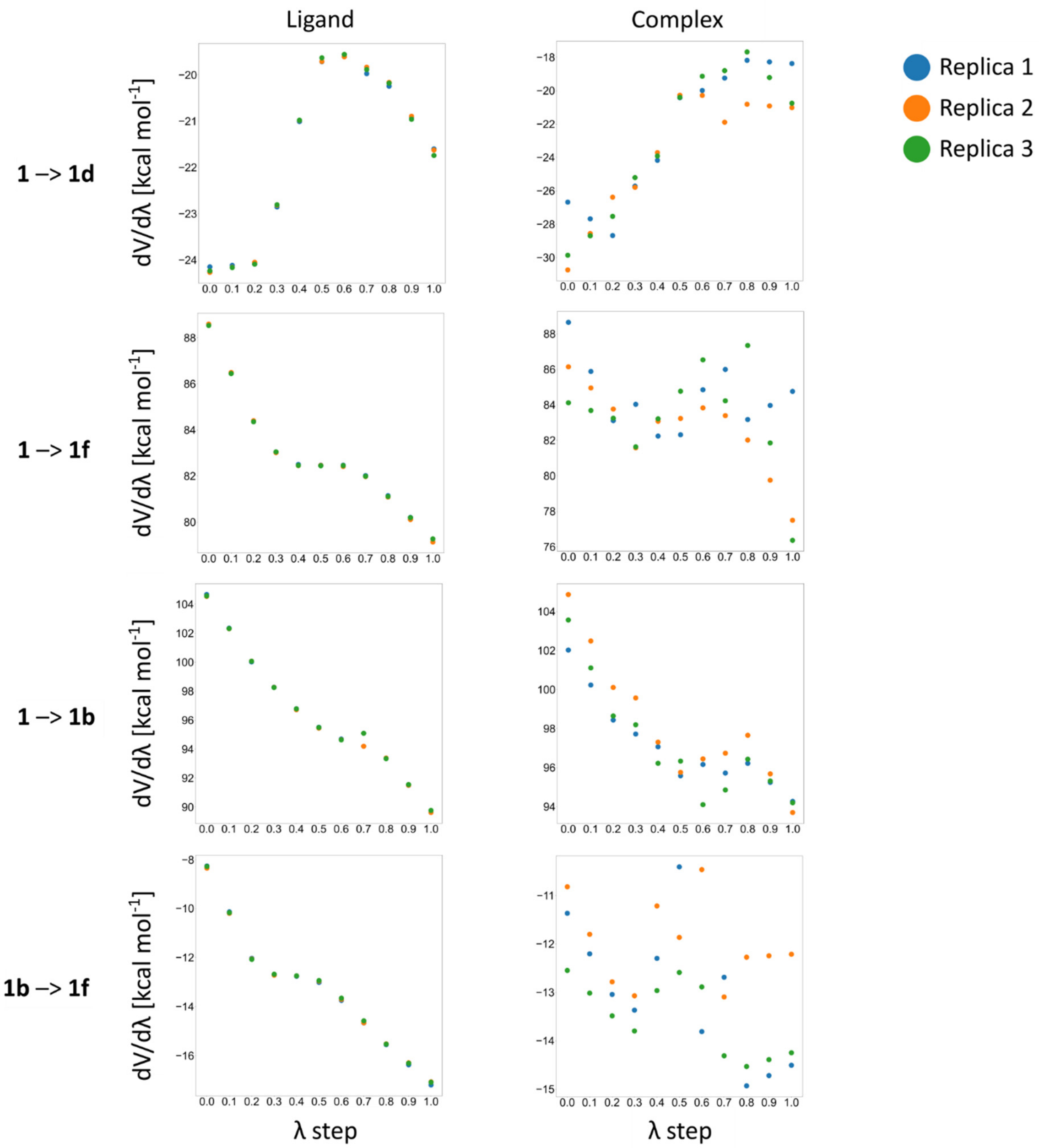

Figure S6. Ensemble-averaged dV/dλ after 10 ns per λ step (ligand in solvent) and 30 ns per λ step (complex in solvent) of sampling time for transitions of chlorflavonin derivatives. The standard error of the mean in all cases is < 0.1 kcal mol^{-1}.

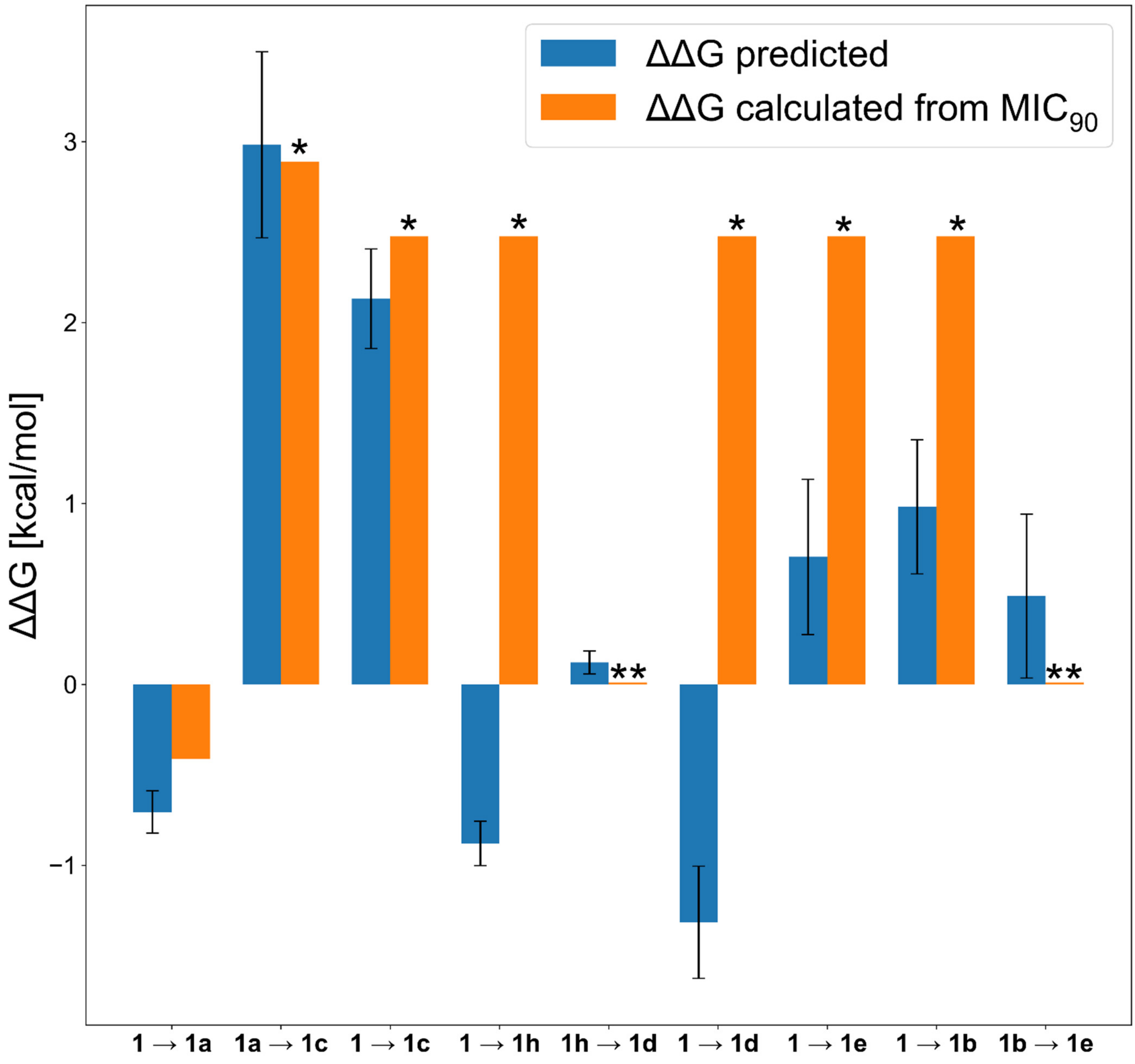

Figure S7. Comparison of predicted relative free energy of binding (ΔΔ*G* predicted) with the corresponding relative free energy of binding calculated from difference in minimal inhibitory concentration (ΔΔ*G* calculated from MIC_{90}) for the transitions V0 → V1 shown on the x-axis. The latter was estimated by $-RT \ln (MIC_{90}^{V0}/MIC_{90}^{V1})$ with $T = 300$ K, assuming that MIC_{90} correlates to the inhibitory constant of the enzyme. Error bars in ΔΔ*G* predicted denote the standard error of the mean (SEM); * higher than the value ($MIC_{90}^{V1} > 100$ µM), ** value uncertain (MIC_{90}^{V0} and $MIC_{90}^{V1} > 100$ µM).

5. Spectral Copies of ^{1}H-, ^{13}C- and ^{19}F-NMR Data

2-(3-chloro-2-hydroxyphenyl)-5-hydroxy-3,7,8-trimethoxy-4*H*-chromen-4-one (1)

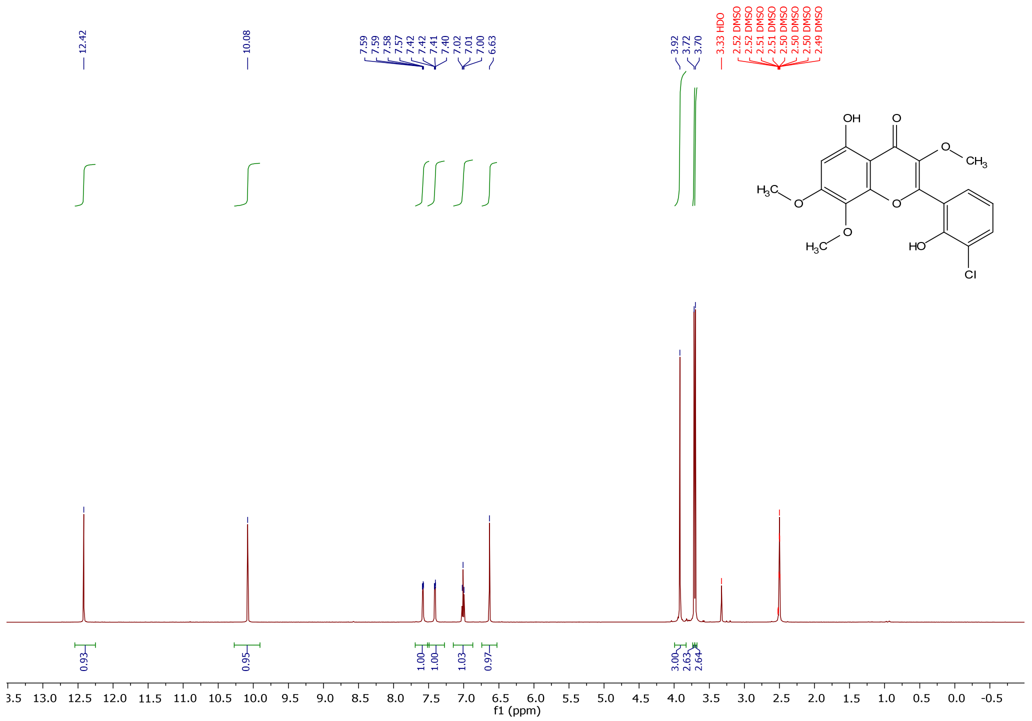

600 MHz, ^{1}H-NMR in DMSO-d_6

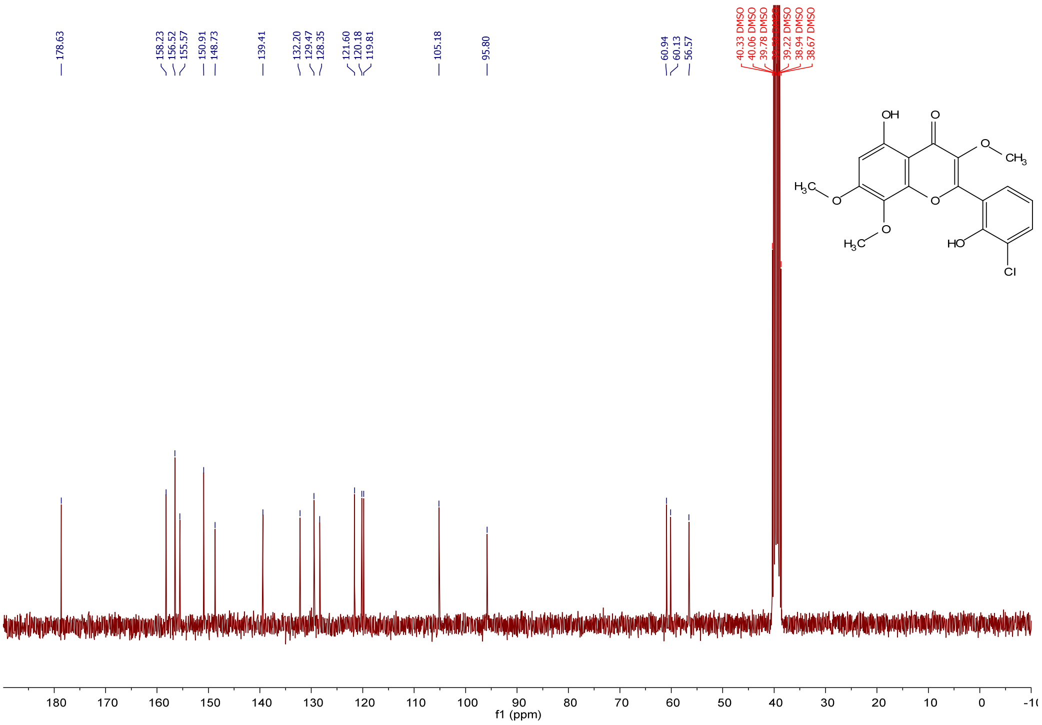

75 MHz, ^{13}C-NMR in DMSO-d_6

2-(3-bromo-2-hydroxyphenyl)-5-hydroxy-3,7,8-trimethoxy-4*H*-chromen-4-one (1a)

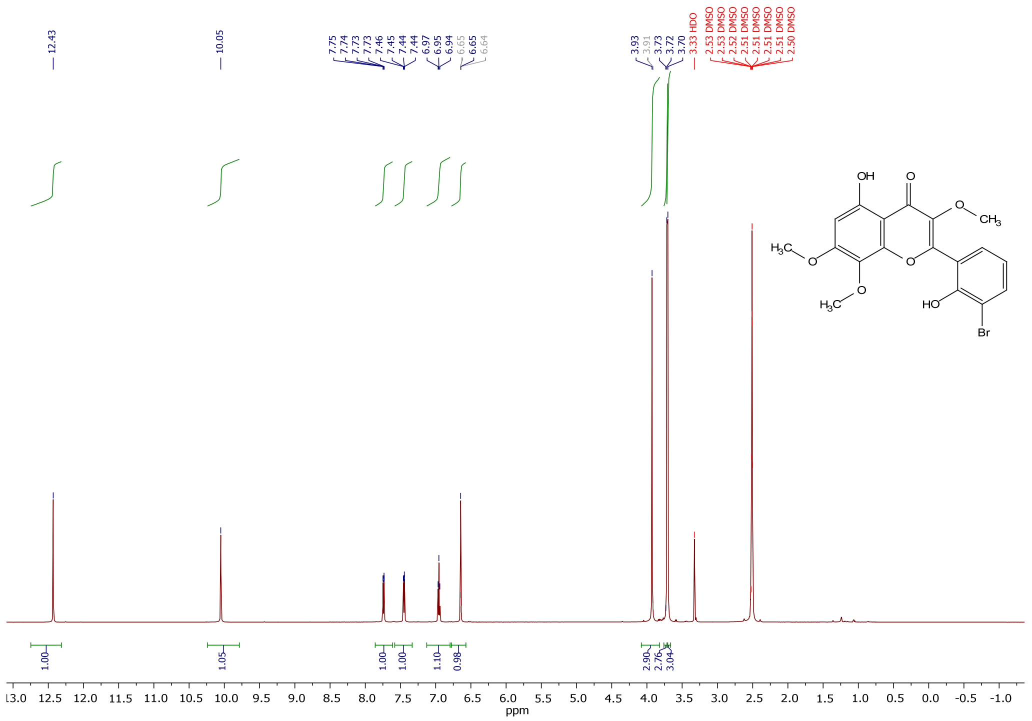

600 MHz, ^{1}H-NMR in DMSO-d_6

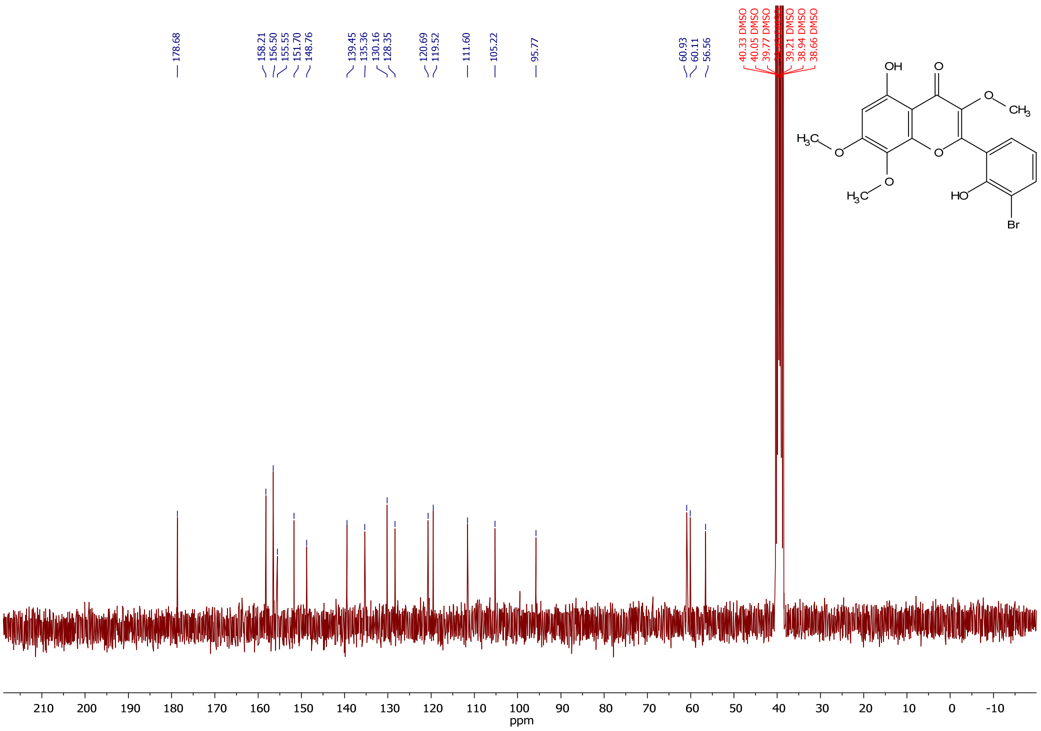

75 MHz, ^{13}C-NMR in DMSO-d_6

2-(3-fluoro-2-hydroxyphenyl)-5-hydroxy-3,7,8-trimethoxy-4*H*-chromen-4-one (1b)

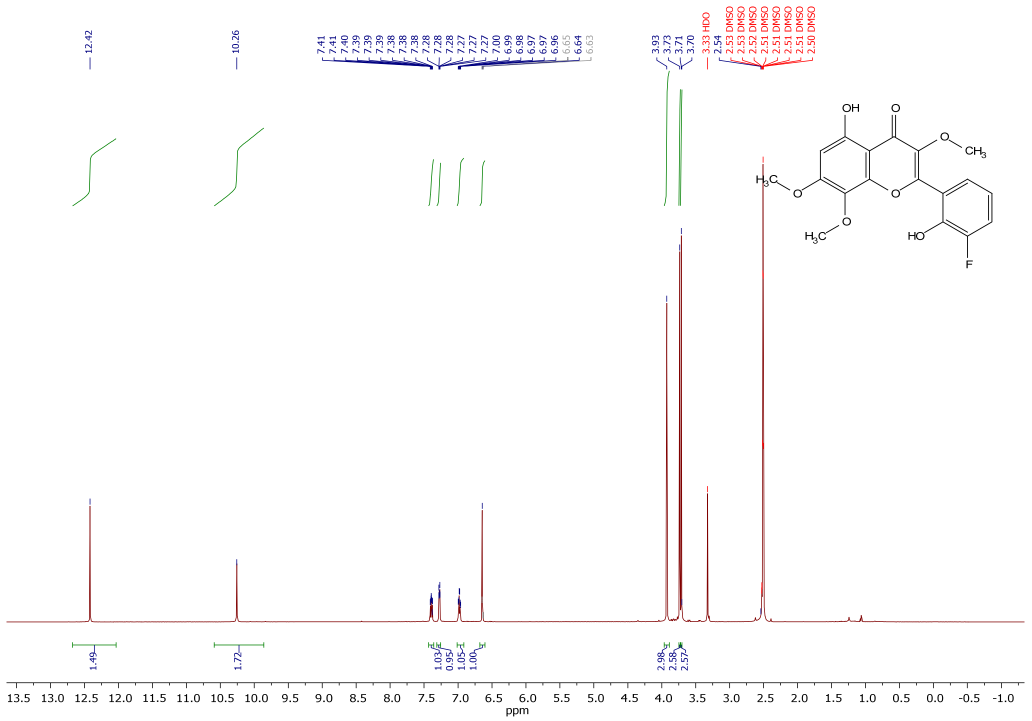

600 MHz, ^{1}H-NMR in DMSO-d_6

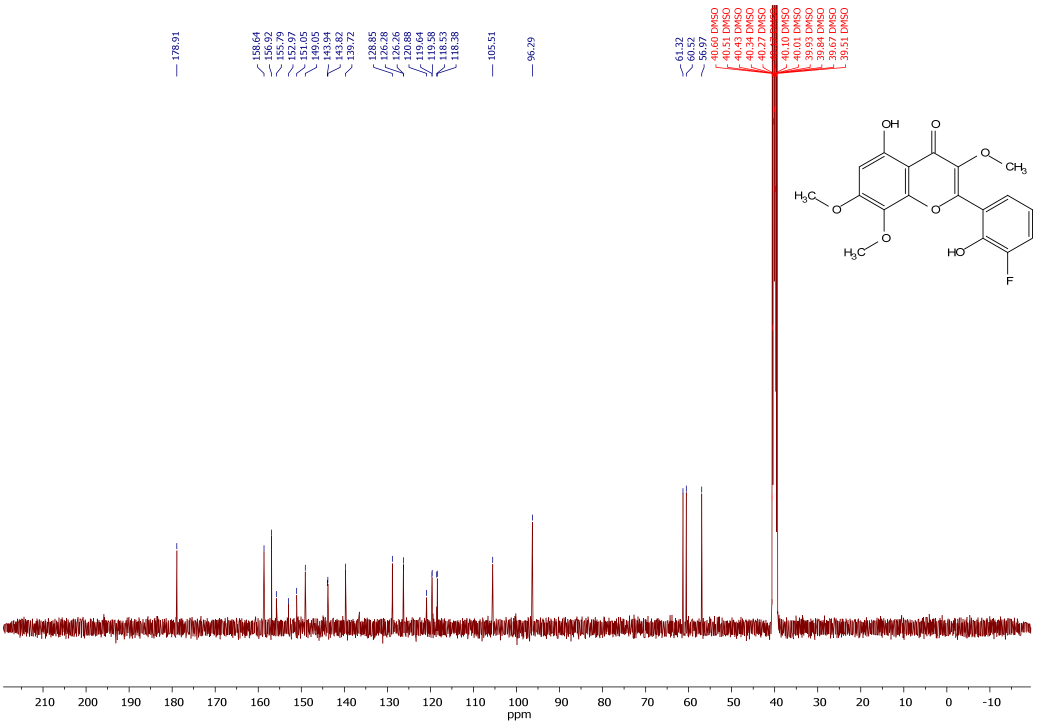

126 MHz, ^{13}C-NMR in DMSO-d_6

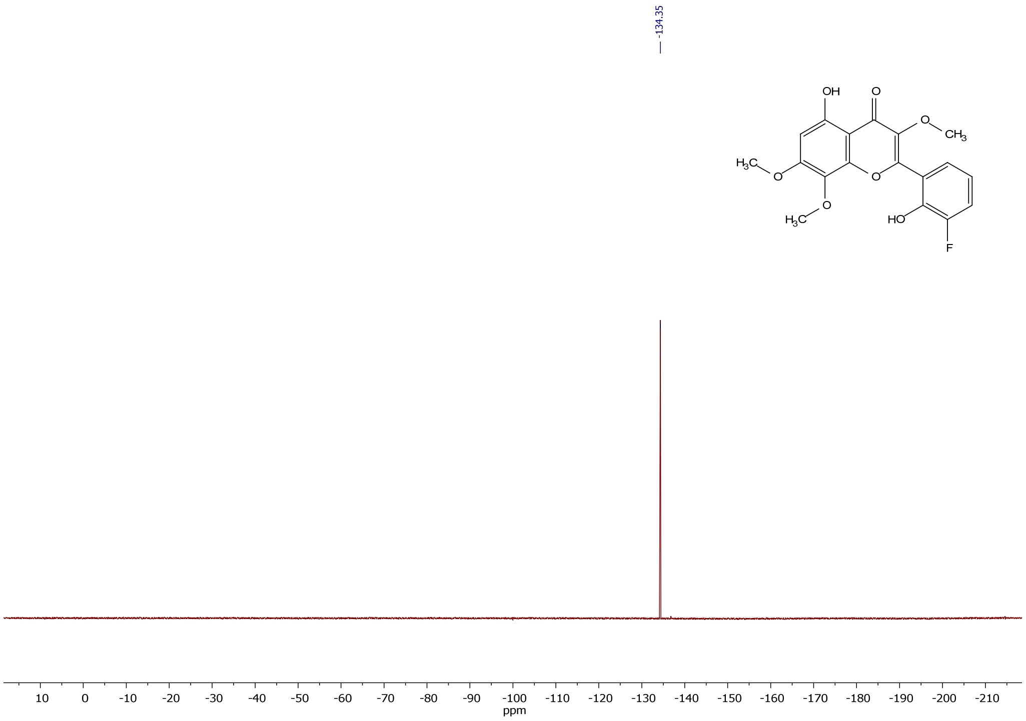

282 MHz, ^{19}F-NMR in DMSO-d_6

5-hydroxy-2-(2-hydroxy-3-iodophenyl)-3,7,8-trimethoxy-4*H*-chromen-4-one (1c)

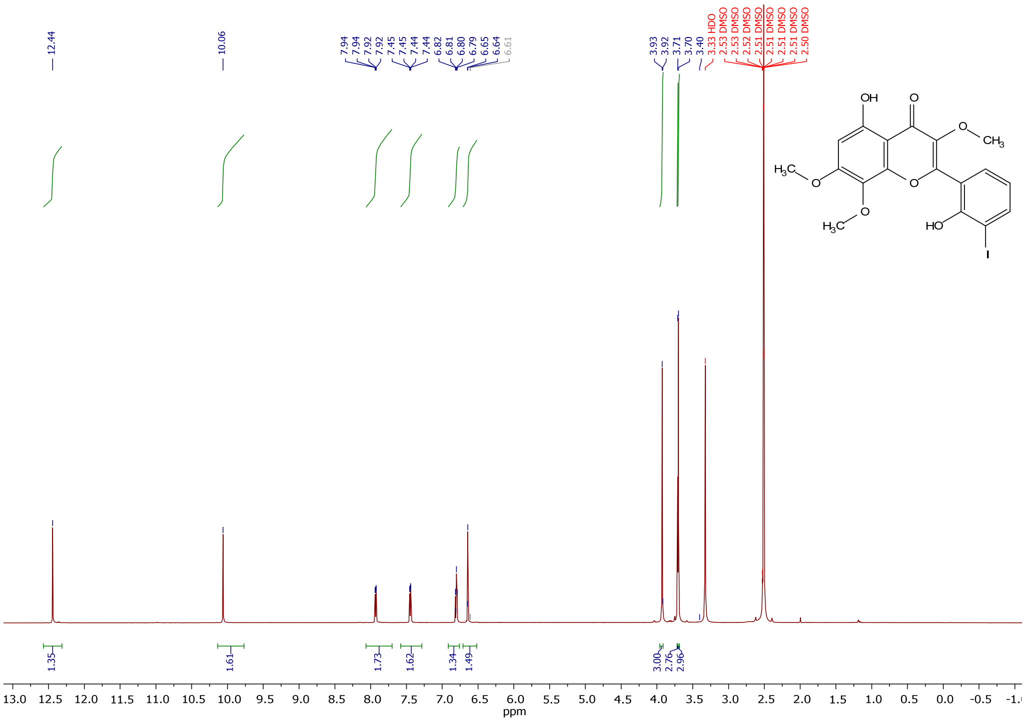

600 MHz, ^{1}H-NMR in DMSO-d_6

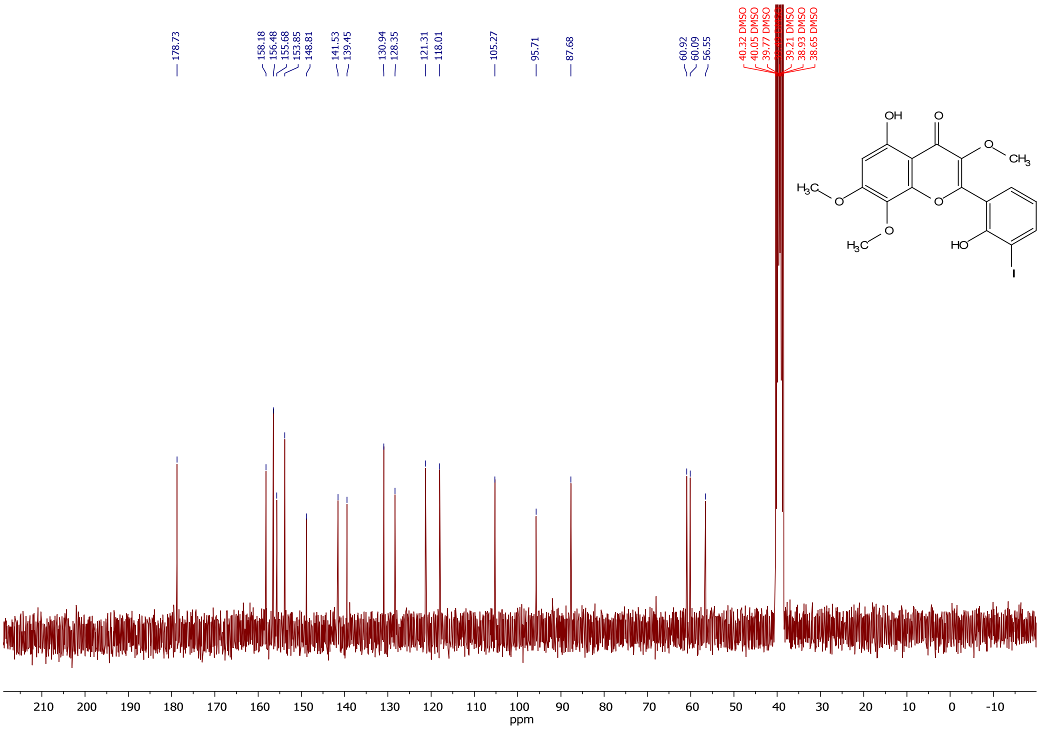

75 MHz, ^{13}C-NMR in DMSO-d_6

5-hydroxy-2-(2-hydroxy-3-(trifluoromethyl)phenyl)-3,7,8-trimethoxy-4*H*-chromen-4-one (1d)

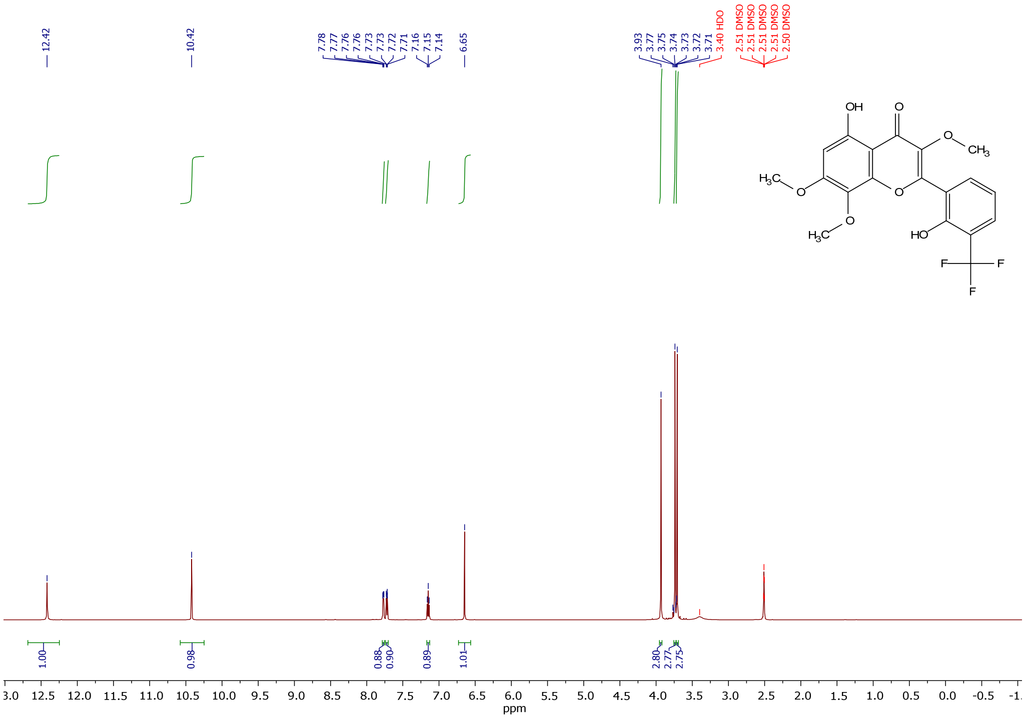

600 MHz, ^{1}H-NMR in DMSO-d_6

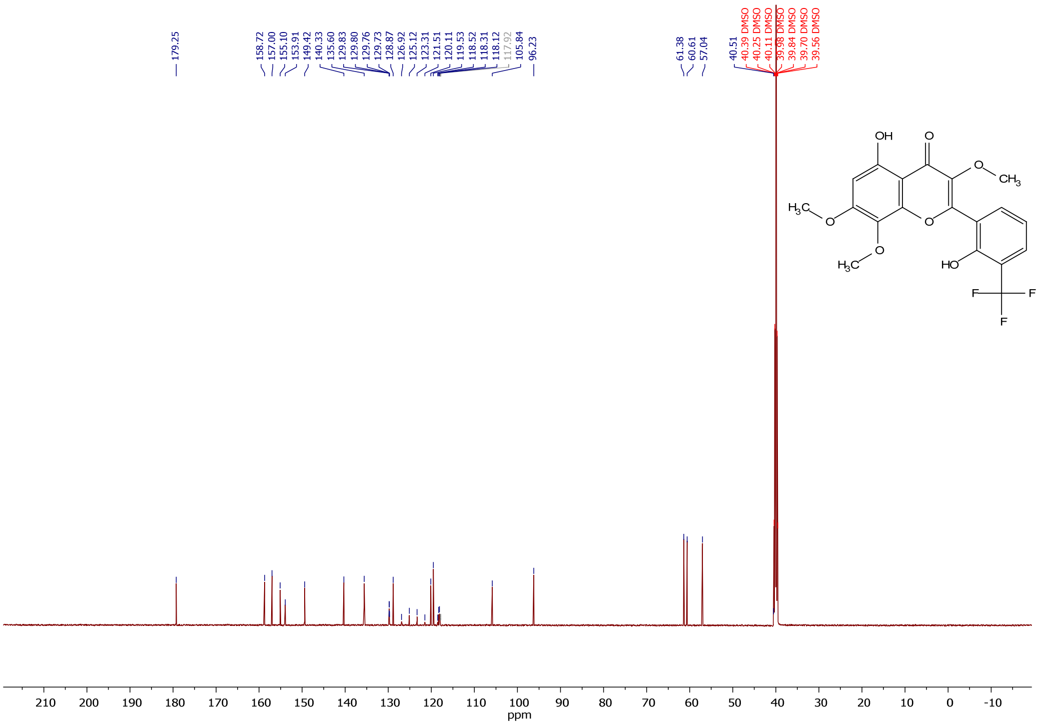

151 MHz, ^{13}C-NMR in DMSO-d_6

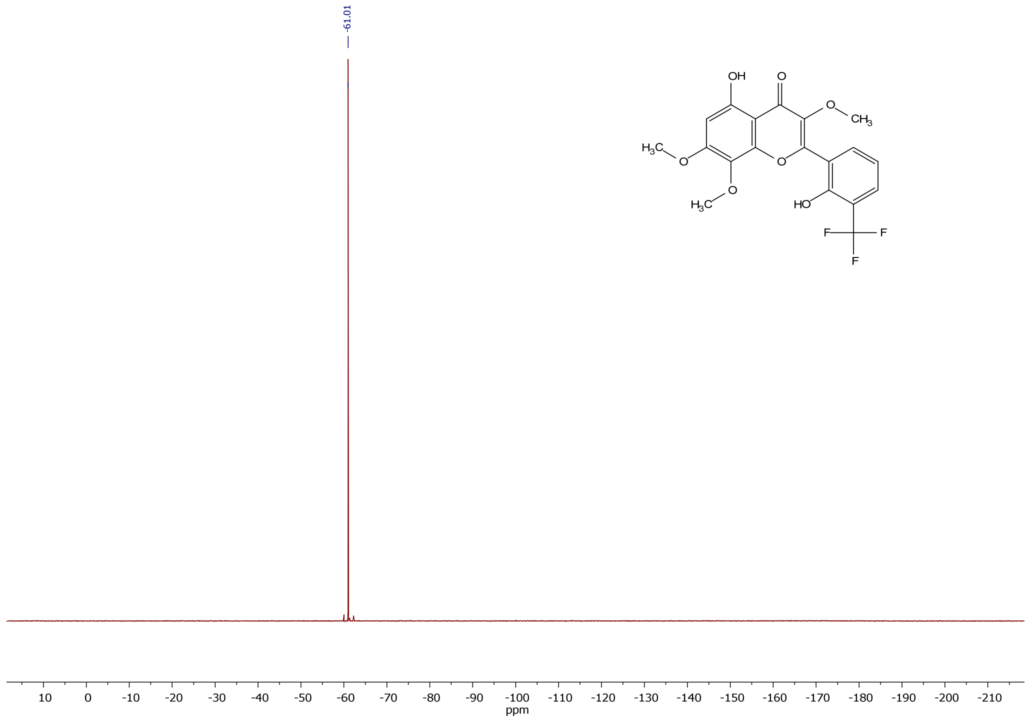

565 MHz, ^{19}F-NMR in DMSO-d_6

5-hydroxy-2-(2-hydroxy-3-methylphenyl)-3,7,8-trimethoxy-4*H*-chromen-4-one (1e)

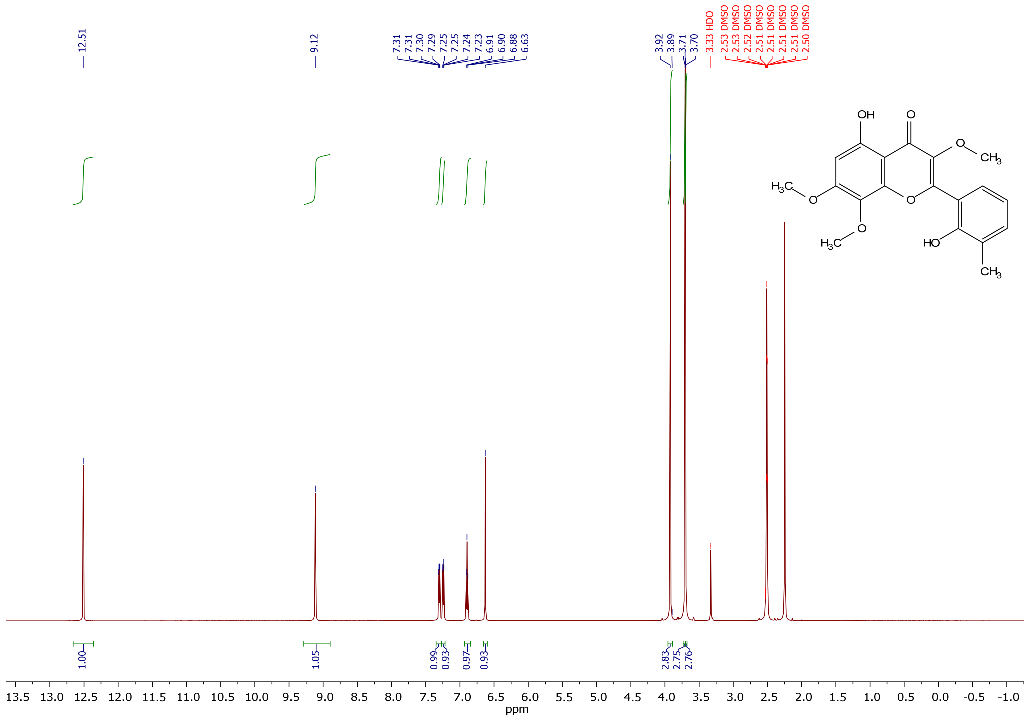

600 MHz, ^{1}H-NMR in DMSO-d_6

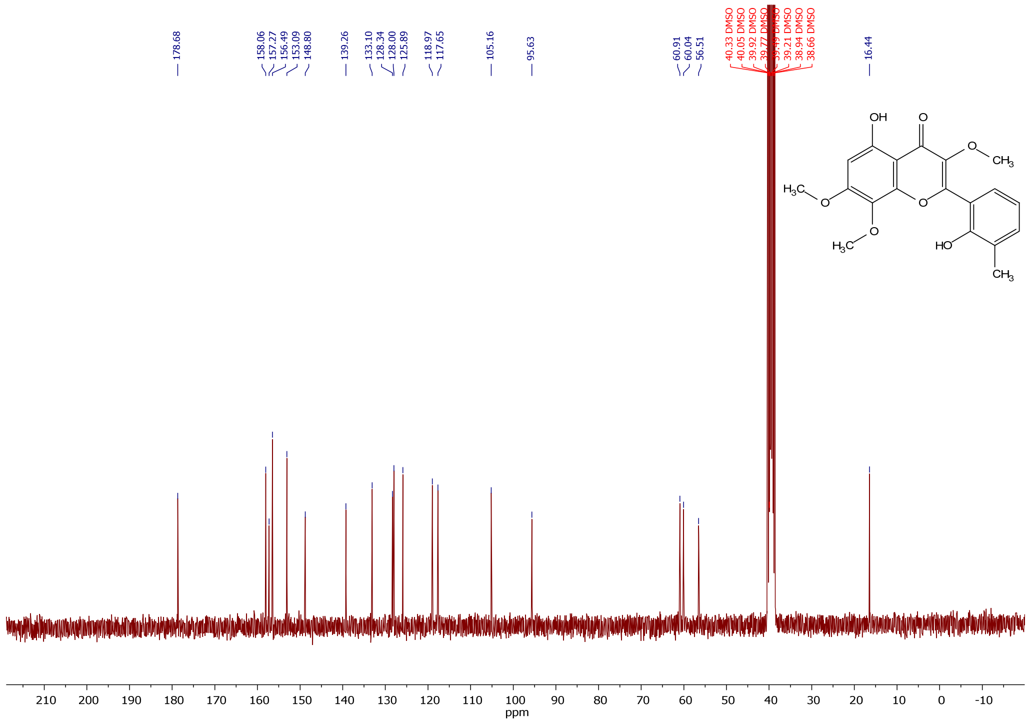

75 MHz, ^{13}C-NMR in DMSO-d_6

2-(3-bromo-2-fluorophenyl)-5-hydroxy-3,7,8-trimethoxy-*4H*-chromen-4-one (1f)

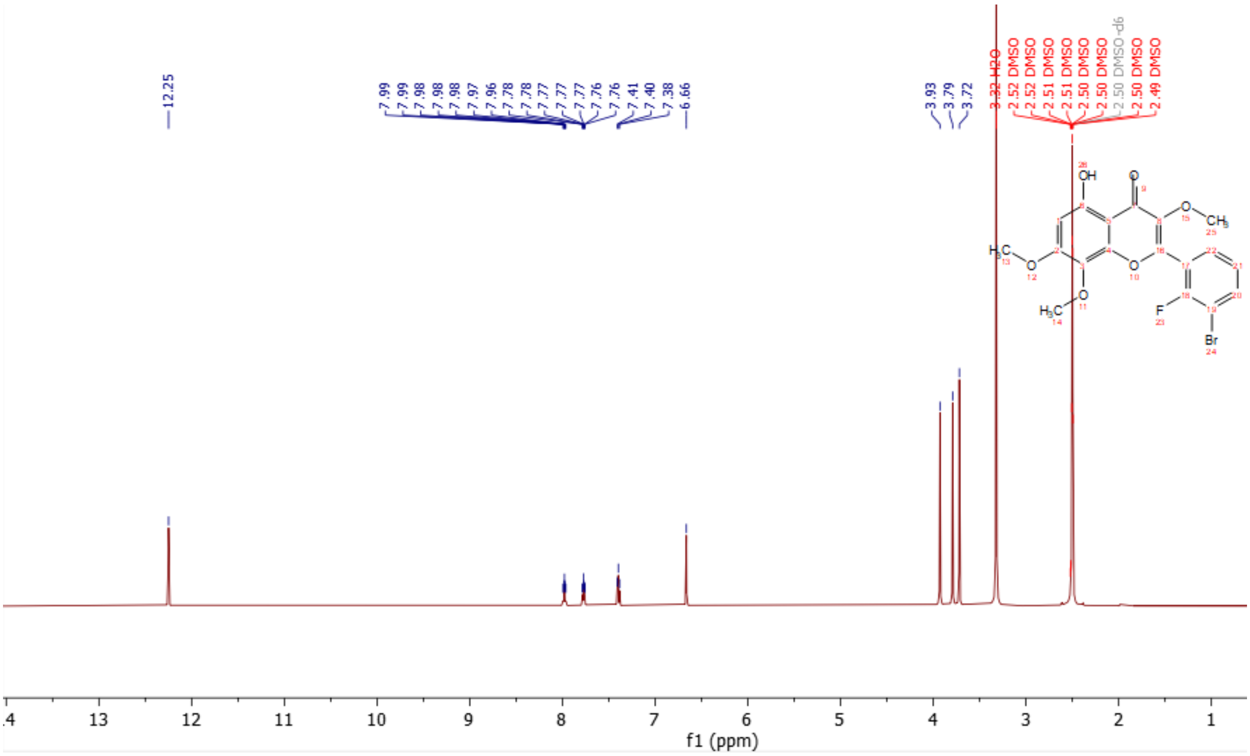

600 MHz, ^{1}H-NMR in DMSO-d_6

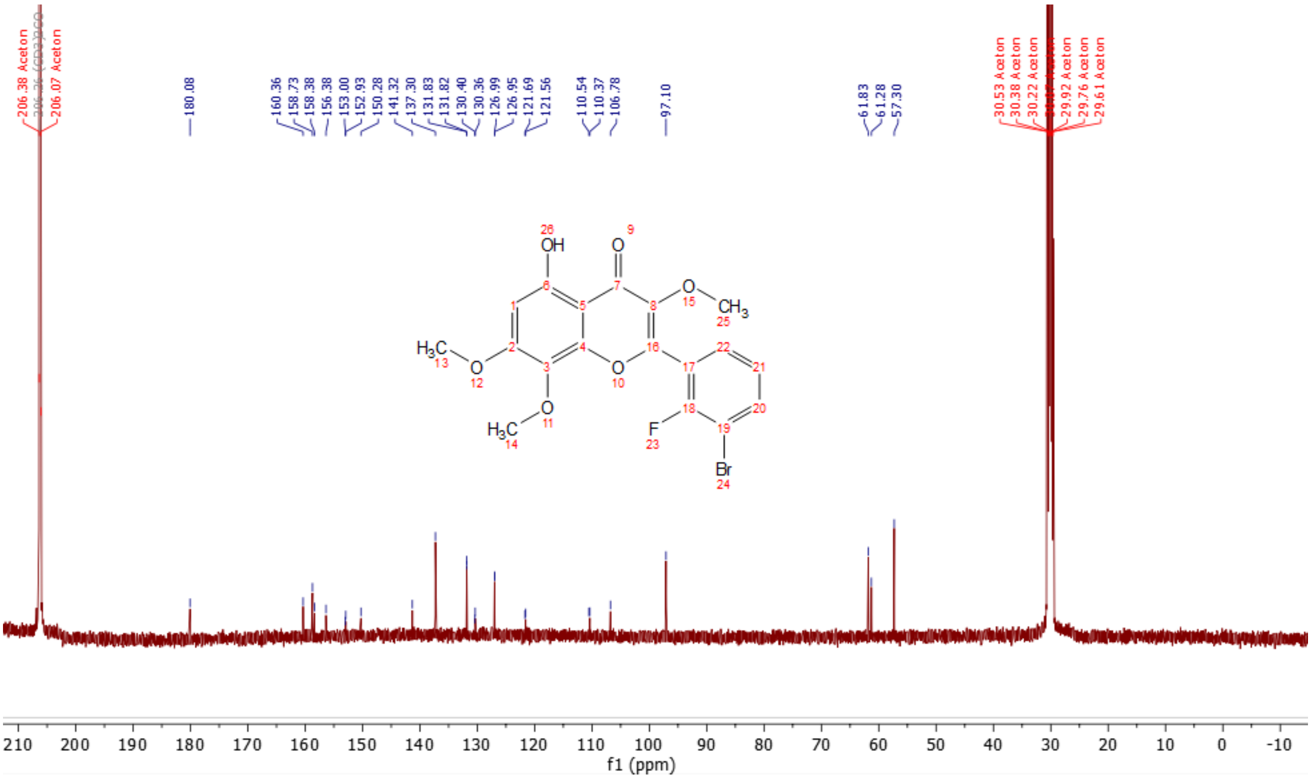

126 MHz, ^{13}C-NMR in Acetone-d_6

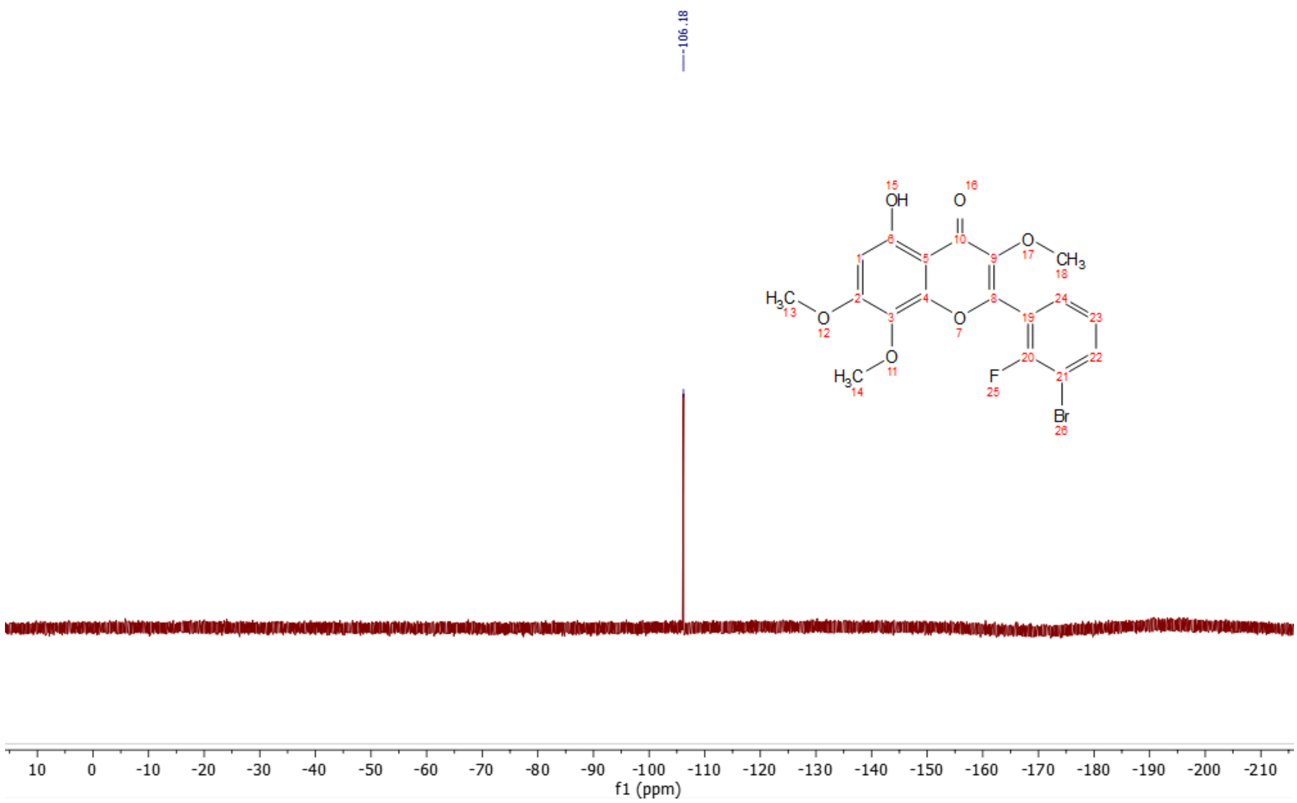

565 MHz, ^{19}F-NMR in Acetone-d_6

2-(3-bromo-2-(difluoromethoxy)phenyl)-5-hydroxy-3,7,8-trimethoxy-*4H*-chromen-4-one (1g)

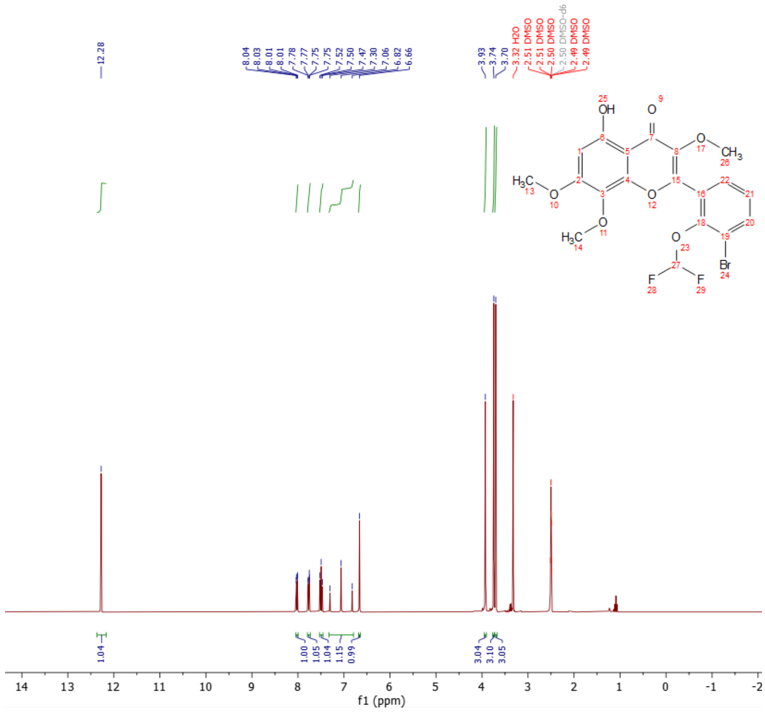

300 MHz, ^{1}H-NMR in DMSO-d_6

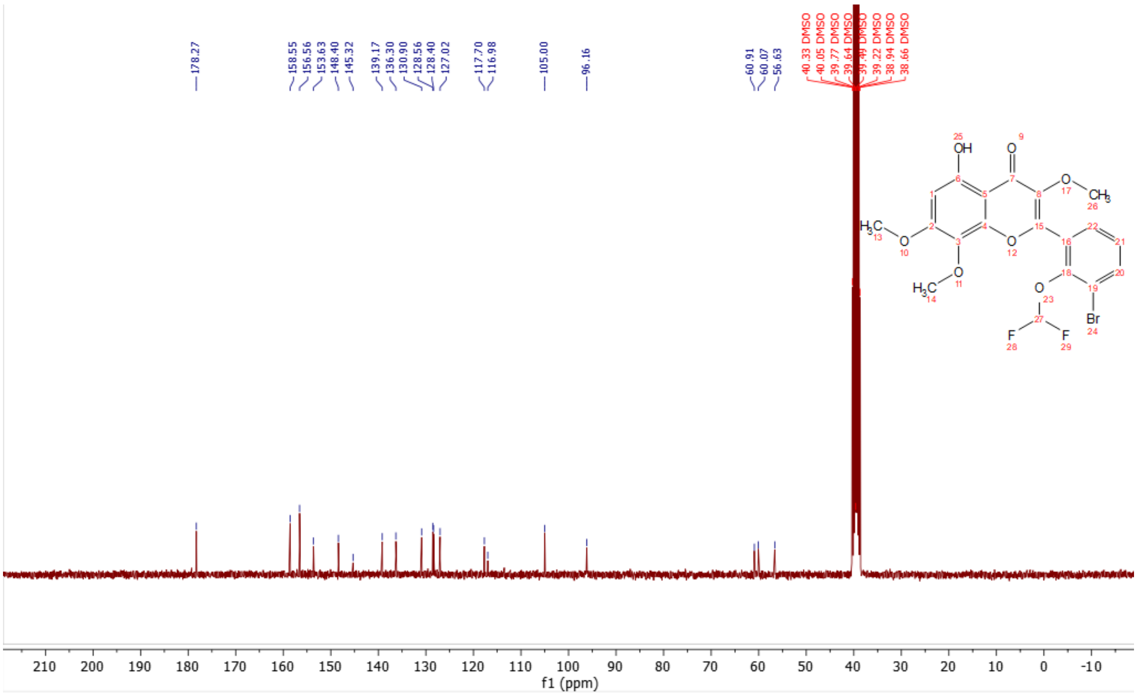

75 MHz, ^{13}C-NMR in DMSO-d_6

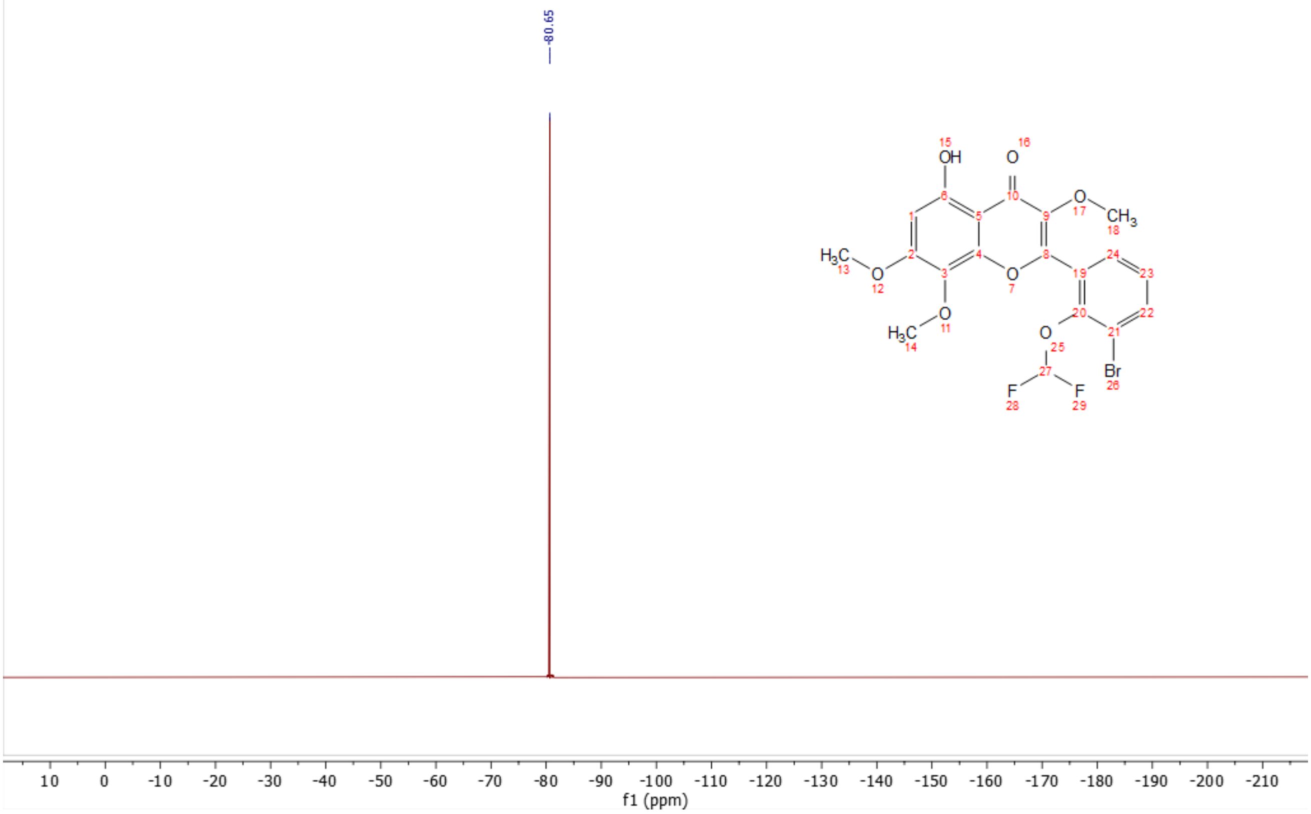

282 MHz, ^{19}F-NMR in DMSO-d_6

2-(3-chloro-5-fluoro-2-hydroxyphenyl)-5-hydroxy-3,7,8-trimethoxy-4*H*-chromen-4-one (1h)

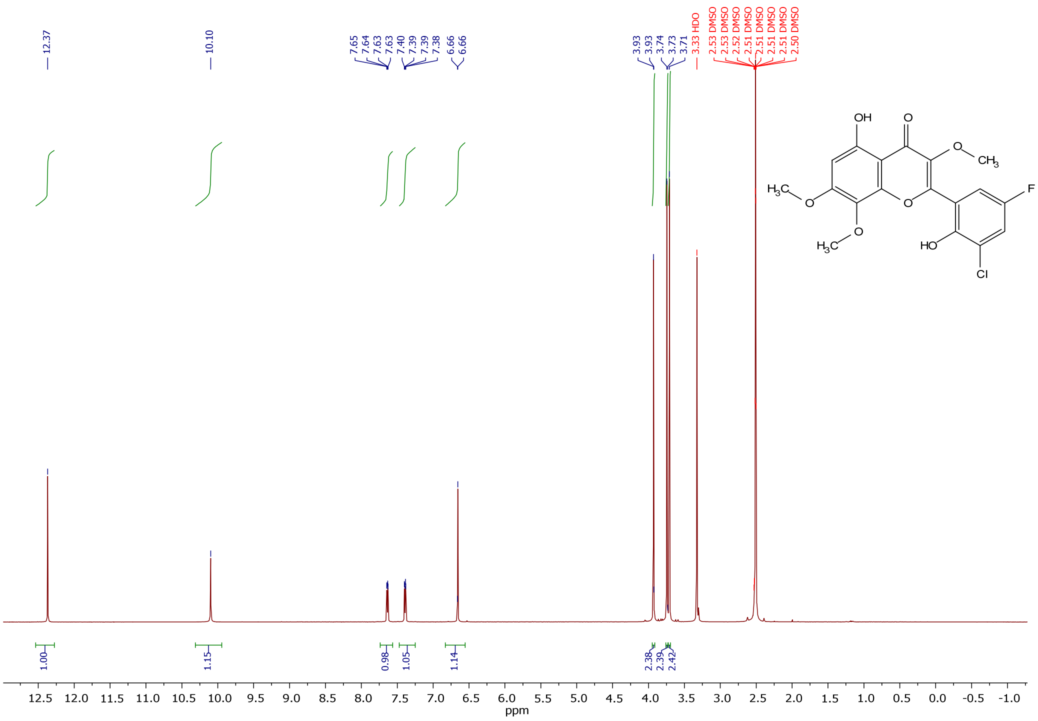

600 MHz, ^{1}H-NMR in DMSO-d_6

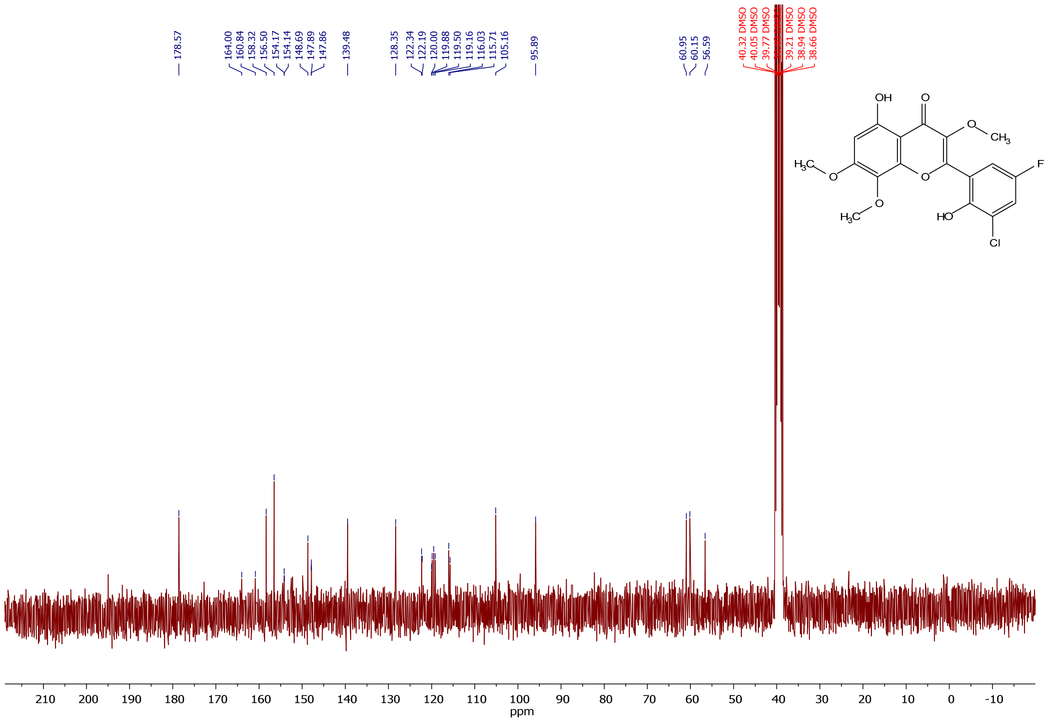

75 MHz, ^{13}C-NMR in DMSO-d_6

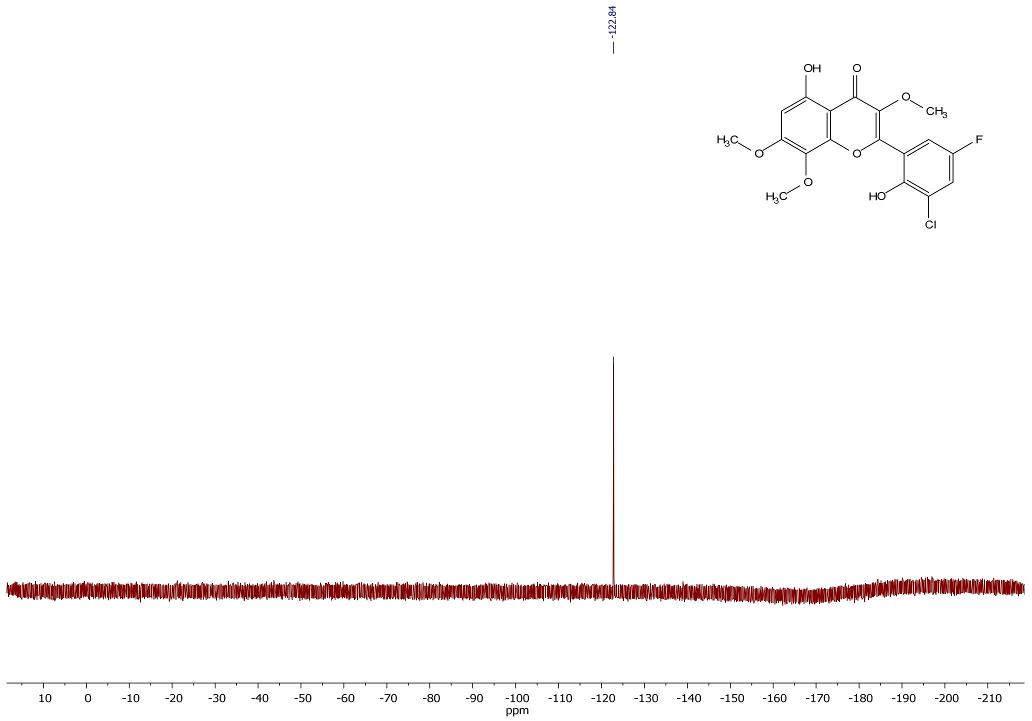

565 MHz, ^{19}F-NMR in DMSO-d_6

2-(3-bromo-5-fluoro-2-hydroxyphenyl)-5-hydroxy-3,7,8-trimethoxy-4*H*-chromen-4-one (1i)

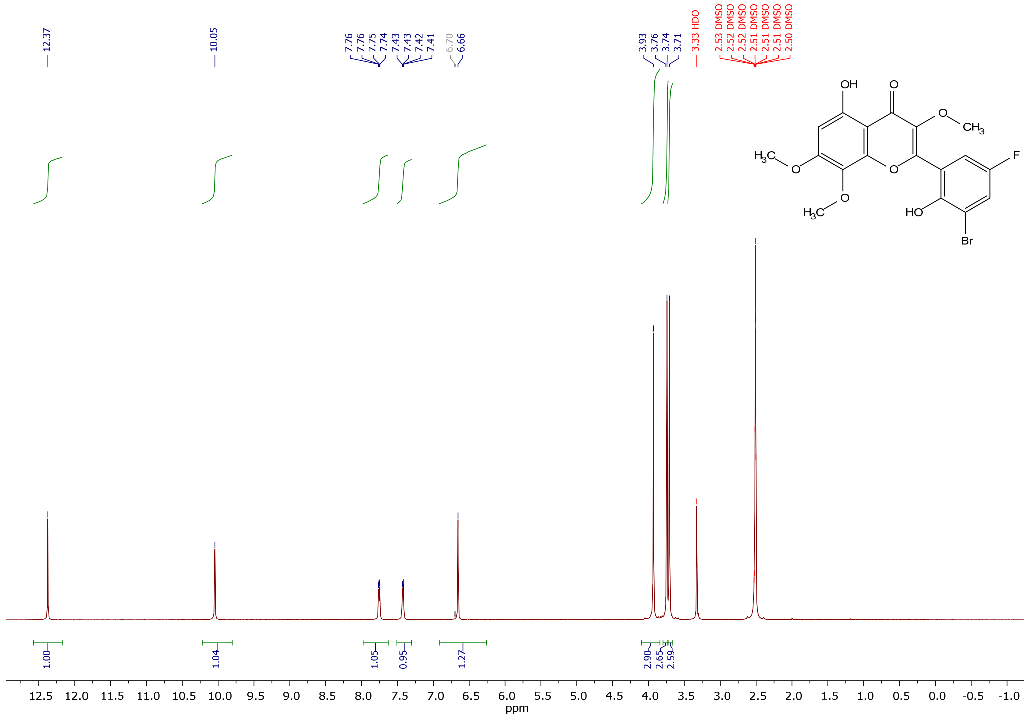

600 MHz, ^{1}H-NMR in DMSO-d_6

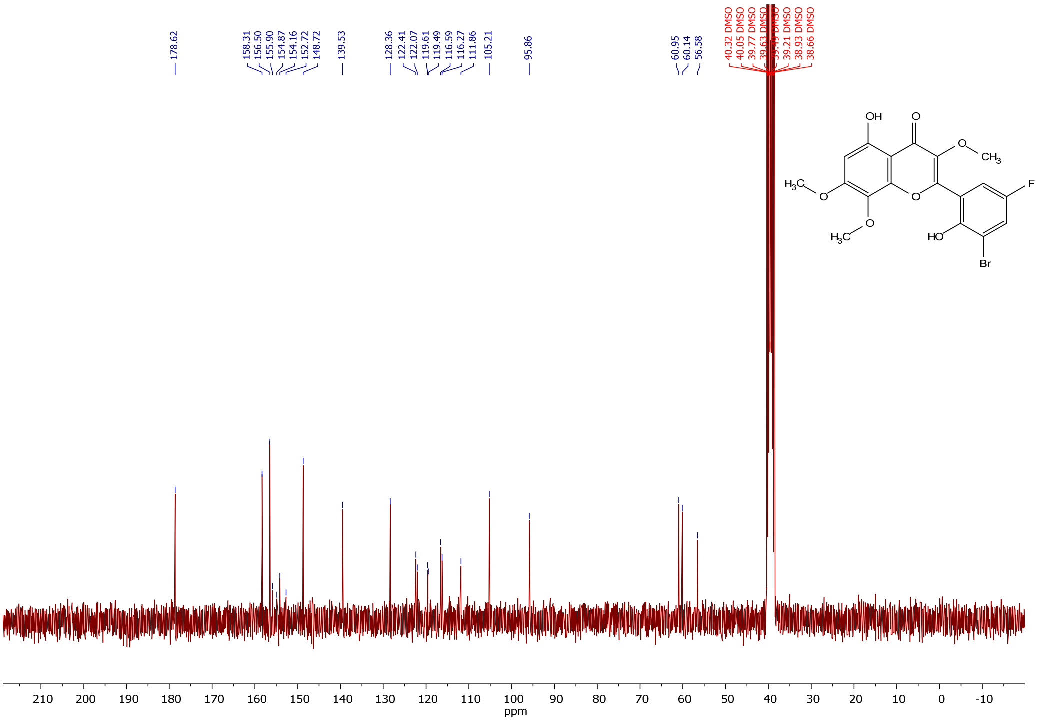

75 MHz, ^{13}C-NMR in DMSO-d_6

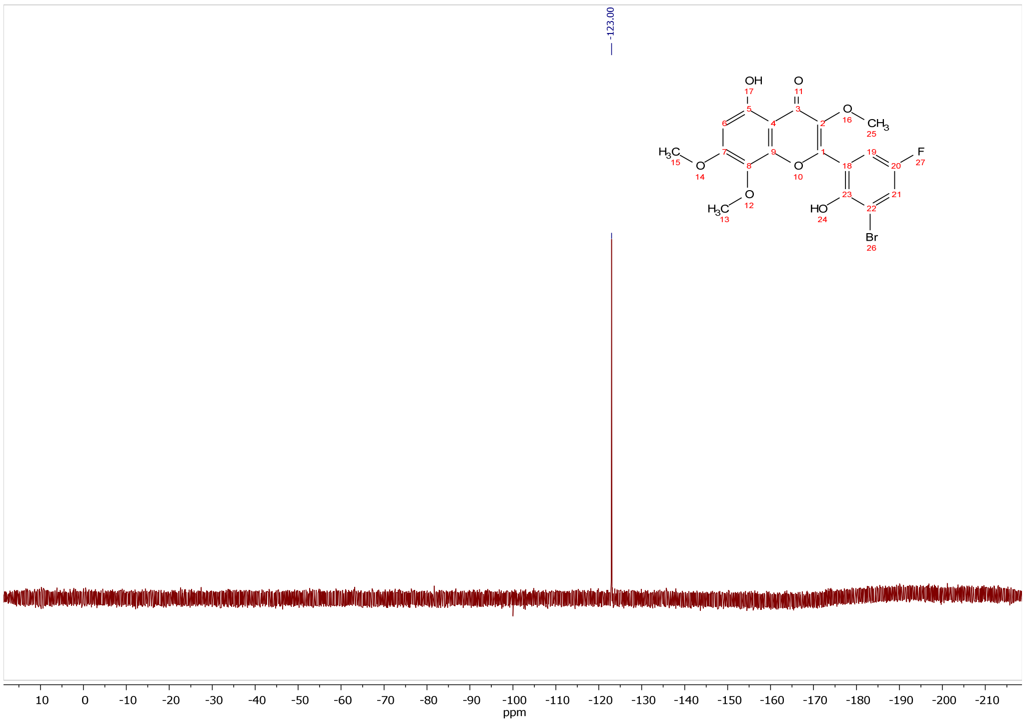

565 MHz, ^{19}F-NMR in DMSO-d_6

2-(3-bromo-2-hydroxy-5-methylphenyl)-5-hydroxy-3,7,8-trimethoxy-4H-chromen-4-one (1j)

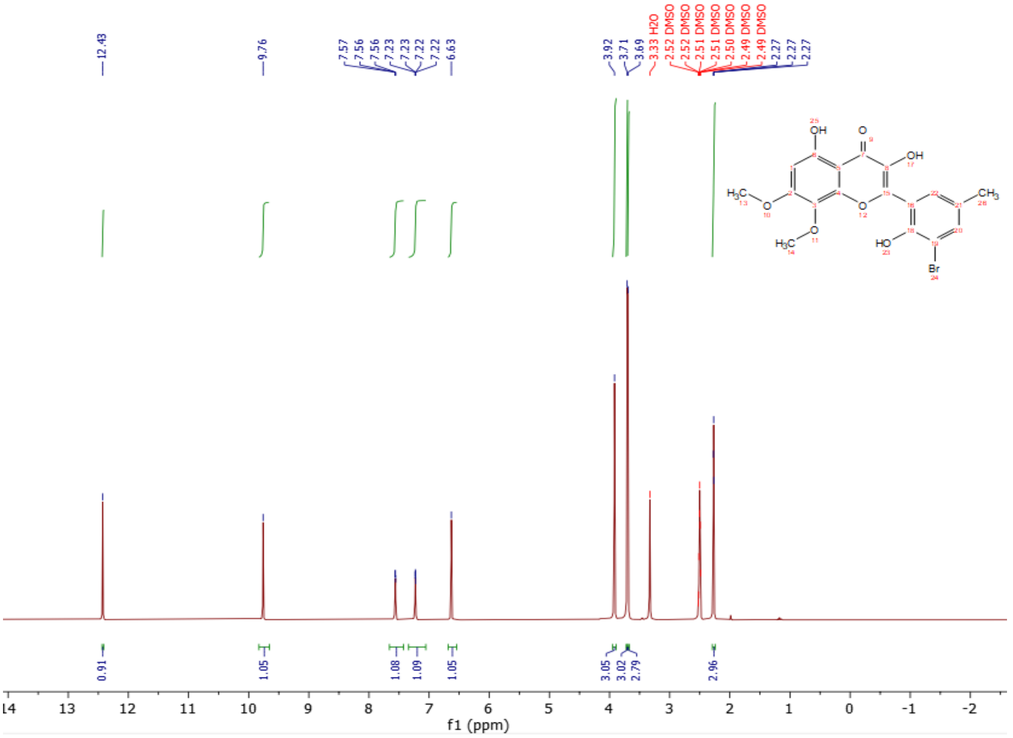

300 MHz, ^{1}H-NMR in DMSO-d_6

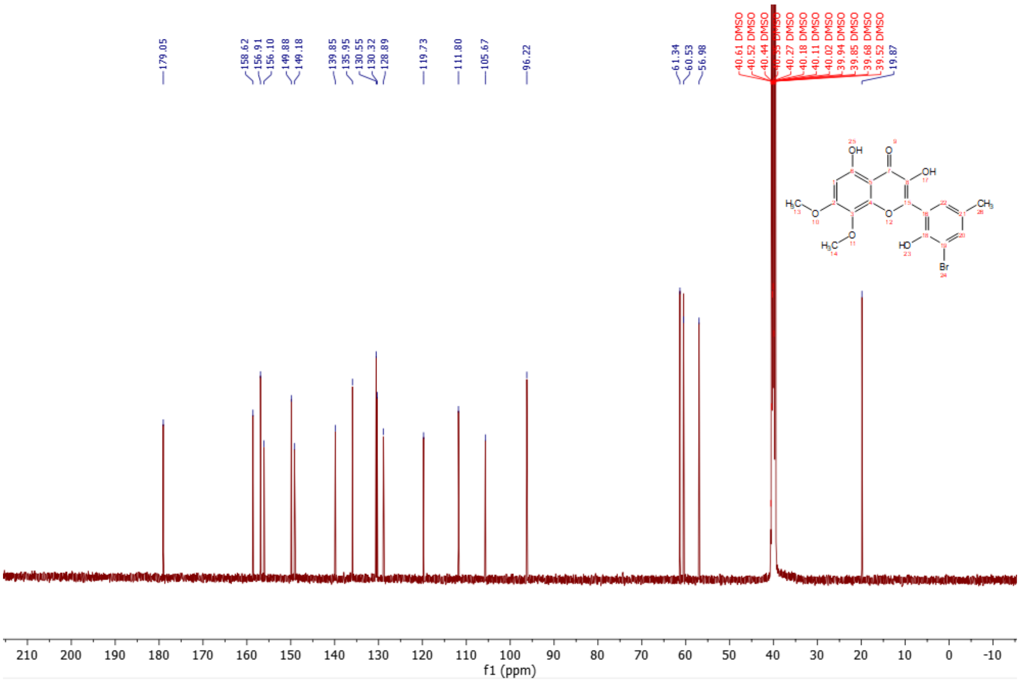

126 MHz, ^{13}C-NMR in DMSO-d_6

2-(4-chloro-2-hydroxyphenyl)-5-hydroxy-3,7,8-trimethoxy-4*H*-chromen-4-one (1k)

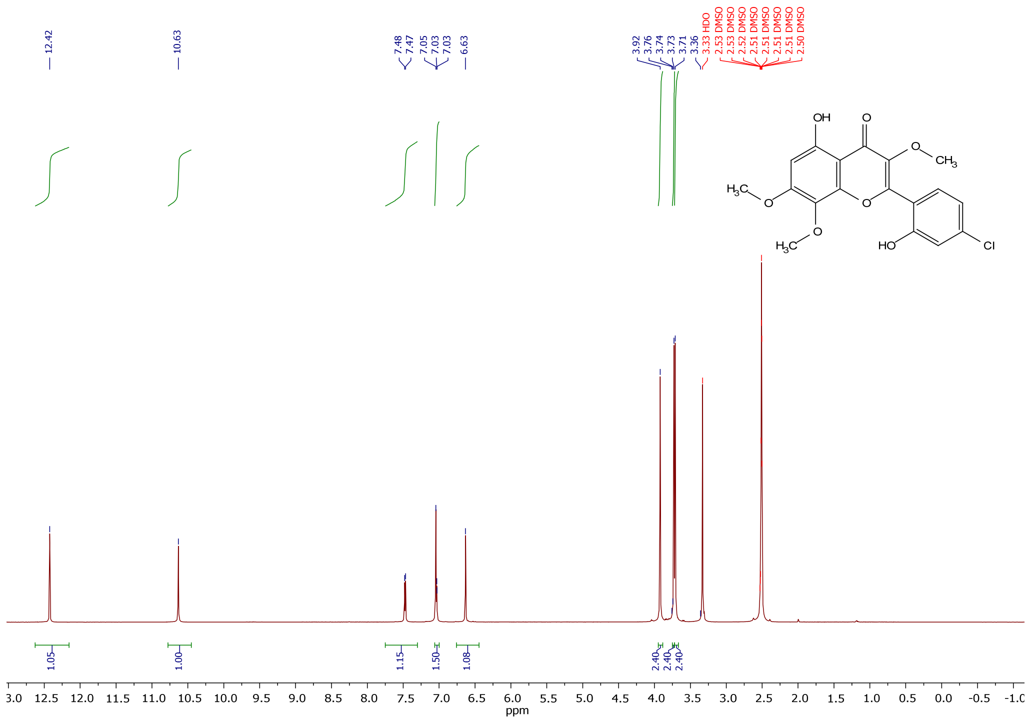

600 MHz, ^{1}H-NMR in DMSO-d_6

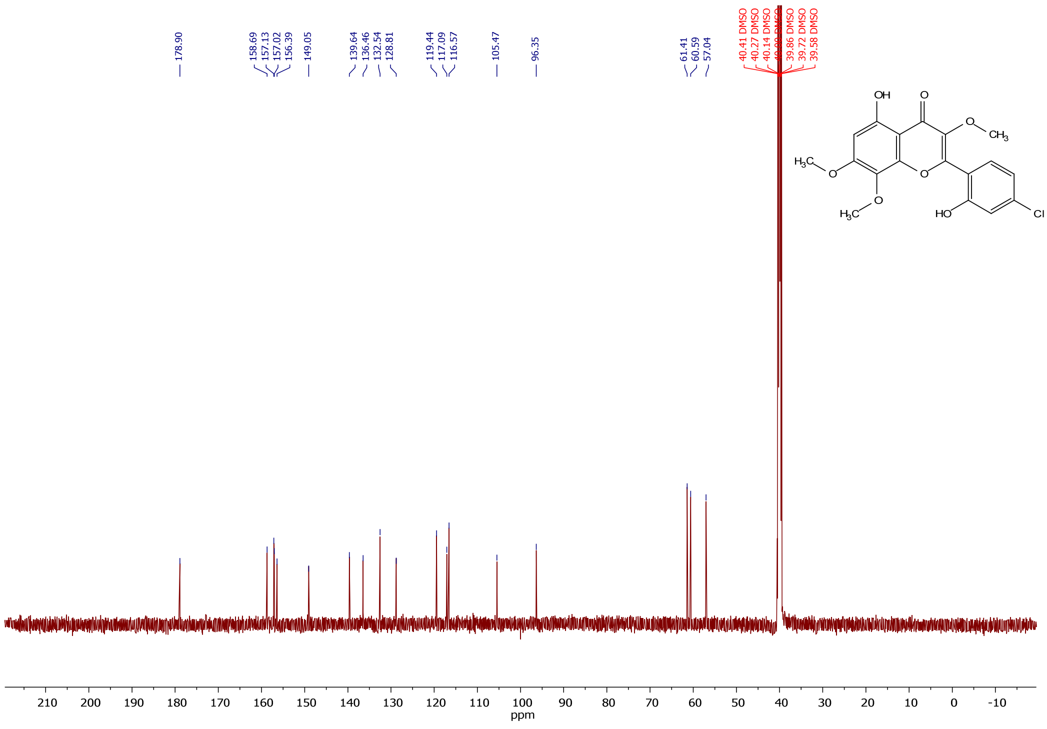

151 MHz, ^{13}C-NMR in DMSO-d_6

2-(4-chloro-3-hydroxyphenyl)-5-hydroxy-3,7,8-trimethoxy-4*H*-chromen-4-one (1l)

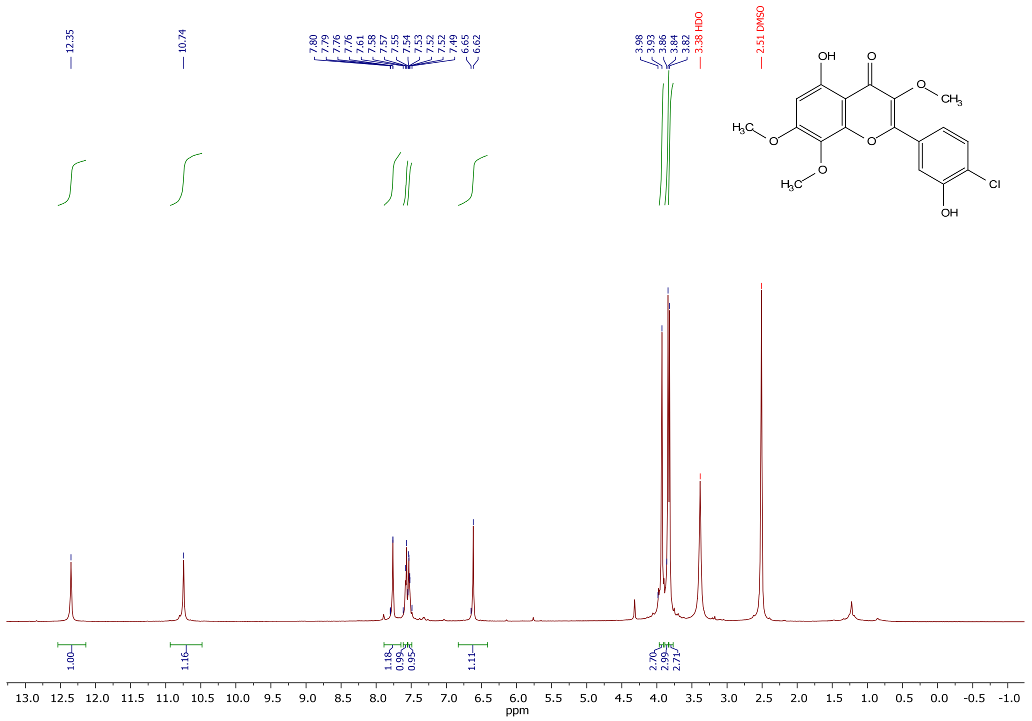

600 MHz, ^{1}H-NMR in DMSO-d_6

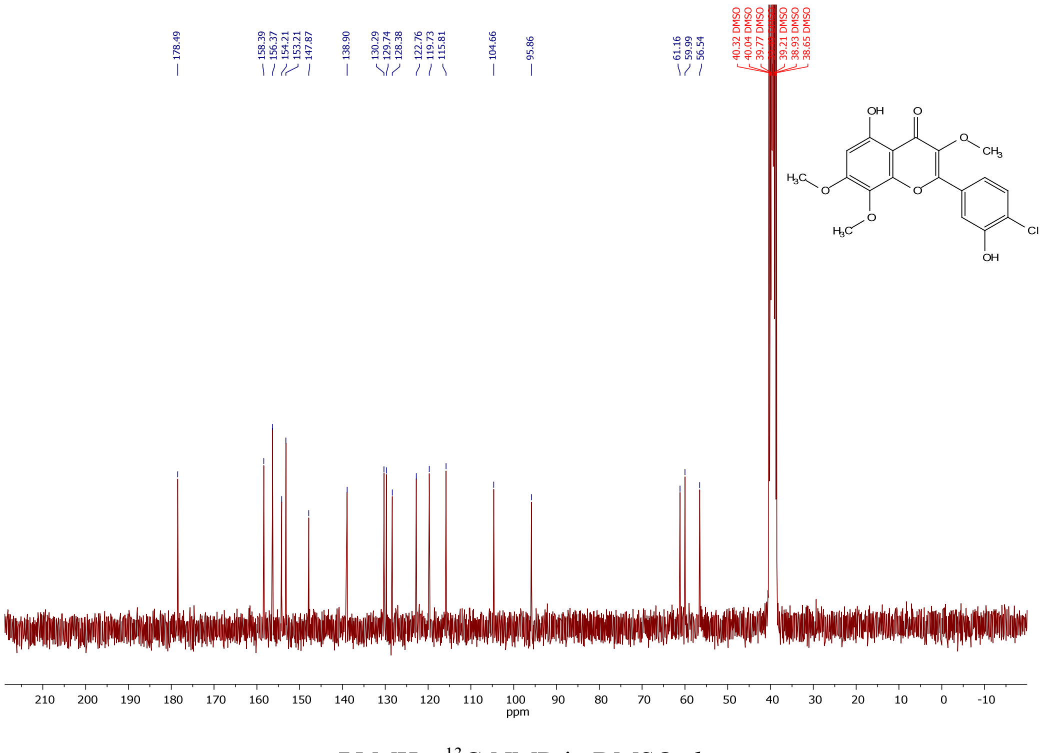

75 MHz, ^{13}C-NMR in DMSO-d_6

2-(2,3-dichlorophenyl)-5-hydroxy-3,7,8-trimethoxy-4*H*-chromen-4-one (1m)

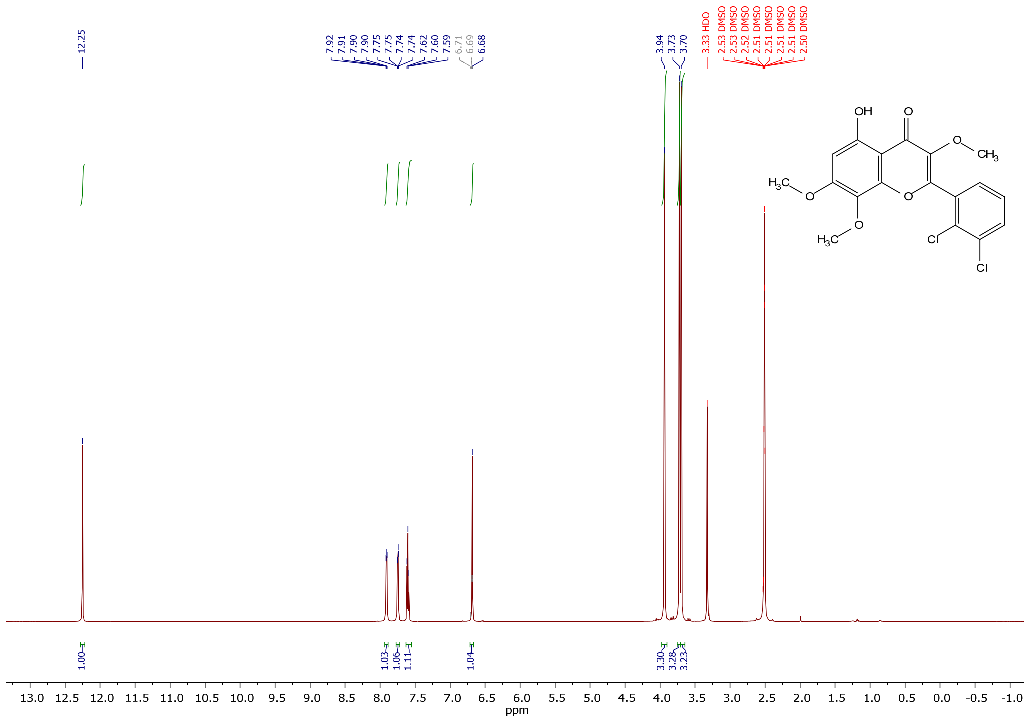

600 MHz, ^{1}H-NMR in DMSO-d_6

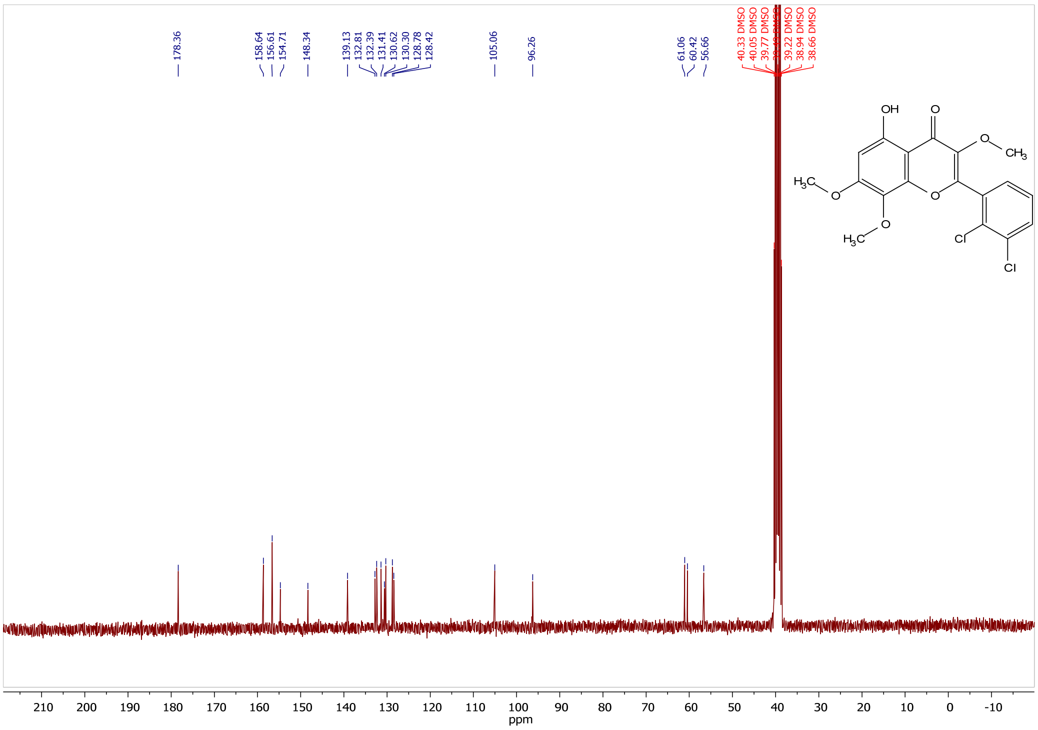

75 MHz, ^{13}C-NMR in DMSO-d_6

2-(3-chlorophenyl)-5-hydroxy-3,7,8-trimethoxy-4*H*-chromen-4-one (1o)

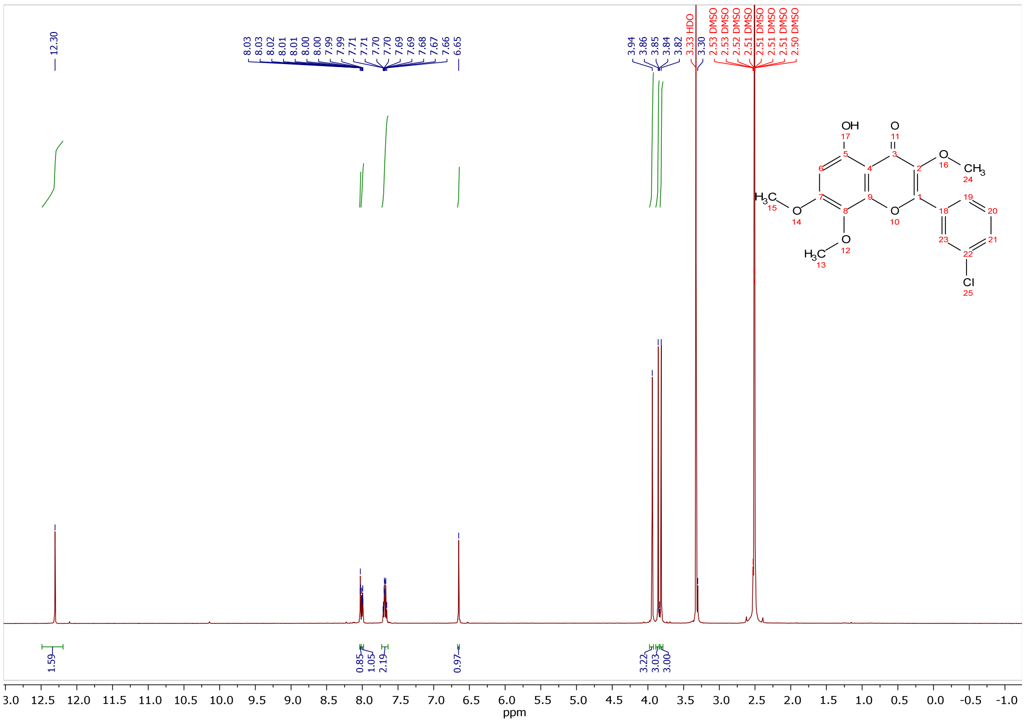

600 MHz, ^{1}H-NMR in DMSO-d_6

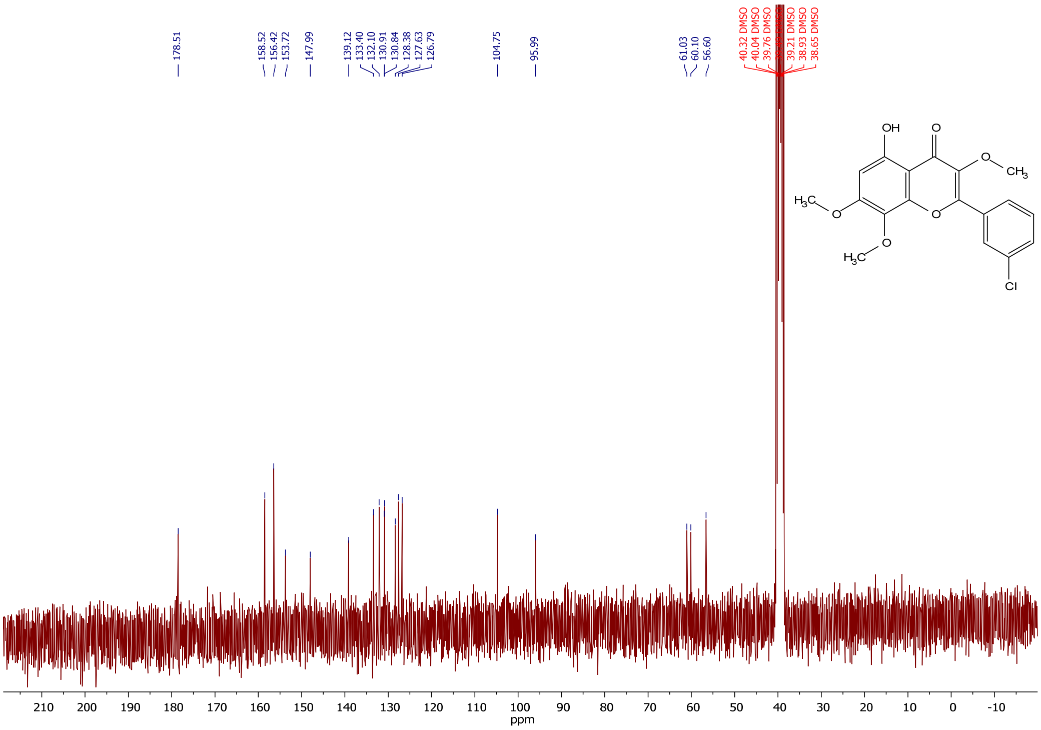

75 MHz, ^{13}C-NMR in DMSO-d_6

6. References

1. Christensen, H. Preparation of Salicylaldehydes via the Ortho-Lithio Derivatives of Methoxymethyl-Protected Phenols. *Synthetic Communications* **1975**, *5*, 65-78, doi:10.1080/00397917508063518.
2. Takeuchi, D.; Chiba, Y.; Takano, S.; Osakada, K. Double-Decker-Type Dinuclear Nickel Catalyst for Olefin Polymerization: Efficient Incorporation of Functional Co-monomers. *Angewandte Chemie International Edition* **2013**, *52*, 12536-12540, doi:https://doi.org/10.1002/anie.201307741.
3. Bischof, D.; Tripp, M.W.; Hofmann, P.E.; Ip, C.-H.; Ivlev, S.I.; Gerhard, M.; Koert, U.; Witte, G. Regioselective Fluorination of Acenes: Tailoring of Molecular Electronic Levels and Solid-State Properties. *Chemistry – A European Journal* **2022**, *28*, e202103653, doi:https://doi.org/10.1002/chem.202103653.
4. Badetti, E.; Lloveras, V.; Amadio, E.; Di Lorenzo, R.; Olivares-Marín, M.; Tesio, A.Y.; Zhang, S.; Pan, F.; Rissanen, K.; Veciana, J.; et al. Organic Polyradicals as Redox Mediators: Effect of Intramolecular Radical Interactions on Their Efficiency. *ACS Applied Materials & Interfaces* **2020**, *12*, 45968-45975, doi:10.1021/acsami.0c09386.
5. Ohno, S.; Qiu, J.; Miyazaki, R.; Aoyama, H.; Murai, K.; Hasegawa, J.-y.; Arisawa, M. Ni-Catalyzed Cycloisomerization between 3-Phenoxy Acrylic Acid Derivatives and Alkynes via Intramolecular Cleavage and Formation of the C–O Bond To Give 2,3-Disubstituted Benzofurans. *Organic Letters* **2019**, *21*, 8400-8403, doi:10.1021/acs.orglett.9b03170.
6. Li, L.; Wang, F.; Ni, C.; Hu, J. Synthesis of gem-Difluorocyclopropa(e)nes and O-, S-, N-, and P-Difluoromethylated Compounds with $TMSCF_2Br$. *Angewandte Chemie International Edition* **2013**, *52*, 12390-12394, doi:10.1002/anie.201306703.
7. *ROCS*, 3.4.2.1; OpenEye Scientific Software: Santa Fe, NM, 2020.
8. Rehberg, N.; Akone, H.S.; Ioerger, T.R.; Erlenkamp, G.; Daletos, G.; Gohlke, H.; Proksch, P.; Kalscheuer, R. Chlorflavonin Targets Acetohydroxyacid Synthase Catalytic Subunit IlvB1 for Synergistic Killing of Mycobacterium tuberculosis. *ACS Infect Dis* **2018**, *4*, 123-134, doi:10.1021/acsinfecdis.7b00055.
9. Homeyer, N.; Gohlke, H. FEW: A workflow tool for free energy calculations of ligand binding. *Journal of Computational Chemistry* **2013**, *34*, 965-973, doi:10.1002/jcc.23218.
10. Case, D.A.; Cheatham III, T.E.; Darden, T.; Gohlke, H.; Luo, R.; Merz Jr., K.M.; Onufriev, A.; Simmerling, C.; Wang, B.; Woods, R.J. The Amber biomolecular simulation programs. *Journal of Computational Chemistry* **2005**, *26*, 1668-1688, doi:10.1002/jcc.20290.
11. Wang, J.; Cieplak, P.; Kollman, P.A. How well does a restrained electrostatic potential (RESP) model perform in calculating conformational energies of organic and biological molecules? *Journal of Computational Chemistry* **2000**, *21*, 1049-1074, doi:10.1002/1096-987X(200009)21:12<1049::AID-JCC3>3.0.CO;2-F.
12. Kruskal, J.B. On the shortest spanning subtree of a graph and the traveling salesman problem. *Proceedings of the American Mathematical Society* **1956**, *7*, 48-50.
13. Hawkins, P.C.D.; Skillman, A.G.; Nicholls, A. Comparison of Shape-Matching and Docking as Virtual Screening Tools. *Journal of Medicinal Chemistry* **2007**, *50*, 74-82, doi:10.1021/jm0603365.
14. *QikProp*, Release 2021-4; Schrödinger: New York, NY, 2021.
15. Kaus, J.W.; Pierce, L.; Walker, R.C.; McCammont, J.A. Improving the Efficiency of Free Energy Calculations in the Amber Molecular Dynamics Package. *Journal of chemical theory and computation* **2013**, *9(9)*, doi:10.1021/ct400340s.

16. Homeyer, N.; Stoll, F.; Hillisch, A.; Gohlke, H. Binding Free Energy Calculations for Lead Optimization: Assessment of Their Accuracy in an Industrial Drug Design Context. *Journal of chemical theory and computation* **2014**, *10 (8)*, 3331-3344, doi:10.1021/ct5000296.
17. Ioannidis, H.; Drakopoulos, A.; Tzitzoglaki, C.; Homeyer, N.; Kolarov, F.; Gkeka, P.; Freudenberger, K.; Liolios, C.; Gauglitz, G.; Cournia, Z.; et al. Alchemical Free Energy Calculations and Isothermal Titration Calorimetry Measurements of Aminoadamantanes Bound to the Closed State of Influenza A/M2TM. *Journal of chemical information and modeling* **2016**, *56 5*, 862-876.
